# Integrative Analysis of Pharmacology and Transcriptomics Predicts Resveratrol Will Ameliorate Microplastics-Induced Lung Damage by Targeting Ccl2 and Esr1

**DOI:** 10.3390/toxics12120910

**Published:** 2024-12-14

**Authors:** Yadong Zhang, Jingyi Ren, Siqi Zhu, Zihao Guo, Huanting Pei, Xiaoya Sun, Jiarui Wu, Weijie Yang, Jinshi Zuo, Yuxia Ma

**Affiliations:** 1 Hebei Key Laboratory of Environment and Human Health, Department of Nutrition and Food Hygiene, School of Public Health, Hebei Medical University, Shijiazhuang 050017, China; yadong_z@163.com (Y.Z.); renj_yi@163.com (J.R.); zhus_q@163.com (S.Z.); huanting_p@163.com (H.P.); xiaoyasun1102@163.com (X.S.); 15133071859@163.com (J.W.); zuojinshi2022@163.com (J.Z.); 2 Department of Occupational and Environmental Health, School of Public Health, Xi’an Jiaotong University Health Science Center, Xi’an 710061, China; 18434009120@163.com; 3 College of Public Health, Hebei Medical University, Shijiazhuang 050017, China; 15803309112@163.com

**Keywords:** microplastics, lung injury, resveratrol, network pharmacology, transcriptomics

## Abstract

Background: Microplastics (MPs) are ubiquitous on earth, posing a growing threat to human health. Previous studies have shown that the lung is a primary organ for MPs exposure. Resveratrol (RES) is a common dietary polyphenol that exhibits anti-inflammatory and antioxidant effects. However, whether RES exerts a protective effect against MPs-induced lung damage is still unknown. Methods: The targets of RES were retrieved from five databases. Differentially expressed genes (DEGs) were identified through comprehensive bioinformatic analysis. Multiple algorithms were employed to screen for the core targets. Ultimately, molecular docking analysis and molecular dynamics (MD) simulations were utilized to confirm the binding affinity between RES and the core targets. Results: In total, 1235 DEGs were identified in the transcriptomes. After removing duplicates, a total of 739 RES targets were obtained from five databases, and 66 of these targets intersected with DEGs. The potential core targets (Esr1, Ccl2) were further identified through topological analysis and machine learning. These findings were subsequently verified by molecular docking and MD simulations. Conclusions: This study demonstrated that RES may mitigate lung injury induced by MPs by targeting Esr1 and Ccl2. Our research offers a novel perspective on the prevention and treatment of MPs-induced lung injury.

## 1. Introduction

The production of plastics has been increasing annually since the 1950s [1]. It is estimated that over 8 billion tons of plastics have been produced worldwide, and 75–80% have been poured into landfills or penetrated into the environment [2,3]. Out of the large amount of plastic waste generated, only a few are recycled and reused [4]. Plastics can disintegrate into microplastics (MPs) (<5 mm) when exposed to natural forces [5]. Current research mainly focuses on the adverse impacts of MPs on aquatic organisms [6]. It is shown that almost 700 aquatic organisms around the world are threatened by microplastic contamination [7,8]. MPs can easily float in the air and enter the body through the respiratory system due to their micro-level sizes [9]. There is a variety of environmental sources of atmospheric MPs, including road dust, sea spray, agricultural dust and dust near population centers [10]. Previous studies suggested that MPs have been detected in human lung tissue, with PP (particles and fibers) being the most common [11]. Textile fibers, a commonly identified type of MPs, have been widely found in environmental samples [12]. The textile fibers can be continuously released into the environment during abrasion, washing and drying of raiments [13]. There is some evidence that exposure to synthetic fibers is closely related to an elevated risk of lung diseases [14]. Therefore, it is necessary to study the mechanism of microplastic damage to lung tissue and to find effective intervention strategies.

Polyphenols, as secondary metabolites of plants, are widely found in most plants, fruits and vegetables [15]. It exhibited beneficial effects in the treatment of various pathological conditions [16,17,18]. Resveratrol (RES), one of the most widely researched polyphenols, can be conventionally found in various food sources, including grapes and blueberries [19,20]. Previous studies have reported that RES has been shown to confer protection in numerous animal models of acute lung injury (ALI) [21,22,23]. RES was able to attenuate the injury elicited by PM_2.5_ through modulation of Autophagy in human bronchial epithelial cells [24] and mouse lung tissue [25]. These findings indicated that RES may serve as a promising pharmaceutical agent for pulmonary diseases. Moreover, an additional benefit of RES is its favorable clinical safety profile [26]. In comparison to other polyphenolic compounds, RES has undergone more extensive investigation in clinical contexts, demonstrating a higher potential for application across diverse populations. However, it remains unclear whether RES is able to alleviate lung injury caused by MPs.

The concept of network pharmacology, initially introduced by Hopkins, involves the mapping of poly-pharmacology networks onto human disease-gene networks. This approach has demonstrated that drugs frequently interact with multiple targets and that drug targets are frequently associated with multiple diseases [27]. In this study, we hypothesize that RES may have beneficial effects in treating lung injury caused by MPs exposure. Network pharmacology, bioinformatics analysis, and the combination of multiple algorithms were used to explore the core therapeutic targets and molecular docking and molecular dynamics (MD) simulations were performed to further prove the binding affinity of RES with core therapeutic targets.

## 2. Materials and Methods

### 2.1. Bioinformatics Analysis

The gene expression dataset GSE238065 was retrieved from the GEO database (https://www.ncbi.nlm.nih.gov/geo/ (accessed on 4 June 2024)). The dataset contained RNA expression profiles from 4 normal lung epithelial cells and 4 lung epithelial cells exposed to 12 k nylon fibers. The analysis of differentially expressed genes (DEGs) was carried out using the R package DESeq2 (version 1.40.2). |log_2_FoldChange| > 1 and adjust *p* < 0.05 were determined as the screening criteria for DEGs. Principal component analysis (PCA) was performed using the FactoMineR package. The volcano plot and heatmap were generated by utilizing “ggplot2” and “pheatmap” R packages, respectively.

### 2.2. GO and KEGG Pathway Enrichment Analysis

“clusterProfiler” in the R software (version 4.0.3) was used to perform Gene Ontology (GO) functional annotation and Kyoto Encyclopedia of Genes and Genomes (KEGG) pathway analysis on the DEGs. Biochemical processes (BP), cell components (CC) and molecular functions (MF) were considered in GO enrichment analysis. Additionally, the *p*-value for statistical significance was set at 0.05.

### 2.3. Network Pharmacology Prediction

The potential targets related to RES were predicted using Traditional Chinese Medicine Systems Pharmacology (TCMSP, https://www.tcmsp-e.com/#/home (accessed on 22 April 2024)), Similarity Ensemble Approach (SEA, https://sea.bkslab.org/ (accessed on 22 April 2024)), Swiss Target Prediction (http://swisstargetprediction.ch/ (accessed on 22 April 2024)), TargetNet (http://targetnet.scbdd.com/home/index/ (accessed on 22 April 2024)) and Bioinformatics Analysis Tool for Molecular mechanism of Traditional Chinese Medicine (BATMAN-TCM, http://bionet.ncpsb.org.cn/batman-tcm/#/home (accessed on 22 April 2024)). All of these RES-related targets obtained from the five databases were identified and standardized by using the UniProt database. The targets were merged, and the duplicate values were removed. Subsequently, the RES-related targets and DEGs were intersected by using the “VennDiagram” package (1.6.20) in R software.

### 2.4. Protein-Protein Interaction (PPI) Network Analysis

The obtained intersection genes were inputted into the STRING database (https://cn.string-db.org/ (accessed on 4 June 2024)), specifying the species as “Mus musculus” in order to access PPI data. The results in TSV format were exported and imported into the Cytoscape 3.9.1 software for creating visualization. Then eight topology methods were conducted to analyze the network through CytoHubba, including MNC, Degree, EPC, BottleNeck, Closness, Radiality, Betweenness and Stress. The genes were sorted into descending order by their values, and the intersection of the top 10 genes was regarded as the hub genes.

### 2.5. Screening Core Genes by Machine Learning Method

The random forest algorithm was performed to identify the core genes involved in the intersection of genes. With the aim of ascertaining the lowest error rate and most stable tree number as the optimal parameter, each error rate for 1–150 trees was calculated, and the randomForest package was utilized to construct 150 random forest trees. The top 10 scoring genes, as recognized by the RF algorithm, were categorized as core genes.

### 2.6. Molecular Docking Analysis

The 2D molecular structure of RES (ligand) was retrieved from PubChem (https://pubchem.ncbi.nlm.nih.gov/ (accessed on 4 June 2024)), and the Alphafold structure of the core therapeutic targets (receptors) was gathered from the UniProt database (https://www.uniprot.org/ (accessed on 4 June 2024)). The ligand’s 2D structure was transformed into a 3D structure in ChemBio3D Ultra (version 21.0.0.28), and the molecular mechanics (MM2) force field was used to optimize energy. PyMoL software (version 2.5) was utilized to remove small-molecule ligands and water molecules. All ligand and receptor files were added hydrogen atoms and charges in AutoDock Tools (version 1.5.7) and exported as PDBQT format. Molecular docking simulations were executed using AutoDock Vina (version 1.1.2). Finally, the visualizations of the docking results were carried out by PyMoL software.

### 2.7. Molecular Dynamics (MD) Simulations Analysis

The Gromacs2022.3 software was employed for conducting MD simulations. In the preprocessing of small molecules, AmberTools22 was utilized to apply the general amber force field (GAFF), whereas Gaussian 16W was employed for the hydrogenation of small molecules and the calculation of RESP potential. Potential data will be added to the topology file of the molecular dynamics system. The simulation was conducted under static temperature conditions of 300 K and atmospheric pressure of 1 Bar. The Amber99sb-ildn force field was employed, with water molecules modeled using the Tip3p water model as the solvent, and the overall charge of the simulation system was balanced by the addition of a suitable quantity of Na^+^ ions. The simulation system utilizes the steepest descent method to minimize energy, followed by conducting 100,000 steps of isothermal isovolumic ensemble (NVT) equilibrium and isothermal isobaric ensemble (NPT) equilibrium, respectively, with a coupling constant of 0.1 ps and a duration of 100 ps. Finally, the free MD simulations were performed. The process included 5,000,000 steps, the step length was 2 fs, and the total duration was 100 ns. To investigate the stability of the complexes, all trajectories were further analyzed on the basis of root-mean-square deviation (RMSD), root-mean-square fluctuation (RMSF), radius of gyration (Rg) and hydrogen bonds.

## 3. Results

### 3.1. Identification of DEGs in Dataset

**Figure 1:**
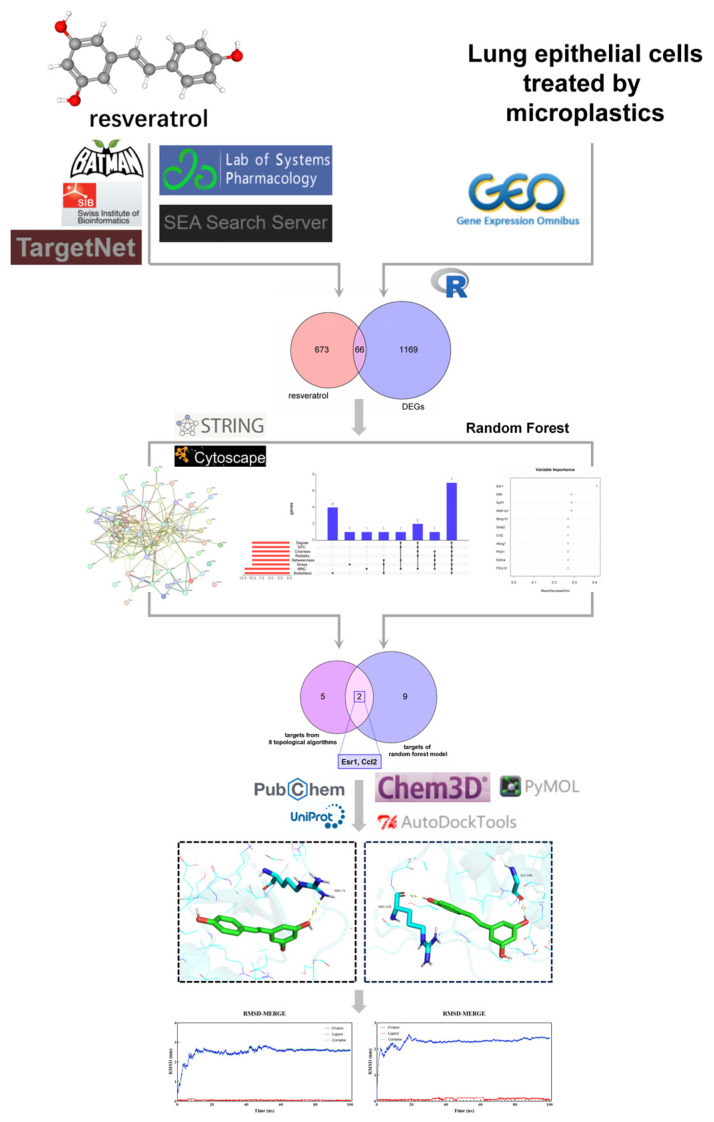
A detailed workflow of the network pharmacological investigation strategy for resveratrol in the treatment of lung injury caused by microplastics.

**Figure 2:**
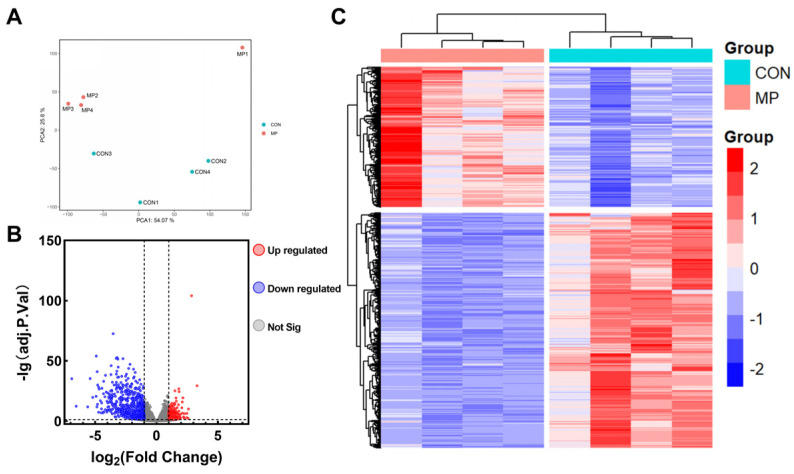
Transcriptomics data results between control group (CON) and microplastics-treated lung epithelial cells group (MP). (**A**) Principal component analysis (PCA) between CON and MP. (**B**) Volcano plots of the differentially expressed genes (DEGs). (**C**) Heatmap of the DEGs.

The workflow of our analysis is shown in Figure 1. The results of the unsupervised PCA (Figure 2A) showed an absolute separation between control group samples and exposure group samples. PC1 and PC2 represented 54.07% and 25.80%, respectively. The dataset was executed with differential expression analysis to obtain the DEGs using the “DESeq2” R package. The number of DEGs was delineated using volcano plots (Figure 2B). A total of 1235 DEGs were identified, meeting the set criterion (adjust *p* < 0.05 and |log2FoldChange| > 1). Among these DEGs, 461 genes were up-regulated and 774 genes were down-regulated. Subsequently, a heatmap of DEGs was displayed in Figure 2C. Cluster analysis unveils the expression of up-regulated and down-regulated genes across various samples. Red and blue symbolize up-regulation and down-regulation, respectively.

### 3.2. GO and KEGG Enrichment Analysis

**Figure 3:**
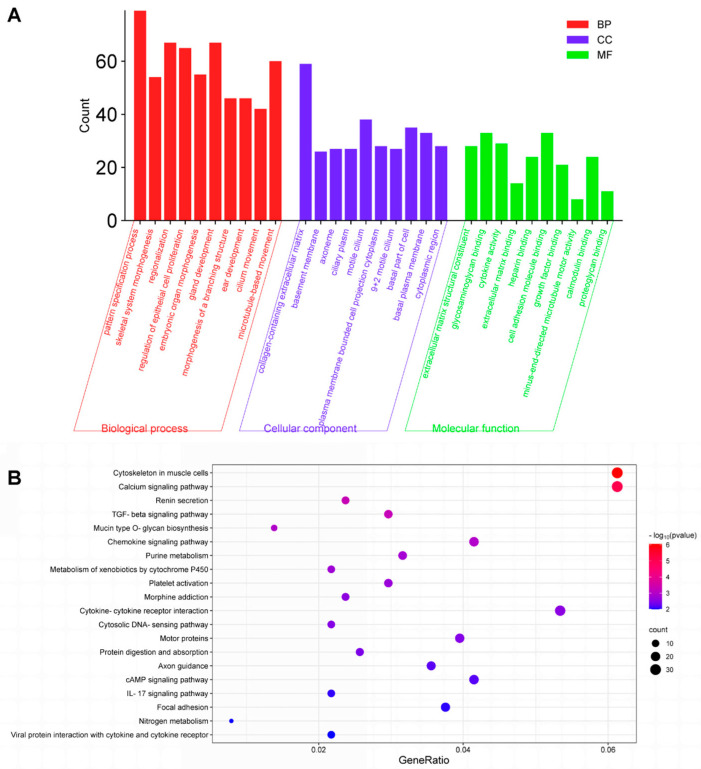
Enrichment analysis of the differentially expressed genes (DEGs) between control group (CON) and microplastics-treated lung epithelial cells group (MP). (**A**) GO analysis of DEGs. (**B**) KEGG analysis of DEGs.

The utilization of GO and KEGG enrichment analysis was used to unveil the functions of DEGs. As illustrated in Figure 3A, the top 10 items of BP, CC and MF were selected based on the *p*-value from the smallest to the largest, and a trichromatic bar chart was generated. In our study, a total of 1072 BPs, 42 CCs and 61 MFs (*p* < 0.05) were enriched across the DEGs. For BPs, the top-ranked enriched items were principally linked to the pattern specification process, skeletal system morphogenesis, regionalization, etc. As for CCs, the highest enriched items were primarily associated with collagen-containing extracellular matrix, basement membrane, axoneme, etc. Additionally, highly enriched MF terms consist of extracellular matrix structural constituent, glycosaminoglycan binding and cytokine activity. The KEGG enrichment analysis included 51 items. The top 20 results suggested that MPs exposure may induce multiple signaling pathway changes, such as calcium signaling pathway, TGF-beta signaling pathway, chemokine signaling pathway, etc. (Figure 3B). The details of all GO and KEGG enrichment analyses are shown in Tables S1 and S2.

### 3.3. Network Pharmacology

#### 3.3.1. The Collection of RES Targets

**Figure 4:**
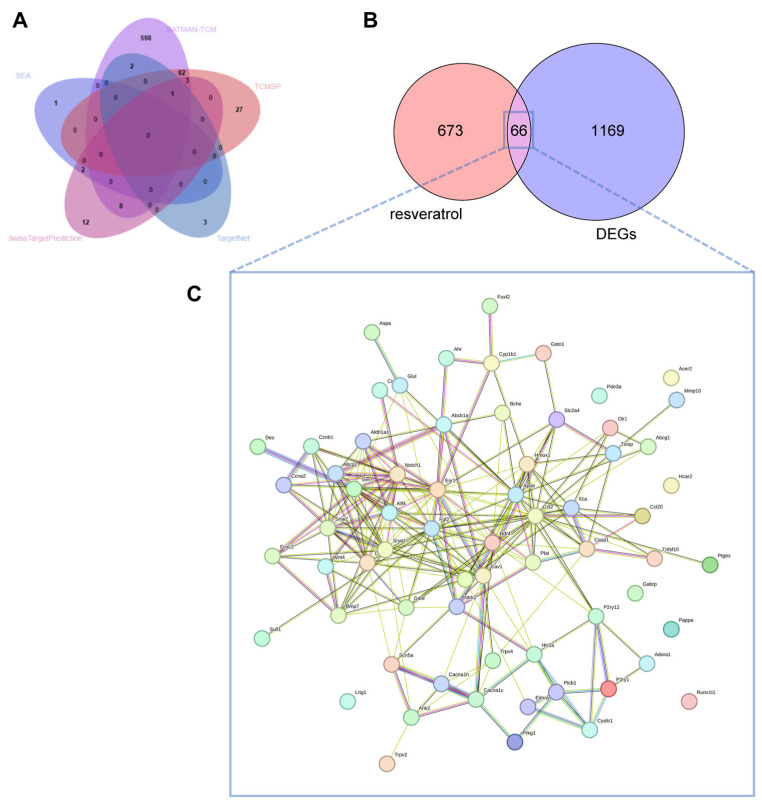
Targets of resveratrol (RES) and protein-protein interaction (PPI) network construction of intersection genes between RES’s targets and DEGs. (**A**) Venn graph showing the numbers of predicted RES targets. (**B**) Venn graph showing the intersection of RES’s targets and DEGs. (**C**) A total of 66 interaction targets were used for PPI network visualization. The distinct colors of the lines connecting the proteins in STRING denote the various types of interactions (databases, experiments, neighborhood, gene fusion, co-occurrence, text mining, co-expression, and homology).

We obtained a total of 739 target genes of RES from TCMSP, SEA, Swiss Target Prediction, TargetNet and BATMAN-TCM database, following the exclusion of partially overlapping targets. There were 113 targets from TCMSP, 3 targets from SEA, 26 targets from Swiss Target Prediction, 6 targets from TargetNet and 694 targets from BATMAN-TCM (Figure 4A).

#### 3.3.2. Identification of Potential Targets and PPI Network Construction

According to Figure 4B, 66 targets were predicted by combining the RES targets with the DEGs, namely, the targets of RES involved in the treatment of MPs-induced lung injury. Subsequently, the PPI network was constructed based on 66 potential therapeutic targets (Figure 4C). The greater the centrality of nodes within a network, the higher the frequency of their interactions with other nodes, suggesting their magnified importance within the overall network structure.

#### 3.3.3. GO and KEGG Analysis of Potential Therapeutic Targets

**Figure 5:**
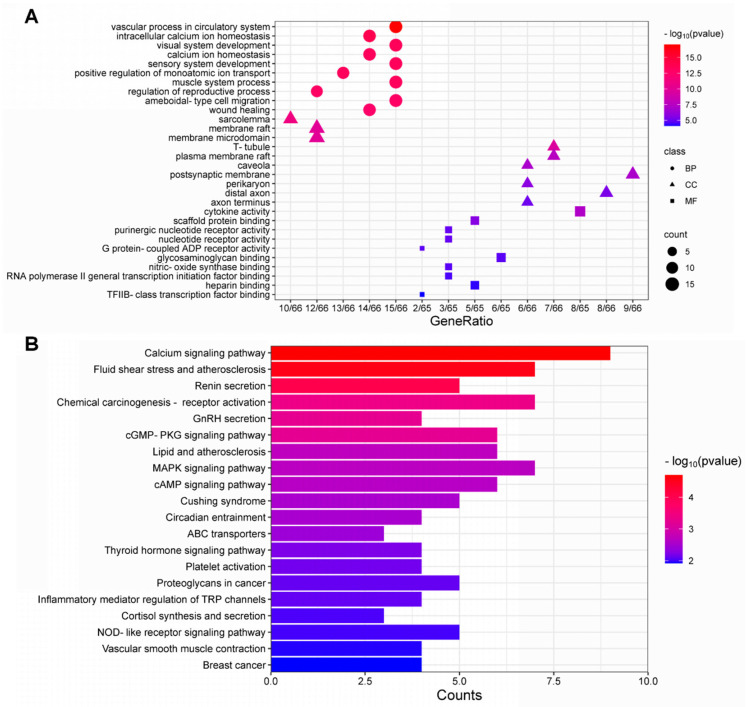
Enrichment analysis of the potential therapeutic targets. (**A**) GO analysis of the potential therapeutic targets. (**B**) KEGG analysis of the potential therapeutic targets.

GO enrichment analysis indicated that the potential therapeutic targets were involved in 1149 GO terms (*p* < 0.05), 1071 terms in BP, 38 terms in CC and 40 terms in MF. The top 10 ranked GO terms in BP, CC, and MF by *p*-value from the smallest to the largest are listed, which were identified as potential biological functions of therapeutic targets. A bubble plot was used to show the results of enrichment analysis (Figure 5A). Circles represent BPs, which revealed that the potential therapeutic targets chiefly regulated vascular processes in the circulatory system, intracellular calcium ion homeostasis and visual system development. Triangles represent CCs, and the targets were primarily associated with sarcolemma, membrane raft and membrane microdomain. Squares represent MFs, which were mainly concerned with cytokine activity, scaffold protein binding and purinergic nucleotide receptor activity. The results of KEGG enrichment analysis included 42 pathways (*p* < 0.05). Identically, the sorting of pathways was based on their *p*-value in ascending order. Among them, the calcium signaling pathway was remarkably important. Additionally, the top 20 pathways indicated that RES may exert a protective effect against lung injury caused by MPs through multiple signaling pathways, such as the cGMP-PKG signaling pathway, mitogen-activated protein kinase (MAPK) signaling pathway, cAMP signaling pathway, Thyroid hormone signaling pathway and NOD-like receptor signaling pathway (Figure 5B). The details of all GO and KEGG analyses were shown in Tables S3 and S4.

### 3.4. Identification of Hub Genes

**Figure 6:**
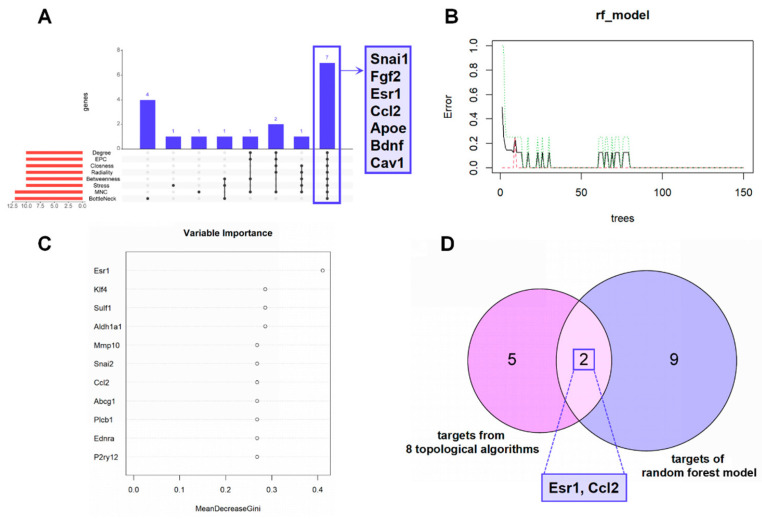
Screening of core potential therapeutic targets. (**A**) UpSet plot of 8 topological algorithms. (**B**) The correlation plot between the number of RF trees and model error. Each line illustrates the variation in error rate corresponding to the incremental addition of trees to the model. (**C**) The Gini coefficient method in a random forest classifier yielded the following results. The importance index is on the *x*-axis, and the genetic variable is on the *y*-axis. (**D**) Venn diagram showing the interaction between targets from 8 topological algorithms and screening targets of random forest model, which means the core potential therapeutic targets.

After constructing the PPI network, the significance of potential therapeutic targets within the interacting network was further assessed using CytoHubba in Cytoscape, employing algorithms including MNC, Degree, EPC, BottleNeck, Closness, Radiality, Betweenness and Stress. The targets were evaluated and ranked, with the top 10 targets based on their algorithm scores undergoing an intersection process. An UpSet diagram was utilized to show the number of genes. As shown in Figure 6A, a total of 7 genes were obtained from the intersection of 8 algorithms, including Snai1, Fgf2, Esr1, Ccl2, Apoe, Bdnf and Cav1. The detailed values are shown in Table 1.

### 3.5. Identification of Hub Genes by Machine Learning Methods

Random forest trees were utilized to select characteristic genes. The results suggested that when N = 14, the error was the smallest, as shown in Figure 6B. According to Figure 6C, Esr1 was the most significant variable, followed by Klf4, Sulf1, Aldh1a1, Mmp10, Snai2, Ccl2, Abcg1, Plcb1, Ednra and P2ry12. The importance score of these genes was calculated and shown in Table S5. Finally, a Venn diagram was implemented between topological algorithms and random forest results, and two genes (Esr1, Ccl2) were intersected (Figure 6D).

### 3.6. Molecular Docking

**Figure 7:**
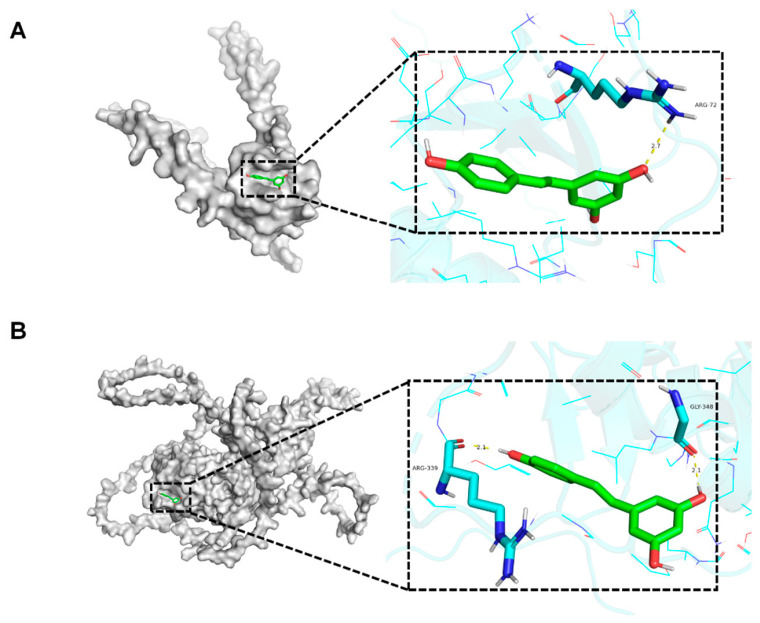
Molecular docking results of resveratrol (RES) and core potential therapeutic targets. (**A**) Binding modes of RES to Ccl2. (**B**) Binding modes of RES to Esr1.

Molecular docking analysis was utilized to further explore whether RES could interact with Esr1 and Ccl2. A binding energy value below 0 kcal/mol suggested the potential for spontaneous binding of the ligand molecule to the receptor target, while a binding energy value below −5 kcal/mol indicated a strong binding affinity. Our results suggested that the binding energy of RES with the hub genes was all below −5 kcal/mol. As shown in Figure 7A, RES formed 1 hydrogen bond with residue ARG-72 with a binding energy of −5.3 kcal/mol. In Figure 7B, the result suggested that RES showed a binding affinity to Esr1 with a binding energy of −6.4 kcal/mol and composed 2 hydrogen bonds with ARG-339 and GLY-348. Furthermore, we examined the structure-activity relationship (SAR), incorporating several additional polyphenols, including curcumin and silymarin, into the analysis. According to our docking results, the binding energy of curcumin with Ccl2 and Esr1 was −4.6 kcal/mol and −6.0 kcal/mol, respectively. The binding energy of silymarin with Ccl2 and Esr1 was −2.54 kcal/mol and −2.20 kcal/mol, respectively. The binding affinity of curcumin and silymarin for Ccl2 and Esr1 was found to be lower than that of RES for these two proteins. In comparison to the two aforementioned polyphenols, RES exhibits a smaller molecular weight, a greater number of phenolic hydroxyl groups, and the presence of ortho-phenolic hydroxyl groups, which collectively enhance its binding affinity to the core targets. The docking results associated with curcumin and silymarin are shown in Supplementary Figure S1.

### 3.7. Molecular Dynamics (MD) Simulations

**Figure 8:**
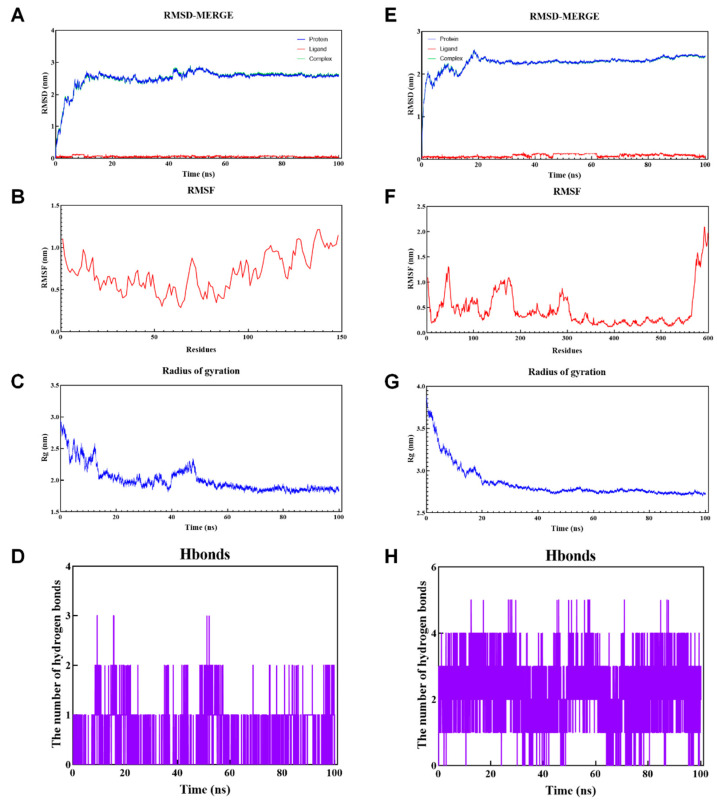
Molecular dynamics simulation analyses of the resveratrol-Ccl2 complex and resveratrol-Esr1 complex. (**A**) RMSD curve of resveratrol (red line), Ccl2 (blue line) and resveratrol-Ccl2 complex (green line). (**B**) RMSF curve of Ccl2. (**C**) Rg curve of Ccl2. (**D**) Hydrogen bonds of the resveratrol-Ccl2 complex. (**E**) RMSD curve of resveratrol (red line), Esr1 (blue line) and resveratrol-Esr1 complex (green line). (**F**) RMSF curve of Esr1. (**G**) Rg curve of Esr1. (**H**) Hydrogen bonds of the resveratrol-Esr1 complex.

To conduct a more thorough examination of the dynamic interaction process and the stability of binding between proteins and small ligands, we performed 100 ns MD simulations on each of the two docking complexes RES-Ccl2 and RES-Esr1. The RMSD serves as a quantitative indicator of the structural stability exhibited by protein-ligand complexes throughout the course of simulations. As the most universally used analytical method in MDs, the lower RMSD value suggested that the docking pose of this model was better. As shown in Figure 8, The RMSD values of both RES-Ccl2 and RES-Esr1 fluctuated relatively noticeably in the former 20 ns, showing an obvious upward trend. It was observed that both complexes stabilized after 20 ns (Figure 8A,E). With its holistic perspective, the RMSD values remained within a narrow range, suggesting that the interaction between protein and RES was characterized by stability. RMSFs provided insight into the impact of ligand binding on protein conformation. Lower RMSF values were typically observed in rigid structures, such as beta sheets and helix, while higher RMSF values were often obtained for turns and sheets. As shown in Figure 8B, the residues 65–76 of Ccl2 displayed greater flexibility. Additionally, the residues 43–53, 169–177, and 286–290 of Esr1 had greater residue flexibility in Figure 8F. Rg was frequently employed as a metric for determining the average radius of the mass weight within a simulation, as well as for assessing the proximity of molecules. Following the initial oscillation, both Ccl2 and Esr1 displayed stability, showing average Rg values around 1.85 nm and 2.75 nm, respectively (Figure 8C,G). Notably, lower Rg values showed more stability and compactness in the structure. During the simulation period, intermolecular interactions, specifically hydrogen bonds, between proteins and ligands were observed. The RES-Ccl2 complex demonstrated stable hydrogen bonds for 0–100 ns, with a maximum of three hydrogen bonds (Figure 8D). The RES-Esr1 complex revealed stable hydrogen bonds (1–4) in the MD simulation, with a maximum of five hydrogen bonds (Figure 8H). These hydrogen bonds are instrumental in the stability of the complex.

### 3.8. Construction of RES-Targets-Pathway-Disease Network

**Figure 9:**
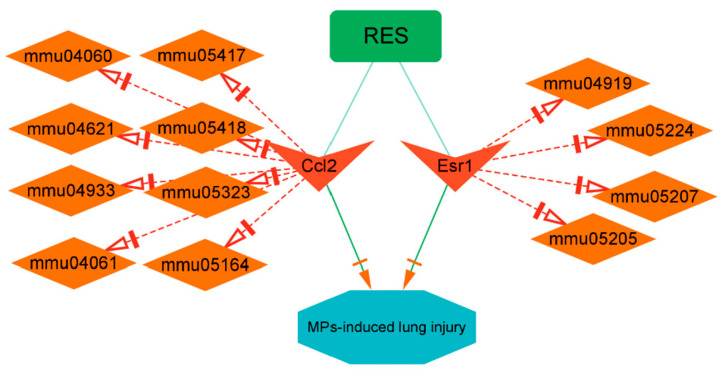
Collectively, integrated visualization network of resveratrol treated lung injury caused by microplastics was illustrated through the network pharmacology-based findings.

The Cytoscape software (version 3.9.1) was utilized for additional analysis. The presentation of an interaction diagram depicting core therapeutic target-related pathways is shown in Figure 9. The top three pathways related to Ccl2 were fluid shear stress and atherosclerosis (mmu05418), lipid and atherosclerosis (mmu05417), NOD-like receptor signaling pathway (mmu04621) and the top three pathways associated with Esr1 were chemical carcinogenesis-receptor activation (mmu05207), thyroid hormone signaling pathway (mmu04919) and proteoglycans in cancer (mmu05205).

## 4. Discussion

The recent advancements in microarray and sequencing technologies have significantly improved the ability to investigate the molecular landscape and potential mechanisms of emerging environmental pollutants. This progress is further bolstered by the growing use of integrated bioinformatic analysis, network pharmacology and machine learning tools to identify new genes, potential diagnostic and prognostic biomarkers, underlying mechanisms and therapeutic targets [28]. In this study, we propose a possible mechanism by which RES alleviates MPs-induced lung injury based on the integrative analysis of pharmacology and transcriptomics. The transcriptomic analysis revealed that exposure to MPs induced significant alterations in the transcriptional levels of Esr1 and Ccl2. Through the integration of network pharmacology and machine learning, we propose that RES may serve as a potential therapeutic agent for the treatment of MPs-induced lung injury, with Esr1 and Ccl2 identified as prospective targets. Molecular docking and MD simulations were used to further verify this finding.

In recent years, there has been a growing focus on the accumulation of plastic particles in the environment and within various organisms. The growing use of plastics and their slow biodegradation is a major concern for researchers and policymakers. The prevalence of micro- and nanoplastic contamination in freshwater and marine ecosystems, along with their associated toxicological ramifications, has been extensively documented. However, there is a paucity of research evaluating the effects of airborne MPs on terrestrial fauna. The current study mainly focused on the effects of MPs on the digestive system [29], but it is important to note that MPs can also be suspended in the air and have adverse health effects on multiple systems of the human body, especially on the respiratory system [30]. Airborne MPs had a wide range of sources, including synthetic textiles, synthetic rubber tire degradation, and urban dust [31]. In addition, there were several studies showed that approximately 7% of the overall microplastic contamination is attributed to aerial transfer from the ocean. A study by Zhu et al. [32] suggested that MPs exhibited the greatest degree of accumulation within the lungs, indicating that the lungs may be a major organ affected by MPs in the human body.

Currently, there is still a lack of research on the adverse health effects of MPs on the lungs. A previous study by Song et al. [33] indicated that both polyester and nylon fibers have the potential to hinder the differentiation of human and murine lung epithelial cells, with nylon demonstrating the greatest detrimental effect on airway epithelial cell differentiation. Considering the accumulation of MPs in the lungs through continuous and long-term inhalation, further investigation is warranted to explore the mechanism of MPs on lung injury. A recent study by Jin et al. [34] demonstrated that MPs cause senescence in mouse lungs and human lung epithelial cells via activating ROS signaling. Similar results were observed in A549 cells in a study by Chman Shahzadi et al. [35]. In our study, DEGs significantly enriched the chemokine signaling pathway, cytokine-cytokine receptor interaction and IL-17 signaling pathway. Similarly, the pathways linked to cytokine and chemokine were observed to be notably enriched in Zheng et al.’s research [36]. Ccl2, alternatively referred to as monocyte chemotactic protein 1 (Mcp1), is among the best-characterized chemokines within the C-C chemokine family, exhibiting a pivotal function in the inflammatory cascade. Notably, its impact on monocyte chemotaxis is of considerable importance [37]. In a study by Lai et al., overexpression of CCL2 in lung tissue was observed in a model of acute lung injury (ALI) induced by avian influenza A (H7N9). Furthermore, CCL2 deficiency was found to effectively mitigate H7N9-induced ALI in mice [38]. Our data showed the expression of Ccl2 was significantly upregulated in the MPs exposure group, indicating that Ccl2 may play an important role in MPs-induced lung inflammation. Esr1 is an estrogen receptor. The estrogen receptor plays critical roles in various physiological and pathological processes, including cell growth and differentiation, lipolysis, regulation of glucose metabolism, immune response and inflammation [39]. Estrogen receptors present in lung cells can promote cellular proliferation [40]. Previous studies have shown that the expression level of ESR1 in non-small cell lung cancer (NSCLC) tissue samples is significantly lower compared to adjacent normal tissue samples [41]. Moreover, the activation of ESR1 has been found to inhibit the proliferation of pulmonary myofibroblasts, thereby alleviating pulmonary fibrosis [42]. Our data in the MPs exposure group showed a notable reduction in Esr1 expression, suggesting that alterations in the estrogen signaling pathway could potentially play a crucial role in the deterioration of lung function.

RES, a polyphenolic phytonutrient, can be found naturally in various plants [43,44]. RES is acknowledged as a natural antioxidant with the ability to mitigate the effects of oxidative stress and inflammation [45,46]. A study by Aruna Kode et al. [47] suggested that RES can promote glutathione synthesis through the activation of Nrf2, thereby conferring protection against oxidative stress induced by cigarette smoke in human lung epithelial cells. Another study showed that RES could potentially alleviate lung epithelial cells’ inflammatory reaction [48]. However, whether RES could reduce lung inflammation and oxidative stress caused by MPs remains to be elucidated. According to our results, RES has the potential to alleviate MPs-induced lung injury, mainly manifested in its regulatory effects on Ccl2 and Esr1 proteins, which are potential targets for RES to exert pharmacological activity. The regulatory effects of RES on Ccl2 and Esr1 have also been confirmed in other studies. In the study of Gao et al. [49], Ccl2 was recognized as one of the key DEGs in the study of RES improving NSCLC. There is also evidence suggesting that RES can attenuate NSCLC by targeting Esr1. Currently, certain polyphenolic compounds, including pinostrobin [50] and maltol [51], have been utilized to mitigate testicular injury and liver injury in models induced by MPs. However, whether polyphenolic compounds can alleviate MPs-induced lung injury has not been reported. Therefore, the protective effect and mechanism of RES on MPs-induced lung injury deserve further in-depth research.

Molecular docking is a commonly utilized method for investigating the interaction between potential drug compounds and proteins in the field of computer-aided drug discovery and design. It is useful in exploring the details related to the interaction between ligands and proteins. With the aim of further validating the accuracy of our previous network pharmacology results, we performed molecular docking of RES with potential therapeutic targets. The results showed that RES had good molecular docking scores with both Ccl2 and Esr1. MD simulations are a sophisticated computational tool employed for the investigation of molecular movement and interactions. For further validation of the docking results, MD simulations were employed in which RMSD, RMSF, Rg and hydrogen binding analyses were performed. Two 100 ns MD simulations were performed for the RES-Ccl2 complex and RES-Esr1 complex. The results suggested that both complexes were stable during the whole simulation run.

From the standpoint of SAR, the biological effects of RES may be associated with its molecular structure. In contrast to berberine and curcumin, RES possesses the potential to traverse the blood–brain barrier and is not subject to elimination by the central nervous system [52]. Due to its small molecular weight and a higher number of phenolic hydroxyl groups, RES demonstrates a stronger molecular interaction profile, making it an ideal candidate for further investigation. Rüweler et al. [53] showed that in human glioma tumor cells, with the exception of RES, the majority of polyhydroxy-substituted RES analogs demonstrated cytotoxic effects upon the inhibition of cellular free radicals. RES is a well-studied natural polyphenol that has been shown to possess various biological functions, with particular emphasis on its potential therapeutic applications in lung diseases as highlighted in the literature [54]. Currently, a large amount of meta-analytic and experimental evidence supports the key role of RES in the intervention of pulmonary fibrosis, oxidative stress, inflammation and lung cancer [55,56,57,58]. In contrast, although other polyphenolic compounds, such as quercetin and green tea polyphenols, also exhibit antioxidant and anti-inflammatory properties, there is a relative paucity of research concerning their mechanisms of action and their specific effects on lung injury. Furthermore, numerous clinical trials have demonstrated that orally administered RES exhibits favorable bioavailability and safety profiles [59,60,61,62]. Therefore, as a potential therapeutic candidate, RES not only exhibits positive effects in experimental studies but also holds promising clinical prospects. In addition, the alterations to the hydroxyl groups, benzene rings, and double bonds of RES can significantly enhance its biological activity. Future advancements in the development of RES-based pharmaceuticals hold the potential to further augment its pharmacological efficacy.

However, our study is subject to certain limitations. First, the forecasted target data were sourced from databases, necessitating regular updates to maintain data accuracy. Second, current network pharmacology analyses predominantly focus on qualitative assessments of potential drug targets in diseases, whereas the quantitative evaluation of drug dosage-related effects remains inadequate. Third, additional researches, including animal experiments or cell experiments, are required to substantiate the conclusions drawn from this study.

## 5. Conclusions

In summary, our research first revealed that RES has the potential to alleviate MPs-induced lung damage by targeting Ccl2 and Esr1 based on the integrative analysis of pharmacology and transcriptomics. Molecular docking and MD simulations were used to validate intermolecular interactions between targets and RES. Given the intricate mechanisms underlying MPs-induced lung injury, it is imperative that the roles of Esr1 and Ccl2 be further investigated through transcriptomic and epigenetic approaches in future research. Meanwhile, we will further validate the feasibility of RES in reducing MPs-induced lung injury via regulating Esr1 and Ccl2 through in vivo and in vitro experiments.

## Figures and Tables

**Table 1:** Detailed information of the hub genes.

Protein Names	Gene Names	Topological Algorithms Score							
		MNC	Degree	EPC	BottleNeck	Closness	Radiality	Betweenness	Stress
Estrogen receptor	Esr1	25	50	27.29	10	40.5	4.43103	533.37597	21,400
Fibroblast growth factor 2	Fgf2	22	46	27.489	5	39.33333	4.37931	322.89579	12,856
C-C motif chemokine 2	Ccl2	20	44	26.404	6	39.16667	4.39655	609.90356	20,224
Apolipoprotein E	Apoe	18	38	25.722	5	37.75	4.34483	362.13894	12,424
Brain-derived neurotrophic factor	Bdnf	18	36	25.801	11	37.16667	4.32759	348.93476	17,216
Zinc finger protein SNAI1	Snai1	15	32	25.864	5	35.33333	4.2069	207.81551	9824
Caveolin-1	Cav1	15	32	24.692	6	36.33333	4.31034	397.20374	18,152

## Data Availability

The data generated during the current study are available from the corresponding author on reasonable request.

## Supplementary Materials

The following supporting information can be downloaded at: https://www.mdpi.com/article/10.3390/toxics12120910/s1, Figure S1: The docking results of curcumin, silymarin and core targets, Table S1: GO analysis of DEGs, Table S2: KEGG analysis of DEGs, Table S3: GO analysis of potential therapeutic targets, Table S4: KEGG analysis of potential therapeutic targets, Table S5: Ranking of variable importance as identified by the random forest model.
