# Supplementary material for: Integrative Analysis of Pharmacology and Transcriptomics Predicts Resveratrol Will Ameliorate Microplastics-Induced Lung Damage by Targeting Ccl2 and Esr1
Source: Toxics. 2024 Dec 14;12(12):910. doi: 10.3390/toxics12120910 (PMC11728634; doi:10.3390/toxics12120910)
Supplement: Supplementary file 1 [file toxics-12-00910-s001.zip › supplementary figure.pdf]

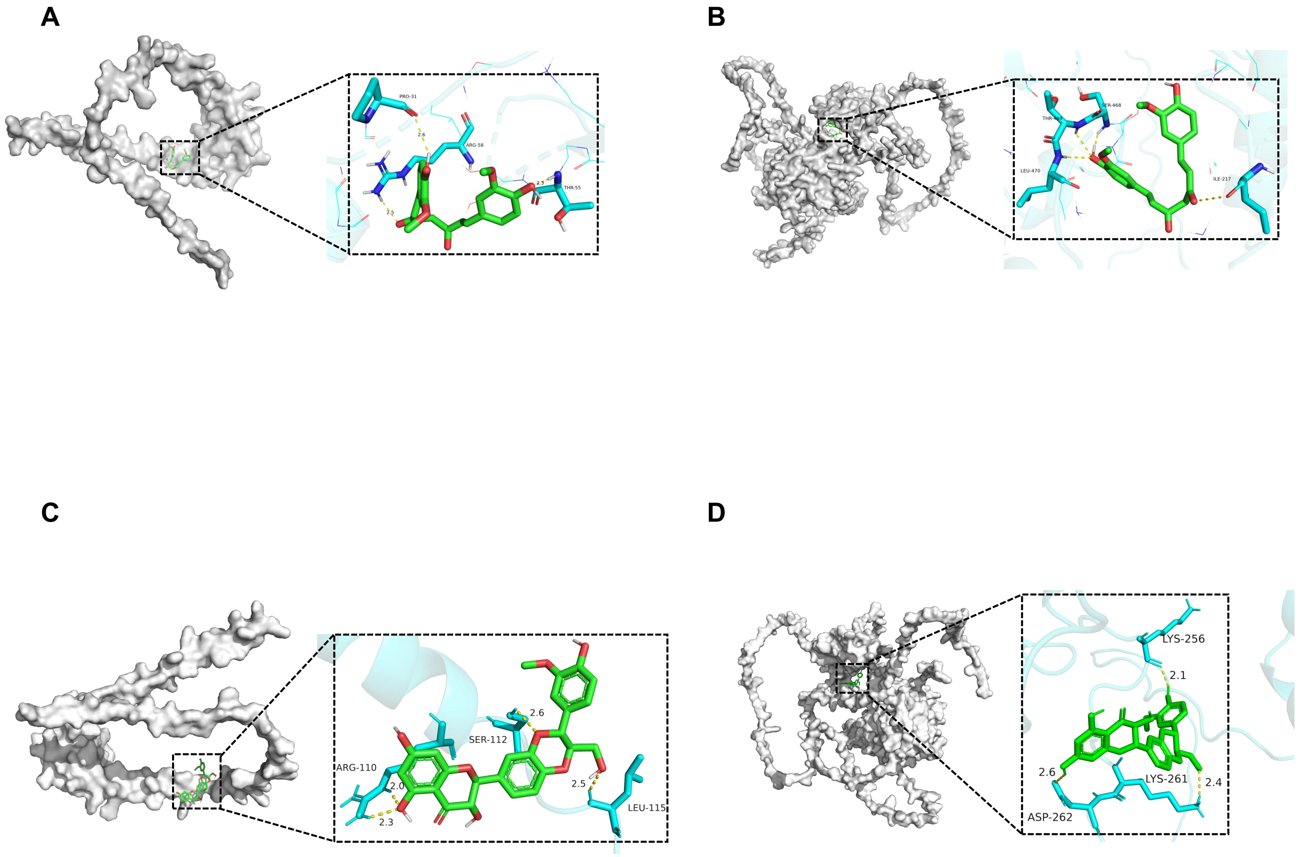

Figure S1. The docking results of curcumin, silymarin and core targets. **A** Binding modes of curcumin to Ccl2. **B** Binding modes of curcumin to Esr1. **C** Binding modes of silymarin to Ccl2. **D** Binding modes of silymarin to Esr1.
