# Supplementary material for: Integrative Analysis of Pharmacology and Transcriptomics Predicts Resveratrol Will Ameliorate Microplastics-Induced Lung Damage by Targeting Ccl2 and Esr1
Source: Toxics. 2024 Dec 14;12(12):910. doi: 10.3390/toxics12120910 (PMC11728634; doi:10.3390/toxics12120910)
Supplement: Supplementary file 1 [file toxics-12-00910-s001.zip › supplementary materials.pdf]

Table S1 GO analysis of DEGs

ID	Category	Term	Count	pvalue
GO:0007389	BP	pattern specification process	79	3.34E-27
GO:0048705	BP	skeletal system morphogenesis	54	4.78E-24
GO:0003002	BP	regionalization	67	2.02E-21
GO:0050678	BP	regulation of epithelial cell proliferation	65	2.74E-21
GO:0048562	BP	embryonic organ morphogenesis	55	8.39E-20
GO:0048732	BP	gland development	67	1.12E-19
GO:0001763	BP	morphogenesis of a branching structure	46	8.31E-19
GO:0043583	BP	ear development	46	2.22E-18
GO:0003341	BP	cilium movement	42	1.56E-17
GO:0007018	BP	microtubule-based movement	60	3.02E-17
GO:0001667	BP	ameboidal-type cell migration	62	3.25E-17
GO:0061448	BP	connective tissue development	49	3.26E-17
GO:0090596	BP	sensory organ morphogenesis	50	3.35E-17
GO:0061138	BP	morphogenesis of a branching epithelium	42	3.6E-17
GO:0051216	BP	cartilage development	41	4E-17
GO:0001503	BP	ossification	58	9.03E-17
GO:0048706	BP	embryonic skeletal system development	33	2.16E-16
GO:0030198	BP	extracellular matrix organization	49	2.36E-16
GO:0060485	BP	mesenchyme development	48	2.37E-16
GO:0043062	BP	extracellular structure organization	49	3.05E-16
GO:0045229	BP	external encapsulating structure organization	49	3.05E-16
GO:0050767	BP	regulation of neurogenesis	61	3.5E-16
GO:0048754	BP	branching morphogenesis of an epithelial tube	37	4.35E-16
GO:0048704	BP	embryonic skeletal system morphogenesis	28	1.06E-15
GO:0045664	BP	regulation of neuron differentiation	41	2.05E-15
GO:0001578	BP	microtubule bundle formation	30	2.3E-15
GO:0050679	BP	positive regulation of epithelial cell proliferation	40	3.87E-15
GO:0060562	BP	epithelial tube morphogenesis	53	4.05E-15
GO:0040013	BP	negative regulation of locomotion	50	5.02E-15
GO:0048839	BP	inner ear development	38	1.02E-14
GO:0043588	BP	skin development	47	1.43E-14
GO:0050900	BP	leukocyte migration	51	2.04E-14
GO:0048762	BP	mesenchymal cell differentiation	40	2.14E-14
GO:0007409	BP	axonogenesis	59	2.55E-14
GO:0030900	BP	forebrain development	53	2.84E-14
GO:2000027	BP	regulation of animal organ morphogenesis	29	3.24E-14
GO:0035082	BP	axoneme assembly	25	3.42E-14
GO:0042060	BP	wound healing	51	3.79E-14
GO:1901342	BP	regulation of vasculature development	44	9.01E-14
GO:0008544	BP	epidermis development	50	9.83E-14
GO:0031214	BP	biomineral tissue development	33	1.03E-13
GO:0045165	BP	cell fate commitment	43	1.94E-13

GO:0050769	BP	positive regulation of neurogenesis	44	2.51E-13
GO:0009952	BP	anterior/posterior pattern specification	37	2.91E-13
GO:2000177	BP	regulation of neural precursor cell proliferation	27	3.19E-13
GO:0150063	BP	visual system development	52	3.64E-13
GO:0016055	BP	Wnt signaling pathway	54	3.93E-13
GO:0030856	BP	regulation of epithelial cell differentiation	31	4.12E-13
GO:0198738	BP	cell-cell signaling by wnt	54	4.68E-13
GO:0048863	BP	stem cell differentiation	41	5.04E-13
GO:0048880	BP	sensory system development	52	5.24E-13
GO:2000146	BP	negative regulation of cell motility	44	5.41E-13
GO:0042476	BP	odontogenesis	27	5.82E-13
GO:0001654	BP	eye development	51	9.91E-13
GO:0045765	BP	regulation of angiogenesis	42	1.03E-12
GO:0050920	BP	regulation of chemotaxis	36	1.23E-12
GO:0032102	BP	negative regulation of response to external stimulus	51	1.3E-12
GO:0042471	BP	ear morphogenesis	28	1.47E-12
GO:0030282	BP	bone mineralization	27	1.52E-12
GO:0043010	BP	camera-type eye development	47	2E-12
GO:0048771	BP	tissue remodeling	34	2E-12
GO:0001649	BP	osteoblast differentiation	35	2.06E-12
GO:0051962	BP	positive regulation of nervous system development	47	2.2E-12
GO:0030336	BP	negative regulation of cell migration	42	2.21E-12
GO:1905330	BP	regulation of morphogenesis of an epithelium	20	2.91E-12
GO:0090287	BP	regulation of cellular response to growth factor stimulus	42	5.13E-12
GO:0001822	BP	kidney development	43	6.7E-12
GO:0072001	BP	renal system development	44	8.49E-12
GO:0052547	BP	regulation of peptidase activity	52	8.87E-12
GO:0002685	BP	regulation of leukocyte migration	35	9.53E-12
GO:0022612	BP	gland morphogenesis	28	1.31E-11
GO:0061041	BP	regulation of wound healing	26	1.64E-11
GO:0043542	BP	endothelial cell migration	34	2.06E-11
GO:0030111	BP	regulation of Wnt signaling pathway	41	2.2E-11
GO:0060326	BP	cell chemotaxis	40	2.29E-11
GO:0050777	BP	negative regulation of immune response	32	2.42E-11
GO:1902692	BP	regulation of neuroblast proliferation	17	2.79E-11
GO:0072006	BP	nephron development	28	2.85E-11
GO:0070371	BP	ERK1 and ERK2 cascade	43	3.42E-11
GO:0033002	BP	muscle cell proliferation	37	3.68E-11
GO:0031589	BP	cell-substrate adhesion	44	4.11E-11
GO:0001936	BP	regulation of endothelial cell proliferation	27	4.57E-11
GO:0061351	BP	neural precursor cell proliferation	32	4.59E-11
GO:1903034	BP	regulation of response to wounding	29	4.9E-11
GO:0090130	BP	tissue migration	41	5.8E-11
GO:0043534	BP	blood vessel endothelial cell migration	24	6.39E-11

GO:0042475	BP	odontogenesis of dentin-containing tooth	21	7.25E-11
GO:0042063	BP	gliogenesis	44	9.08E-11
GO:0014706	BP	striated muscle tissue development	38	1.02E-10
GO:0001935	BP	endothelial cell proliferation	28	1.07E-10
GO:0060541	BP	respiratory system development	36	1.08E-10
GO:0010631	BP	epithelial cell migration	40	1.46E-10
GO:0070372	BP	regulation of ERK1 and ERK2 cascade	40	1.75E-10
GO:0090132	BP	epithelium migration	40	1.75E-10
GO:0060348	BP	bone development	35	1.86E-10
GO:0007162	BP	negative regulation of cell adhesion	39	2.29E-10
GO:0010594	BP	regulation of endothelial cell migration	28	2.44E-10
GO:0048844	BP	artery morphogenesis	19	3.05E-10
GO:0044458	BP	motile cilium assembly	18	3.07E-10
GO:0001823	BP	mesonephros development	22	3.52E-10
GO:0060688	BP	regulation of morphogenesis of a branching structure	17	3.62E-10
GO:0001818	BP	negative regulation of cytokine production	37	3.85E-10
GO:0003351	BP	epithelial cilium movement involved in extracellular fluid movement	14	4.25E-10
GO:0048663	BP	neuron fate commitment	19	4.72E-10
GO:0010959	BP	regulation of metal ion transport	48	5.58E-10
GO:0045667	BP	regulation of osteoblast differentiation	26	5.83E-10
GO:0001938	BP	positive regulation of endothelial cell proliferation	21	6.3E-10
GO:0001657	BP	ureteric bud development	21	7.56E-10
GO:0070167	BP	regulation of biomineral tissue development	21	7.56E-10
GO:0007288	BP	sperm axoneme assembly	12	7.56E-10
GO:0060840	BP	artery development	22	9.92E-10
GO:0007405	BP	neuroblast proliferation	19	1.09E-09
GO:0007411	BP	axon guidance	33	1.14E-09
GO:0006858	BP	extracellular transport	14	1.17E-09
GO:0002062	BP	chondrocyte differentiation	22	1.17E-09
GO:0042472	BP	inner ear morphogenesis	22	1.17E-09
GO:0072009	BP	nephron epithelium development	22	1.17E-09
GO:0097485	BP	neuron projection guidance	33	1.26E-09
GO:0072163	BP	mesonephric epithelium development	21	1.29E-09
GO:0072164	BP	mesonephric tubule development	21	1.29E-09
GO:0070374	BP	positive regulation of ERK1 and ERK2 cascade	31	1.3E-09
GO:0001539	BP	cilium or flagellum-dependent cell motility	27	1.33E-09
GO:0060285	BP	cilium-dependent cell motility	27	1.33E-09
GO:0097529	BP	myeloid leukocyte migration	32	1.44E-09
GO:0072073	BP	kidney epithelium development	25	1.46E-09
GO:0031346	BP	positive regulation of cell projection organization	47	1.56E-09
GO:0001656	BP	metanephros development	19	1.62E-09
GO:0010463	BP	mesenchymal cell proliferation	15	1.65E-09
GO:0060294	BP	cilium movement involved in cell motility	26	2.53E-09
GO:0006816	BP	calcium ion transport	46	2.83E-09

GO:2000179	BP	positive regulation of neural precursor cell proliferation	17	3.08E-09
GO:0060070	BP	canonical Wnt signaling pathway	36	3.18E-09
GO:0044782	BP	cilium organization	43	3.26E-09
GO:0120316	BP	sperm flagellum assembly	13	3.64E-09
GO:0003205	BP	cardiac chamber development	28	3.74E-09
GO:0002040	BP	sprouting angiogenesis	22	4.14E-09
GO:0045666	BP	positive regulation of neuron differentiation	21	4.14E-09
GO:0043535	BP	regulation of blood vessel endothelial cell migration	19	4.17E-09
GO:0010466	BP	negative regulation of peptidase activity	33	4.52E-09
GO:0060993	BP	kidney morphogenesis	20	4.65E-09
GO:0045669	BP	positive regulation of osteoblast differentiation	17	4.73E-09
GO:0052548	BP	regulation of endopeptidase activity	40	4.78E-09
GO:0019221	BP	cytokine-mediated signaling pathway	44	4.93E-09
GO:0090183	BP	regulation of kidney development	12	5.61E-09
GO:0045444	BP	fat cell differentiation	33	5.98E-09
GO:0030500	BP	regulation of bone mineralization	18	6.13E-09
GO:0001655	BP	urogenital system development	16	6.28E-09
GO:0010810	BP	regulation of cell-substrate adhesion	30	6.42E-09
GO:0035107	BP	appendage morphogenesis	26	6.8E-09
GO:0035108	BP	limb morphogenesis	26	6.8E-09
GO:0009913	BP	epidermal cell differentiation	31	6.82E-09
GO:0030326	BP	embryonic limb morphogenesis	23	7.96E-09
GO:0035113	BP	embryonic appendage morphogenesis	23	7.96E-09
GO:0003012	BP	muscle system process	44	8.05E-09
GO:0042445	BP	hormone metabolic process	31	8.29E-09
GO:0045766	BP	positive regulation of angiogenesis	26	8.62E-09
GO:1904018	BP	positive regulation of vasculature development	26	8.62E-09
GO:0060349	BP	bone morphogenesis	20	9.01E-09
GO:0032652	BP	regulation of interleukin-1 production	21	9.02E-09
GO:0007178	BP	transmembrane receptor protein serine/threonine kinase signaling pathway	42	1.06E-08
GO:0010632	BP	regulation of epithelial cell migration	31	1.11E-08
GO:0014033	BP	neural crest cell differentiation	19	1.19E-08
GO:0048592	BP	eye morphogenesis	26	1.37E-08
GO:0048736	BP	appendage development	28	1.39E-08
GO:0060173	BP	limb development	28	1.39E-08
GO:0009914	BP	hormone transport	42	1.41E-08
GO:0034329	BP	cell junction assembly	46	1.51E-08
GO:0050878	BP	regulation of body fluid levels	38	1.62E-08
GO:0060675	BP	ureteric bud morphogenesis	16	1.84E-08
GO:1904888	BP	cranial skeletal system development	17	1.91E-08
GO:0072080	BP	nephron tubule development	19	1.96E-08
GO:0030323	BP	respiratory tube development	30	2.1E-08
GO:1905332	BP	positive regulation of morphogenesis of an epithelium	12	2.17E-08
GO:0032612	BP	interleukin-1 production	21	2.17E-08

GO:0042474	BP	middle ear morphogenesis	10	2.18E-08
GO:0045598	BP	regulation of fat cell differentiation	23	2.28E-08
GO:0030879	BP	mammary gland development	24	2.49E-08
GO:0097722	BP	sperm motility	24	2.49E-08
GO:0030203	BP	glycosaminoglycan metabolic process	19	2.7E-08
GO:0006936	BP	muscle contraction	35	3.16E-08
GO:0010721	BP	negative regulation of cell development	35	3.16E-08
GO:0050890	BP	cognition	38	3.42E-08
GO:0060560	BP	developmental growth involved in morphogenesis	33	3.49E-08
GO:0120197	BP	mucociliary clearance	7	3.66E-08
GO:0003007	BP	heart morphogenesis	33	3.79E-08
GO:0072171	BP	mesonephric tubule morphogenesis	16	4.07E-08
GO:0006022	BP	aminoglycan metabolic process	20	4.11E-08
GO:0032963	BP	collagen metabolic process	20	4.11E-08
GO:0048864	BP	stem cell development	18	4.26E-08
GO:0060828	BP	regulation of canonical Wnt signaling pathway	30	4.41E-08
GO:0045861	BP	negative regulation of proteolysis	38	4.56E-08
GO:0014032	BP	neural crest cell development	17	4.72E-08
GO:0001837	BP	epithelial to mesenchymal transition	23	4.78E-08
GO:0030317	BP	flagellated sperm motility	23	4.78E-08
GO:0002052	BP	positive regulation of neuroblast proliferation	11	4.91E-08
GO:0035924	BP	cellular response to vascular endothelial growth factor stimulus	14	5.09E-08
GO:0007219	BP	Notch signaling pathway	25	5.12E-08
GO:0051346	BP	negative regulation of hydrolase activity	38	5.26E-08
GO:0098773	BP	skin epidermis development	23	5.39E-08
GO:0072028	BP	nephron morphogenesis	17	5.61E-08
GO:2000241	BP	regulation of reproductive process	28	5.67E-08
GO:0030324	BP	lung development	29	5.77E-08
GO:0061326	BP	renal tubule development	19	5.8E-08
GO:0003197	BP	endocardial cushion development	13	6.32E-08
GO:0061005	BP	cell differentiation involved in kidney development	13	6.32E-08
GO:0003018	BP	vascular process in circulatory system	29	6.33E-08
GO:0030199	BP	collagen fibril organization	14	6.38E-08
GO:0046879	BP	hormone secretion	40	6.43E-08
GO:0070098	BP	chemokine-mediated signaling pathway	15	7.1E-08
GO:0048608	BP	reproductive structure development	35	7.4E-08
GO:0035282	BP	segmentation	19	7.78E-08
GO:0061333	BP	renal tubule morphogenesis	17	7.87E-08
GO:0030850	BP	prostate gland development	13	8.08E-08
GO:0060271	BP	cilium assembly	38	8.59E-08
GO:0030595	BP	leukocyte chemotaxis	28	9.07E-08
GO:0045216	BP	cell-cell junction organization	28	9.07E-08
GO:0021675	BP	nerve development	17	9.28E-08
GO:1902742	BP	apoptotic process involved in development	12	9.34E-08

GO:0061458	BP	reproductive system development	35	9.96E-08
GO:0021537	BP	telencephalon development	31	1.03E-07
GO:0002042	BP	cell migration involved in sprouting angiogenesis	13	1.03E-07
GO:0003206	BP	cardiac chamber morphogenesis	22	1.03E-07
GO:0061035	BP	regulation of cartilage development	15	1.05E-07
GO:0042692	BP	muscle cell differentiation	43	1.07E-07
GO:0032330	BP	regulation of chondrocyte differentiation	13	1.3E-07
GO:0099111	BP	microtubule-based transport	27	1.3E-07
GO:0071772	BP	response to BMP	25	1.32E-07
GO:0071773	BP	cellular response to BMP stimulus	25	1.32E-07
GO:0051924	BP	regulation of calcium ion transport	32	1.35E-07
GO:0030177	BP	positive regulation of Wnt signaling pathway	21	1.38E-07
GO:0048565	BP	digestive tract development	20	1.4E-07
GO:0048593	BP	camera-type eye morphogenesis	22	1.47E-07
GO:0030099	BP	myeloid cell differentiation	44	1.5E-07
GO:0007043	BP	cell-cell junction assembly	23	1.51E-07
GO:0030178	BP	negative regulation of Wnt signaling pathway	23	1.51E-07
GO:0030278	BP	regulation of ossification	21	1.56E-07
GO:0055123	BP	digestive system development	21	1.56E-07
GO:0002218	BP	activation of innate immune response	28	1.56E-07
GO:0045604	BP	regulation of epidermal cell differentiation	13	1.63E-07
GO:0072132	BP	mesenchyme morphogenesis	13	1.63E-07
GO:0090049	BP	regulation of cell migration involved in sprouting angiogenesis	11	1.71E-07
GO:0072078	BP	nephron tubule morphogenesis	16	1.71E-07
GO:0044344	BP	cellular response to fibroblast growth factor stimulus	17	1.76E-07
GO:0031349	BP	positive regulation of defense response	42	1.77E-07
GO:0016358	BP	dendrite development	34	1.96E-07
GO:0007188	BP	adenylate cyclase-modulating G protein-coupled receptor signaling pathway	27	2.06E-07
GO:0001658	BP	branching involved in ureteric bud morphogenesis	14	2.23E-07
GO:0048709	BP	oligodendrocyte differentiation	19	2.35E-07
GO:0003151	BP	outflow tract morphogenesis	16	2.38E-07
GO:0072088	BP	nephron epithelium morphogenesis	16	2.38E-07
GO:0050727	BP	regulation of inflammatory response	37	2.48E-07
GO:0021543	BP	pallium development	23	2.58E-07
GO:0032732	BP	positive regulation of interleukin-1 production	15	2.63E-07
GO:0071774	BP	response to fibroblast growth factor	17	2.77E-07
GO:0048566	BP	embryonic digestive tract development	10	2.96E-07
GO:0035850	BP	epithelial cell differentiation involved in kidney development	11	3E-07
GO:0060021	BP	roof of mouth development	17	3.22E-07
GO:0043270	BP	positive regulation of monoatomic ion transport	30	3.35E-07
GO:0042303	BP	molting cycle	20	3.37E-07
GO:0042633	BP	hair cycle	20	3.37E-07
GO:0002833	BP	positive regulation of response to biotic stimulus	34	3.46E-07
GO:0009410	BP	response to xenobiotic stimulus	34	3.46E-07

GO:0071695	BP	anatomical structure maturation	32	3.62E-07
GO:0032651	BP	regulation of interleukin-1 beta production	17	3.72E-07
GO:0001764	BP	neuron migration	25	3.85E-07
GO:0045862	BP	positive regulation of proteolysis	37	3.9E-07
GO:0048738	BP	cardiac muscle tissue development	30	3.92E-07
GO:0060512	BP	prostate gland morphogenesis	10	4.05E-07
GO:0014013	BP	regulation of gliogenesis	20	4.28E-07
GO:0072089	BP	stem cell proliferation	20	4.28E-07
GO:1990868	BP	response to chemokine	15	4.4E-07
GO:1990869	BP	cellular response to chemokine	15	4.4E-07
GO:0010977	BP	negative regulation of neuron projection development	22	4.45E-07
GO:0070588	BP	calcium ion transmembrane transport	34	4.56E-07
GO:0002831	BP	regulation of response to biotic stimulus	43	4.6E-07
GO:0030858	BP	positive regulation of epithelial cell differentiation	13	4.73E-07
GO:0070664	BP	negative regulation of leukocyte proliferation	17	4.96E-07
GO:0010812	BP	negative regulation of cell-substrate adhesion	13	5.78E-07
GO:0030509	BP	BMP signaling pathway	23	5.85E-07
GO:0050680	BP	negative regulation of epithelial cell proliferation	22	6.08E-07
GO:0003208	BP	cardiac ventricle morphogenesis	15	6.12E-07
GO:0090288	BP	negative regulation of cellular response to growth factor stimulus	17	6.55E-07
GO:0048730	BP	epidermis morphogenesis	11	6.55E-07
GO:0003272	BP	endocardial cushion formation	9	6.67E-07
GO:0031348	BP	negative regulation of defense response	29	6.69E-07
GO:0032886	BP	regulation of microtubule-based process	29	6.69E-07
GO:0045682	BP	regulation of epidermis development	13	7.02E-07
GO:0090303	BP	positive regulation of wound healing	13	7.02E-07
GO:0014855	BP	striated muscle cell proliferation	15	7.18E-07
GO:0043537	BP	negative regulation of blood vessel endothelial cell migration	10	7.32E-07
GO:0006939	BP	smooth muscle contraction	18	7.56E-07
GO:0032970	BP	regulation of actin filament-based process	38	7.64E-07
GO:0055074	BP	calcium ion homeostasis	34	7.8E-07
GO:0050866	BP	negative regulation of cell activation	26	8.49E-07
GO:0032611	BP	interleukin-1 beta production	17	8.6E-07
GO:0048659	BP	smooth muscle cell proliferation	24	9.6E-07
GO:0048486	BP	parasympathetic nervous system development	8	9.64E-07
GO:0022404	BP	molting cycle process	18	9.71E-07
GO:0022405	BP	hair cycle process	18	9.71E-07
GO:1903510	BP	mucopolysaccharide metabolic process	15	9.83E-07
GO:0060038	BP	cardiac muscle cell proliferation	13	1.03E-06
GO:0090497	BP	mesenchymal cell migration	13	1.03E-06
GO:0051098	BP	regulation of binding	38	1.03E-06
GO:0050770	BP	regulation of axonogenesis	23	1.04E-06
GO:2000181	BP	negative regulation of blood vessel morphogenesis	17	1.12E-06
GO:0090090	BP	negative regulation of canonical Wnt signaling pathway	19	1.16E-06

GO:0002065	BP	columnar/cuboidal epithelial cell differentiation	20	1.18E-06
GO:0010596	BP	negative regulation of endothelial cell migration	12	1.22E-06
GO:0048701	BP	embryonic cranial skeleton morphogenesis	12	1.22E-06
GO:0045071	BP	negative regulation of viral genome replication	13	1.23E-06
GO:0010717	BP	regulation of epithelial to mesenchymal transition	16	1.25E-06
GO:1901343	BP	negative regulation of vasculature development	17	1.28E-06
GO:0006929	BP	substrate-dependent cell migration	9	1.29E-06
GO:0002573	BP	myeloid leukocyte differentiation	29	1.32E-06
GO:0048645	BP	animal organ formation	14	1.34E-06
GO:0030501	BP	positive regulation of bone mineralization	11	1.34E-06
GO:0048638	BP	regulation of developmental growth	37	1.42E-06
GO:0002043	BP	blood vessel endothelial cell proliferation involved in sprouting angiogenesis	8	1.43E-06
GO:0051928	BP	positive regulation of calcium ion transport	20	1.47E-06
GO:0032835	BP	glomerulus development	13	1.47E-06
GO:0007611	BP	learning or memory	32	1.57E-06
GO:0051146	BP	striated muscle cell differentiation	33	1.58E-06
GO:0035265	BP	organ growth	25	1.59E-06
GO:0048872	BP	homeostasis of number of cells	36	1.63E-06
GO:0060676	BP	ureteric bud formation	5	1.69E-06
GO:0060561	BP	apoptotic process involved in morphogenesis	9	1.75E-06
GO:0003356	BP	regulation of cilium beat frequency	7	1.76E-06
GO:0007160	BP	cell-matrix adhesion	26	1.78E-06
GO:0008593	BP	regulation of Notch signaling pathway	15	1.79E-06
GO:0010951	BP	negative regulation of endopeptidase activity	23	1.81E-06
GO:0030857	BP	negative regulation of epithelial cell differentiation	12	1.82E-06
GO:0009880	BP	embryonic pattern specification	14	1.84E-06
GO:0061180	BP	mammary gland epithelium development	14	1.84E-06
GO:0007517	BP	muscle organ development	35	1.88E-06
GO:0050921	BP	positive regulation of chemotaxis	20	2.01E-06
GO:0048483	BP	autonomic nervous system development	11	2.09E-06
GO:0072091	BP	regulation of stem cell proliferation	16	2.15E-06
GO:2001237	BP	negative regulation of extrinsic apoptotic signaling pathway	16	2.15E-06
GO:1903036	BP	positive regulation of response to wounding	14	2.16E-06
GO:0003279	BP	cardiac septum development	18	2.24E-06
GO:1903706	BP	regulation of hemopoiesis	39	2.3E-06
GO:0002253	BP	activation of immune response	41	2.42E-06
GO:0006705	BP	mineralocorticoid biosynthetic process	6	2.5E-06
GO:0045607	BP	regulation of inner ear auditory receptor cell differentiation	6	2.5E-06
GO:0045631	BP	regulation of mechanoreceptor differentiation	6	2.5E-06
GO:0060872	BP	semicircular canal development	6	2.5E-06
GO:2000980	BP	regulation of inner ear receptor cell differentiation	6	2.5E-06
GO:0016485	BP	protein processing	29	2.51E-06
GO:0120254	BP	olefinic compound metabolic process	21	2.57E-06
GO:2001236	BP	regulation of extrinsic apoptotic signaling pathway	21	2.57E-06

GO:0060443	BP	mammary gland morphogenesis	12	2.66E-06
GO:0001708	BP	cell fate specification	17	2.69E-06
GO:0002698	BP	negative regulation of immune effector process	19	2.81E-06
GO:0002064	BP	epithelial cell development	26	2.84E-06
GO:0008543	BP	fibroblast growth factor receptor signaling pathway	14	2.93E-06
GO:0035456	BP	response to interferon-beta	13	2.93E-06
GO:0007350	BP	blastoderm segmentation	8	2.94E-06
GO:0090189	BP	regulation of branching involved in ureteric bud morphogenesis	8	2.94E-06
GO:0021953	BP	central nervous system neuron differentiation	24	2.96E-06
GO:0043254	BP	regulation of protein-containing complex assembly	38	3E-06
GO:0045088	BP	regulation of innate immune response	35	3.02E-06
GO:0001942	BP	hair follicle development	17	3.03E-06
GO:0060740	BP	prostate gland epithelium morphogenesis	9	3.13E-06
GO:0061037	BP	negative regulation of cartilage development	9	3.13E-06
GO:0031109	BP	microtubule polymerization or depolymerization	18	3.14E-06
GO:0001974	BP	blood vessel remodeling	12	3.2E-06
GO:0010001	BP	glial cell differentiation	28	3.2E-06
GO:0045089	BP	positive regulation of innate immune response	30	3.38E-06
GO:0007548	BP	sex differentiation	31	3.4E-06
GO:0007585	BP	respiratory gaseous exchange by respiratory system	13	3.45E-06
GO:0035904	BP	aorta development	13	3.45E-06
GO:0030510	BP	regulation of BMP signaling pathway	16	3.59E-06
GO:0048644	BP	muscle organ morphogenesis	15	3.6E-06
GO:0045599	BP	negative regulation of fat cell differentiation	12	3.83E-06
GO:1902074	BP	response to salt	32	3.84E-06
GO:0016331	BP	morphogenesis of embryonic epithelium	22	4.07E-06
GO:0032331	BP	negative regulation of chondrocyte differentiation	8	4.1E-06
GO:0098743	BP	cell aggregation	8	4.1E-06
GO:0021783	BP	preganglionic parasympathetic fiber development	7	4.26E-06
GO:0090660	BP	cerebrospinal fluid circulation	7	4.26E-06
GO:0001755	BP	neural crest cell migration	12	4.56E-06
GO:0010633	BP	negative regulation of epithelial cell migration	13	4.74E-06
GO:0048662	BP	negative regulation of smooth muscle cell proliferation	13	4.74E-06
GO:0031345	BP	negative regulation of cell projection organization	24	4.8E-06
GO:0070507	BP	regulation of microtubule cytoskeleton organization	20	4.88E-06
GO:0097530	BP	granulocyte migration	20	4.88E-06
GO:1902903	BP	regulation of supramolecular fiber organization	35	5.04E-06
GO:0009100	BP	glycoprotein metabolic process	33	5.4E-06
GO:0045778	BP	positive regulation of ossification	12	5.42E-06
GO:2000116	BP	regulation of cysteine-type endopeptidase activity	26	5.55E-06
GO:2000738	BP	positive regulation of stem cell differentiation	8	5.63E-06
GO:0002687	BP	positive regulation of leukocyte migration	20	5.89E-06
GO:0060415	BP	muscle tissue morphogenesis	14	6.01E-06
GO:0010976	BP	positive regulation of neuron projection development	25	6.07E-06

GO:0021700	BP	developmental maturation	34	6.21E-06
GO:0045746	BP	negative regulation of Notch signaling pathway	9	6.88E-06
GO:0019233	BP	sensory perception of pain	19	6.97E-06
GO:0051783	BP	regulation of nuclear division	19	6.97E-06
GO:0002704	BP	negative regulation of leukocyte mediated immunity	13	7.47E-06
GO:0002688	BP	regulation of leukocyte chemotaxis	17	7.5E-06
GO:0003229	BP	ventricular cardiac muscle tissue development	12	7.56E-06
GO:0003231	BP	cardiac ventricle development	19	7.67E-06
GO:0070663	BP	regulation of leukocyte proliferation	28	7.76E-06
GO:0042742	BP	defense response to bacterium	34	8.21E-06
GO:0022408	BP	negative regulation of cell-cell adhesion	23	8.22E-06
GO:0098742	BP	cell-cell adhesion via plasma-membrane adhesion molecules	23	8.22E-06
GO:0007281	BP	germ cell development	36	8.26E-06
GO:0090092	BP	regulation of transmembrane receptor protein serine/threonine kinase signaling pathway	27	8.73E-06
GO:0097191	BP	extrinsic apoptotic signaling pathway	25	8.75E-06
GO:0050772	BP	positive regulation of axonogenesis	15	8.84E-06
GO:0071900	BP	regulation of protein serine/threonine kinase activity	32	8.86E-06
GO:0032731	BP	positive regulation of interleukin-1 beta production	12	8.89E-06
GO:0014015	BP	positive regulation of gliogenesis	14	9.02E-06
GO:1904062	BP	regulation of monoatomic cation transmembrane transport	33	9.05E-06
GO:0090050	BP	positive regulation of cell migration involved in sprouting angiogenesis	7	9.18E-06
GO:0014812	BP	muscle cell migration	17	9.29E-06
GO:0048752	BP	semicircular canal morphogenesis	5	9.5E-06
GO:0048713	BP	regulation of oligodendrocyte differentiation	11	9.95E-06
GO:0061383	BP	trabecula morphogenesis	11	9.95E-06
GO:0070169	BP	positive regulation of biomineral tissue development	11	9.95E-06
GO:0002691	BP	regulation of cellular extravasation	10	1.01E-05
GO:0003016	BP	respiratory system process	10	1.01E-05
GO:0035136	BP	forelimb morphogenesis	10	1.01E-05
GO:2001233	BP	regulation of apoptotic signaling pathway	36	1.02E-05
GO:0045665	BP	negative regulation of neuron differentiation	14	1.03E-05
GO:1903557	BP	positive regulation of tumor necrosis factor superfamily cytokine production	16	1.04E-05
GO:0007380	BP	specification of segmental identity, head	4	1.08E-05
GO:0035287	BP	head segmentation	4	1.08E-05
GO:0035289	BP	posterior head segmentation	4	1.08E-05
GO:0072513	BP	positive regulation of secondary heart field cardioblast proliferation	4	1.08E-05
GO:0048015	BP	phosphatidylinositol-mediated signaling	20	1.11E-05
GO:0031069	BP	hair follicle morphogenesis	9	1.11E-05
GO:0072210	BP	metanephric nephron development	9	1.11E-05
GO:0031532	BP	actin cytoskeleton reorganization	16	1.16E-05
GO:0060041	BP	retina development in camera-type eye	21	1.17E-05
GO:0002820	BP	negative regulation of adaptive immune response	11	1.19E-05
GO:0032941	BP	secretion by tissue	11	1.19E-05

GO:0035270	BP	endocrine system development	18	1.21E-05
GO:0001894	BP	tissue homeostasis	27	1.22E-05
GO:0060249	BP	anatomical structure homeostasis	27	1.22E-05
GO:0030216	BP	keratinocyte differentiation	19	1.22E-05
GO:1902337	BP	regulation of apoptotic process involved in morphogenesis	6	1.23E-05
GO:0071621	BP	granulocyte chemotaxis	17	1.27E-05
GO:0021987	BP	cerebral cortex development	16	1.29E-05
GO:0050768	BP	negative regulation of neurogenesis	20	1.32E-05
GO:0045123	BP	cellular extravasation	13	1.32E-05
GO:0051450	BP	myoblast proliferation	8	1.34E-05
GO:0097202	BP	activation of cysteine-type endopeptidase activity	8	1.34E-05
GO:1903555	BP	regulation of tumor necrosis factor superfamily cytokine production	21	1.38E-05
GO:0003203	BP	endocardial cushion morphogenesis	9	1.4E-05
GO:0007204	BP	positive regulation of cytosolic calcium ion concentration	23	1.4E-05
GO:1990266	BP	neutrophil migration	17	1.41E-05
GO:0035458	BP	cellular response to interferon-beta	11	1.41E-05
GO:0070661	BP	leukocyte proliferation	34	1.41E-05
GO:0048017	BP	inositol lipid-mediated signaling	20	1.44E-05
GO:0031110	BP	regulation of microtubule polymerization or depolymerization	14	1.51E-05
GO:0042490	BP	mechanoreceptor differentiation	14	1.51E-05
GO:0002695	BP	negative regulation of leukocyte activation	22	1.52E-05
GO:0048639	BP	positive regulation of developmental growth	23	1.63E-05
GO:0008347	BP	glial cell migration	12	1.65E-05
GO:0045926	BP	negative regulation of growth	26	1.67E-05
GO:0051896	BP	regulation of protein kinase B signaling	20	1.7E-05
GO:0021602	BP	cranial nerve morphogenesis	8	1.74E-05
GO:0060037	BP	pharyngeal system development	8	1.74E-05
GO:0072331	BP	signal transduction by p53 class mediator	19	1.75E-05
GO:0007589	BP	body fluid secretion	16	1.78E-05
GO:0016525	BP	negative regulation of angiogenesis	15	1.79E-05
GO:0072202	BP	cell differentiation involved in metanephros development	7	1.81E-05
GO:0090190	BP	positive regulation of branching involved in ureteric bud morphogenesis	7	1.81E-05
GO:0050863	BP	regulation of T cell activation	33	1.84E-05
GO:0060513	BP	prostatic bud formation	5	1.84E-05
GO:0006044	BP	N-acetylglucosamine metabolic process	6	1.9E-05
GO:0008212	BP	mineralocorticoid metabolic process	6	1.9E-05
GO:1904748	BP	regulation of apoptotic process involved in development	6	1.9E-05
GO:0001659	BP	temperature homeostasis	21	1.9E-05
GO:0048660	BP	regulation of smooth muscle cell proliferation	21	1.9E-05
GO:0050731	BP	positive regulation of peptidyl-tyrosine phosphorylation	21	1.9E-05
GO:0002764	BP	immune response-regulating signaling pathway	34	1.92E-05
GO:0050672	BP	negative regulation of lymphocyte proliferation	14	1.93E-05
GO:0061053	BP	somite development	14	1.93E-05
GO:0051961	BP	negative regulation of nervous system development	20	2.01E-05

GO:0097553	BP	calcium ion transmembrane import into cytosol	21	2.06E-05
GO:0034765	BP	regulation of monoatomic ion transmembrane transport	39	2.15E-05
GO:0010464	BP	regulation of mesenchymal cell proliferation	9	2.15E-05
GO:0030224	BP	monocyte differentiation	9	2.15E-05
GO:0048588	BP	developmental cell growth	27	2.15E-05
GO:0048806	BP	genitalia development	10	2.17E-05
GO:0032945	BP	negative regulation of mononuclear cell proliferation	14	2.17E-05
GO:0045685	BP	regulation of glial cell differentiation	14	2.17E-05
GO:0010950	BP	positive regulation of endopeptidase activity	20	2.18E-05
GO:0035296	BP	regulation of tube diameter	20	2.18E-05
GO:0097746	BP	blood vessel diameter maintenance	20	2.18E-05
GO:0060317	BP	cardiac epithelial to mesenchymal transition	8	2.25E-05
GO:0043616	BP	keratinocyte proliferation	11	2.3E-05
GO:0035150	BP	regulation of tube size	20	2.37E-05
GO:0042552	BP	myelination	20	2.37E-05
GO:0051100	BP	negative regulation of binding	20	2.37E-05
GO:0071706	BP	tumor necrosis factor superfamily cytokine production	21	2.4E-05
GO:0043586	BP	tongue development	7	2.47E-05
GO:0048485	BP	sympathetic nervous system development	7	2.47E-05
GO:1902547	BP	regulation of cellular response to vascular endothelial growth factor stimulus	7	2.47E-05
GO:0010595	BP	positive regulation of endothelial cell migration	15	2.49E-05
GO:0048525	BP	negative regulation of viral process	15	2.49E-05
GO:0003407	BP	neural retina development	13	2.57E-05
GO:0032412	BP	regulation of monoatomic ion transmembrane transporter activity	27	2.59E-05
GO:0002757	BP	immune response-activating signaling pathway	33	2.64E-05
GO:0038084	BP	vascular endothelial growth factor signaling pathway	9	2.65E-05
GO:0001666	BP	response to hypoxia	23	2.69E-05
GO:0033559	BP	unsaturated fatty acid metabolic process	17	2.78E-05
GO:1902075	BP	cellular response to salt	21	2.8E-05
GO:0003198	BP	epithelial to mesenchymal transition involved in endocardial cushion formation	6	2.83E-05
GO:1903587	BP	regulation of blood vessel endothelial cell proliferation involved in sprouting angiogenesis	6	2.83E-05
GO:2000288	BP	positive regulation of myoblast proliferation	6	2.83E-05
GO:0010574	BP	regulation of vascular endothelial growth factor production	8	2.86E-05
GO:0050670	BP	regulation of lymphocyte proliferation	25	2.98E-05
GO:0007272	BP	ensheathment of neurons	20	3.01E-05
GO:0008366	BP	axon ensheathment	20	3.01E-05
GO:0071466	BP	cellular response to xenobiotic stimulus	20	3.01E-05
GO:0018108	BP	peptidyl-tyrosine phosphorylation	29	3.09E-05
GO:0002823	BP	negative regulation of adaptive immune response based on somatic recombination of immune receptors built from immunoglobulin superfamily domains	10	3.09E-05
GO:0050873	BP	brown fat cell differentiation	11	3.13E-05
GO:0007231	BP	osmosensory signaling pathway	4	3.14E-05
GO:0051090	BP	regulation of DNA-binding transcription factor activity	36	3.15E-05

GO:0003266	BP	regulation of secondary heart field cardioblast proliferation	5	3.26E-05
GO:0032342	BP	aldosterone biosynthetic process	5	3.26E-05
GO:0061162	BP	establishment of monopolar cell polarity	7	3.31E-05
GO:0045109	BP	intermediate filament organization	12	3.34E-05
GO:0032760	BP	positive regulation of tumor necrosis factor production	15	3.43E-05
GO:0018212	BP	peptidyl-tyrosine modification	29	3.45E-05
GO:0032680	BP	regulation of tumor necrosis factor production	20	3.52E-05
GO:0071674	BP	mononuclear cell migration	20	3.52E-05
GO:0048799	BP	animal organ maturation	8	3.62E-05
GO:1901099	BP	negative regulation of signal transduction in absence of ligand	8	3.62E-05
GO:2001240	BP	negative regulation of extrinsic apoptotic signaling pathway in absence of ligand	8	3.62E-05
GO:0021872	BP	forebrain generation of neurons	11	3.64E-05
GO:0010811	BP	positive regulation of cell-substrate adhesion	17	3.67E-05
GO:0016053	BP	organic acid biosynthetic process	28	3.8E-05
GO:0032944	BP	regulation of mononuclear cell proliferation	25	3.84E-05
GO:0022409	BP	positive regulation of cell-cell adhesion	29	3.86E-05
GO:0008207	BP	C21-steroid hormone metabolic process	9	3.92E-05
GO:0060572	BP	morphogenesis of an epithelial bud	6	4.11E-05
GO:0031638	BP	zymogen activation	13	4.2E-05
GO:2001056	BP	positive regulation of cysteine-type endopeptidase activity	18	4.27E-05
GO:0045069	BP	regulation of viral genome replication	14	4.31E-05
GO:0007019	BP	microtubule depolymerization	10	4.32E-05
GO:0007015	BP	actin filament organization	36	4.36E-05
GO:0010952	BP	positive regulation of peptidase activity	21	4.37E-05
GO:0061339	BP	establishment or maintenance of monopolar cell polarity	7	4.38E-05
GO:0070168	BP	negative regulation of biomineral tissue development	7	4.38E-05
GO:0006874	BP	intracellular calcium ion homeostasis	28	4.5E-05
GO:0055001	BP	muscle cell development	23	4.64E-05
GO:0007159	BP	leukocyte cell-cell adhesion	34	4.67E-05
GO:0001569	BP	branching involved in blood vessel morphogenesis	9	4.74E-05
GO:0050918	BP	positive chemotaxis	9	4.74E-05
GO:0002221	BP	pattern recognition receptor signaling pathway	20	4.78E-05
GO:0048546	BP	digestive tract morphogenesis	10	5.08E-05
GO:0030593	BP	neutrophil chemotaxis	14	5.35E-05
GO:0003263	BP	cardioblast proliferation	5	5.42E-05
GO:0003264	BP	regulation of cardioblast proliferation	5	5.42E-05
GO:0032354	BP	response to follicle-stimulating hormone	5	5.42E-05
GO:0060536	BP	cartilage morphogenesis	5	5.42E-05
GO:0070444	BP	oligodendrocyte progenitor proliferation	5	5.42E-05
GO:0070445	BP	regulation of oligodendrocyte progenitor proliferation	5	5.42E-05
GO:0051604	BP	protein maturation	31	5.53E-05
GO:0022898	BP	regulation of transmembrane transporter activity	27	5.55E-05
GO:0046651	BP	lymphocyte proliferation	30	5.6E-05
GO:0046883	BP	regulation of hormone secretion	29	5.63E-05

GO:0035115	BP	embryonic forelimb morphogenesis	8	5.63E-05
GO:0048048	BP	embryonic eye morphogenesis	8	5.63E-05
GO:0003338	BP	metanephros morphogenesis	7	5.72E-05
GO:0090335	BP	regulation of brown fat cell differentiation	7	5.72E-05
GO:0032346	BP	positive regulation of aldosterone metabolic process	3	5.77E-05
GO:0032349	BP	positive regulation of aldosterone biosynthetic process	3	5.77E-05
GO:1902811	BP	positive regulation of skeletal muscle fiber differentiation	3	5.77E-05
GO:0060231	BP	mesenchymal to epithelial transition	6	5.81E-05
GO:0007613	BP	memory	18	5.95E-05
GO:0021545	BP	cranial nerve development	10	5.95E-05
GO:0032640	BP	tumor necrosis factor production	20	5.97E-05
GO:0031334	BP	positive regulation of protein-containing complex assembly	21	6.24E-05
GO:0097193	BP	intrinsic apoptotic signaling pathway	28	6.26E-05
GO:0042130	BP	negative regulation of T cell proliferation	12	6.38E-05
GO:0046887	BP	positive regulation of hormone secretion	20	6.43E-05
GO:0060350	BP	endochondral bone morphogenesis	11	6.44E-05
GO:0043491	BP	protein kinase B signaling	21	6.69E-05
GO:1990138	BP	neuron projection extension	21	6.69E-05
GO:0043114	BP	regulation of vascular permeability	9	6.8E-05
GO:2001239	BP	regulation of extrinsic apoptotic signaling pathway in absence of ligand	9	6.8E-05
GO:0006023	BP	aminoglycan biosynthetic process	10	6.94E-05
GO:0043536	BP	positive regulation of blood vessel endothelial cell migration	10	6.94E-05
GO:0001958	BP	endochondral ossification	8	6.94E-05
GO:0009954	BP	proximal/distal pattern formation	8	6.94E-05
GO:0010573	BP	vascular endothelial growth factor production	8	6.94E-05
GO:0036075	BP	replacement ossification	8	6.94E-05
GO:1902338	BP	negative regulation of apoptotic process involved in morphogenesis	4	7.1E-05
GO:2001016	BP	positive regulation of skeletal muscle cell differentiation	4	7.1E-05
GO:0071560	BP	cellular response to transforming growth factor beta stimulus	23	7.3E-05
GO:1901888	BP	regulation of cell junction assembly	23	7.3E-05
GO:0061437	BP	renal system vasculature development	7	7.38E-05
GO:0061440	BP	kidney vasculature development	7	7.38E-05
GO:0090103	BP	cochlea morphogenesis	7	7.38E-05
GO:0032943	BP	mononuclear cell proliferation	30	7.59E-05
GO:0071675	BP	regulation of mononuclear cell migration	15	7.61E-05
GO:0042098	BP	T cell proliferation	23	7.77E-05
GO:0034763	BP	negative regulation of transmembrane transport	17	8.01E-05
GO:1903038	BP	negative regulation of leukocyte cell-cell adhesion	17	8.01E-05
GO:0072087	BP	renal vesicle development	6	8.03E-05
GO:0002701	BP	negative regulation of production of molecular mediator of immune response	9	8.08E-05
GO:0007156	BP	homophilic cell adhesion via plasma membrane adhesion molecules	14	8.11E-05
GO:0090263	BP	positive regulation of canonical Wnt signaling pathway	14	8.11E-05
GO:0046394	BP	carboxylic acid biosynthetic process	27	8.17E-05
GO:0032956	BP	regulation of actin cytoskeleton organization	30	8.39E-05

GO:0030193	BP	regulation of blood coagulation	11	8.43E-05
GO:0086003	BP	cardiac muscle cell contraction	11	8.43E-05
GO:0120178	BP	steroid hormone biosynthetic process	8	8.5E-05
GO:0001973	BP	G protein-coupled adenosine receptor signaling pathway	5	8.52E-05
GO:0035588	BP	G protein-coupled purinergic receptor signaling pathway	5	8.52E-05
GO:0060601	BP	lateral sprouting from an epithelium	5	8.52E-05
GO:1902548	BP	negative regulation of cellular response to vascular endothelial growth factor stimulus	5	8.52E-05
GO:1903037	BP	regulation of leukocyte cell-cell adhesion	31	8.61E-05
GO:0034101	BP	erythrocyte homeostasis	18	8.84E-05
GO:0042593	BP	glucose homeostasis	26	9.05E-05
GO:0007266	BP	Rho protein signal transduction	16	9.06E-05
GO:0051261	BP	protein depolymerization	15	9.18E-05
GO:0060419	BP	heart growth	15	9.18E-05
GO:0071559	BP	response to transforming growth factor beta	23	9.37E-05
GO:2001234	BP	negative regulation of apoptotic signaling pathway	23	9.37E-05
GO:0051767	BP	nitric-oxide synthase biosynthetic process	7	9.4E-05
GO:0051769	BP	regulation of nitric-oxide synthase biosynthetic process	7	9.4E-05
GO:0060045	BP	positive regulation of cardiac muscle cell proliferation	7	9.4E-05
GO:0060571	BP	morphogenesis of an epithelial fold	7	9.4E-05
GO:0061311	BP	cell surface receptor signaling pathway involved in heart development	7	9.4E-05
GO:0086005	BP	ventricular cardiac muscle cell action potential	7	9.4E-05
GO:0062197	BP	cellular response to chemical stress	29	9.44E-05
GO:0007596	BP	blood coagulation	19	9.45E-05
GO:0033500	BP	carbohydrate homeostasis	26	9.56E-05
GO:0048332	BP	mesoderm morphogenesis	11	9.6E-05
GO:0002832	BP	negative regulation of response to biotic stimulus	16	9.87E-05
GO:0030217	BP	T cell differentiation	28	0.000101
GO:0032413	BP	negative regulation of ion transmembrane transporter activity	12	0.000103
GO:0060411	BP	cardiac septum morphogenesis	12	0.000103
GO:0031668	BP	cellular response to extracellular stimulus	24	0.000103
GO:0021983	BP	pituitary gland development	8	0.000103
GO:0035909	BP	aorta morphogenesis	8	0.000103
GO:0086004	BP	regulation of cardiac muscle cell contraction	8	0.000103
GO:0086091	BP	regulation of heart rate by cardiac conduction	8	0.000103
GO:0002758	BP	innate immune response-activating signaling pathway	20	0.000105
GO:0008016	BP	regulation of heart contraction	20	0.000105
GO:0001502	BP	cartilage condensation	6	0.000109
GO:0006700	BP	C21-steroid hormone biosynthetic process	6	0.000109
GO:2000136	BP	regulation of cell proliferation involved in heart morphogenesis	6	0.000109
GO:1900046	BP	regulation of hemostasis	11	0.000109
GO:0006805	BP	xenobiotic metabolic process	15	0.00011
GO:0035088	BP	establishment or maintenance of apical/basal cell polarity	9	0.000113
GO:0061245	BP	establishment or maintenance of bipolar cell polarity	9	0.000113
GO:0071526	BP	semaphorin-plexin signaling pathway	9	0.000113

GO:0090102	BP	cochlea development	9	0.000113
GO:0006631	BP	fatty acid metabolic process	35	0.000115
GO:0008202	BP	steroid metabolic process	29	0.000115
GO:0002067	BP	glandular epithelial cell differentiation	12	0.000116
GO:0032535	BP	regulation of cellular component size	32	0.000116
GO:0007599	BP	hemostasis	19	0.000117
GO:0050817	BP	coagulation	19	0.000117
GO:0043129	BP	surfactant homeostasis	7	0.000119
GO:0048596	BP	embryonic camera-type eye morphogenesis	7	0.000119
GO:0070977	BP	bone maturation	7	0.000119
GO:0032409	BP	regulation of transporter activity	27	0.000119
GO:0002027	BP	regulation of heart rate	14	0.00012
GO:0051048	BP	negative regulation of secretion	21	0.000122
GO:0030098	BP	lymphocyte differentiation	36	0.000124
GO:2000648	BP	positive regulation of stem cell proliferation	10	0.000124
GO:0099173	BP	postsynapse organization	23	0.000127
GO:0014820	BP	tonic smooth muscle contraction	5	0.000128
GO:0032341	BP	aldosterone metabolic process	5	0.000128
GO:0034309	BP	primary alcohol biosynthetic process	5	0.000128
GO:0072172	BP	mesonephric tubule formation	5	0.000128
GO:1903817	BP	negative regulation of voltage-gated potassium channel activity	5	0.000128
GO:0051250	BP	negative regulation of lymphocyte activation	18	0.000129
GO:0060113	BP	inner ear receptor cell differentiation	12	0.000129
GO:0060043	BP	regulation of cardiac muscle cell proliferation	9	0.000132
GO:0045637	BP	regulation of myeloid cell differentiation	22	0.000137
GO:0032347	BP	regulation of aldosterone biosynthetic process	4	0.000138
GO:0061314	BP	Notch signaling involved in heart development	4	0.000138
GO:1904746	BP	negative regulation of apoptotic process involved in development	4	0.000138
GO:0009855	BP	determination of bilateral symmetry	16	0.000138
GO:0006941	BP	striated muscle contraction	18	0.000139
GO:0050818	BP	regulation of coagulation	11	0.00014
GO:0051770	BP	positive regulation of nitric-oxide synthase biosynthetic process	6	0.000145
GO:0061323	BP	cell proliferation involved in heart morphogenesis	6	0.000145
GO:0072273	BP	metanephric nephron morphogenesis	6	0.000145
GO:2000291	BP	regulation of myoblast proliferation	6	0.000145
GO:0055017	BP	cardiac muscle tissue growth	14	0.000145
GO:0007626	BP	locomotory behavior	24	0.000146
GO:0050730	BP	regulation of peptidyl-tyrosine phosphorylation	24	0.000146
GO:1903522	BP	regulation of blood circulation	24	0.000146
GO:0001558	BP	regulation of cell growth	35	0.000148
GO:0035767	BP	endothelial cell chemotaxis	7	0.000148
GO:0001709	BP	cell fate determination	8	0.00015
GO:0030513	BP	positive regulation of BMP signaling pathway	8	0.00015
GO:0009799	BP	specification of symmetry	16	0.00015

GO:0007264	BP	small GTPase mediated signal transduction	33	0.000151
GO:0050798	BP	activated T cell proliferation	9	0.000154
GO:0050807	BP	regulation of synapse organization	26	0.000155
GO:0001952	BP	regulation of cell-matrix adhesion	15	0.000157
GO:0032271	BP	regulation of protein polymerization	20	0.000158
GO:0021536	BP	diencephalon development	11	0.000158
GO:0061045	BP	negative regulation of wound healing	11	0.000158
GO:0003015	BP	heart process	23	0.00016
GO:0036293	BP	response to decreased oxygen levels	23	0.00016
GO:0050773	BP	regulation of dendrite development	16	0.000163
GO:0048813	BP	dendrite morphogenesis	19	0.000178
GO:0001756	BP	somitogenesis	11	0.000178
GO:0006024	BP	glycosaminoglycan biosynthetic process	9	0.00018
GO:0021879	BP	forebrain neuron differentiation	9	0.00018
GO:0019058	BP	viral life cycle	23	0.00018
GO:0001738	BP	morphogenesis of a polarized epithelium	12	0.00018
GO:0072074	BP	kidney mesenchyme development	5	0.000186
GO:1903365	BP	regulation of fear response	5	0.000186
GO:0030539	BP	male genitalia development	6	0.000189
GO:0031290	BP	retinal ganglion cell axon guidance	6	0.000189
GO:0035089	BP	establishment of apical/basal cell polarity	6	0.000189
GO:0042481	BP	regulation of odontogenesis	6	0.000189
GO:0010634	BP	positive regulation of epithelial cell migration	17	0.00019
GO:0006690	BP	icosanoid metabolic process	16	0.000191
GO:0007088	BP	regulation of mitotic nuclear division	14	0.000191
GO:0007286	BP	spermatid development	24	0.000192
GO:0046777	BP	protein autophosphorylation	22	0.000197
GO:0060047	BP	heart contraction	22	0.000197
GO:0071496	BP	cellular response to external stimulus	27	0.000197
GO:0002088	BP	lens development in camera-type eye	12	0.0002
GO:0034109	BP	homotypic cell-cell adhesion	12	0.0002
GO:0019079	BP	viral genome replication	15	0.000202
GO:0003352	BP	regulation of cilium movement	8	0.000212
GO:0060563	BP	neuroepithelial cell differentiation	8	0.000212
GO:0086002	BP	cardiac muscle cell action potential involved in contraction	8	0.000212
GO:0033326	BP	cerebrospinal fluid secretion	3	0.000224
GO:0042305	BP	specification of segmental identity, mandibular segment	3	0.000224
GO:0072106	BP	regulation of ureteric bud formation	3	0.000224
GO:0072107	BP	positive regulation of ureteric bud formation	3	0.000224
GO:0048146	BP	positive regulation of fibroblast proliferation	11	0.000225
GO:0002053	BP	positive regulation of mesenchymal cell proliferation	7	0.000225
GO:0045616	BP	regulation of keratinocyte differentiation	7	0.000225
GO:0090257	BP	regulation of muscle system process	23	0.000225
GO:0019932	BP	second-messenger-mediated signaling	25	0.000225

GO:0007163	BP	establishment or maintenance of cell polarity	21	0.000228
GO:0051897	BP	positive regulation of protein kinase B signaling	14	0.000229
GO:0090100	BP	positive regulation of transmembrane receptor protein serine/threonine kinase signaling pathway	14	0.000229
GO:0050803	BP	regulation of synapse structure or activity	26	0.000234
GO:0003344	BP	pericardium morphogenesis	4	0.00024
GO:0032344	BP	regulation of aldosterone metabolic process	4	0.00024
GO:1905276	BP	regulation of epithelial tube formation	4	0.00024
GO:1905278	BP	positive regulation of epithelial tube formation	4	0.00024
GO:0043903	BP	regulation of biological process involved in symbiotic interaction	10	0.000241
GO:0002710	BP	negative regulation of T cell mediated immunity	6	0.000244
GO:0019730	BP	antimicrobial humoral response	18	0.000245
GO:0031128	BP	developmental induction	8	0.000251
GO:0001704	BP	formation of primary germ layer	13	0.000253
GO:0002262	BP	myeloid cell homeostasis	20	0.000262
GO:0021984	BP	adenohypophysis development	5	0.000262
GO:0035590	BP	purinergic nucleotide receptor signaling pathway	5	0.000262
GO:0045605	BP	negative regulation of epidermal cell differentiation	5	0.000262
GO:0045683	BP	negative regulation of epidermis development	5	0.000262
GO:0060700	BP	regulation of ribonuclease activity	5	0.000262
GO:0098840	BP	protein transport along microtubule	5	0.000262
GO:0099118	BP	microtubule-based protein transport	5	0.000262
GO:0051258	BP	protein polymerization	24	0.000265
GO:0007498	BP	mesoderm development	14	0.000272
GO:0032410	BP	negative regulation of transporter activity	12	0.000272
GO:1903035	BP	negative regulation of response to wounding	12	0.000272
GO:0001707	BP	mesoderm formation	10	0.000273
GO:0040036	BP	regulation of fibroblast growth factor receptor signaling pathway	7	0.000274
GO:0055010	BP	ventricular cardiac muscle tissue morphogenesis	9	0.000277
GO:0070509	BP	calcium ion import	9	0.000277
GO:1901861	BP	regulation of muscle tissue development	9	0.000277
GO:0140962	BP	multicellular organismal-level chemical homeostasis	11	0.000281
GO:0031669	BP	cellular response to nutrient levels	21	0.000289
GO:1990845	BP	adaptive thermogenesis	17	0.000294
GO:1903532	BP	positive regulation of secretion by cell	30	0.000294
GO:0072330	BP	monocarboxylic acid biosynthetic process	20	0.000297
GO:0035914	BP	skeletal muscle cell differentiation	12	0.000301
GO:0050728	BP	negative regulation of inflammatory response	16	0.000302
GO:0060042	BP	retina morphogenesis in camera-type eye	11	0.000313
GO:0060428	BP	lung epithelium development	9	0.000318
GO:0051402	BP	neuron apoptotic process	27	0.000319
GO:0042129	BP	regulation of T cell proliferation	19	0.00032
GO:1903169	BP	regulation of calcium ion transmembrane transport	19	0.00032
GO:0046620	BP	regulation of organ growth	14	0.000322

GO:0060759	BP	regulation of response to cytokine stimulus	16	0.000325
GO:0048515	BP	spermatid differentiation	24	0.000327
GO:0002063	BP	chondrocyte development	7	0.000331
GO:0045104	BP	intermediate filament cytoskeleton organization	12	0.000332
GO:0009991	BP	response to extracellular stimulus	33	0.000332
GO:0072676	BP	lymphocyte migration	13	0.000333
GO:2000243	BP	positive regulation of reproductive process	13	0.000333
GO:0007179	BP	transforming growth factor beta receptor signaling pathway	19	0.000341
GO:0043405	BP	regulation of MAP kinase activity	19	0.000341
GO:0006040	BP	amino sugar metabolic process	8	0.000345
GO:0032768	BP	regulation of monooxygenase activity	8	0.000345
GO:0019216	BP	regulation of lipid metabolic process	30	0.000349
GO:0008643	BP	carbohydrate transport	16	0.00035
GO:0030218	BP	erythrocyte differentiation	16	0.00035
GO:0060972	BP	left/right pattern formation	15	0.000355
GO:2000696	BP	regulation of epithelial cell differentiation involved in kidney development	5	0.00036
GO:0050922	BP	negative regulation of chemotaxis	9	0.000363
GO:1901617	BP	organic hydroxy compound biosynthetic process	21	0.000363
GO:0045103	BP	intermediate filament-based process	12	0.000365
GO:0045927	BP	positive regulation of growth	26	0.00038
GO:0051259	BP	protein complex oligomerization	22	0.000387
GO:0033007	BP	negative regulation of mast cell activation involved in immune response	4	0.000388
GO:0035581	BP	sequestering of extracellular ligand from receptor	4	0.000388
GO:0048703	BP	embryonic viscerocranium morphogenesis	4	0.000388
GO:1903589	BP	positive regulation of blood vessel endothelial cell proliferation involved in sprouting angiogenesis	4	0.000388
GO:2000342	BP	negative regulation of chemokine (C-X-C motif) ligand 2 production	4	0.000388
GO:1903531	BP	negative regulation of secretion by cell	18	0.00039
GO:0060986	BP	endocrine hormone secretion	10	0.00039
GO:0010575	BP	positive regulation of vascular endothelial growth factor production	6	0.000391
GO:1903975	BP	regulation of glial cell migration	6	0.000391
GO:0015850	BP	organic hydroxy compound transport	25	0.000391
GO:0045833	BP	negative regulation of lipid metabolic process	13	0.000398
GO:0002686	BP	negative regulation of leukocyte migration	8	0.000402
GO:0055023	BP	positive regulation of cardiac muscle tissue growth	8	0.000402
GO:1903115	BP	regulation of actin filament-based movement	8	0.000402
GO:0045824	BP	negative regulation of innate immune response	12	0.000402
GO:0006633	BP	fatty acid biosynthetic process	16	0.000404
GO:0043271	BP	negative regulation of monoatomic ion transport	16	0.000404
GO:0002548	BP	monocyte chemotaxis	9	0.000414
GO:0051445	BP	regulation of meiotic cell cycle	9	0.000414
GO:0051047	BP	positive regulation of secretion	33	0.000421
GO:0009101	BP	glycoprotein biosynthetic process	24	0.000421
GO:0014909	BP	smooth muscle cell migration	13	0.000434

GO:0034767	BP	positive regulation of monoatomic ion transmembrane transport	19	0.000436
GO:0007193	BP	adenylate cyclase-inhibiting G protein-coupled receptor signaling pathway	10	0.000438
GO:0042733	BP	embryonic digit morphogenesis	10	0.000438
GO:0001570	BP	vasculogenesis	12	0.000442
GO:0120193	BP	tight junction organization	12	0.000442
GO:0061844	BP	antimicrobial humoral immune response mediated by antimicrobial peptide	15	0.000446
GO:0010038	BP	response to metal ion	25	0.000452
GO:0045732	BP	positive regulation of protein catabolic process	19	0.000463
GO:0048010	BP	vascular endothelial growth factor receptor signaling pathway	8	0.000466
GO:2001258	BP	negative regulation of cation channel activity	8	0.000466
GO:0060632	BP	regulation of microtubule-based movement	9	0.000471
GO:0014074	BP	response to purine-containing compound	13	0.000472
GO:0061384	BP	heart trabecula morphogenesis	7	0.000473
GO:0034308	BP	primary alcohol metabolic process	11	0.000476
GO:2000736	BP	regulation of stem cell differentiation	11	0.000476
GO:0001959	BP	regulation of cytokine-mediated signaling pathway	15	0.00048
GO:0007189	BP	adenylate cyclase-activating G protein-coupled receptor signaling pathway	15	0.00048
GO:0016045	BP	detection of bacterium	5	0.000483
GO:0033623	BP	regulation of integrin activation	5	0.000483
GO:0034698	BP	response to gonadotropin	5	0.000483
GO:0036159	BP	inner dynein arm assembly	5	0.000483
GO:0046184	BP	aldehyde biosynthetic process	5	0.000483
GO:0072077	BP	renal vesicle morphogenesis	5	0.000483
GO:0061387	BP	regulation of extent of cell growth	14	0.000484
GO:0033622	BP	integrin activation	6	0.000487
GO:0050926	BP	regulation of positive chemotaxis	6	0.000487
GO:0072012	BP	glomerulus vasculature development	6	0.000487
GO:0072677	BP	eosinophil migration	6	0.000487
GO:1901071	BP	glucosamine-containing compound metabolic process	6	0.000487
GO:0055008	BP	cardiac muscle tissue morphogenesis	10	0.00049
GO:0030308	BP	negative regulation of cell growth	19	0.000491
GO:0051091	BP	positive regulation of DNA-binding transcription factor activity	23	0.000499
GO:0050830	BP	defense response to Gram-positive bacterium	17	0.000505
GO:0071248	BP	cellular response to metal ion	17	0.000505
GO:0043523	BP	regulation of neuron apoptotic process	24	0.000512
GO:0034766	BP	negative regulation of monoatomic ion transmembrane transport	13	0.000514
GO:0033674	BP	positive regulation of kinase activity	32	0.000525
GO:0034103	BP	regulation of tissue remodeling	11	0.000526
GO:0042491	BP	inner ear auditory receptor cell differentiation	9	0.000533
GO:0043392	BP	negative regulation of DNA binding	9	0.000533
GO:0061217	BP	regulation of mesonephros development	3	0.000545
GO:0086045	BP	membrane depolarization during AV node cell action potential	3	0.000545
GO:0099183	BP	trans-synaptic signaling by BDNF, modulating synaptic transmission	3	0.000545
GO:1903444	BP	negative regulation of brown fat cell differentiation	3	0.000545

GO:1990926	BP	short-term synaptic potentiation	3	0.000545
GO:0045639	BP	positive regulation of myeloid cell differentiation	13	0.000559
GO:0001953	BP	negative regulation of cell-matrix adhesion	7	0.000561
GO:1904738	BP	vascular associated smooth muscle cell migration	7	0.000561
GO:0048469	BP	cell maturation	20	0.000563
GO:0060048	BP	cardiac muscle contraction	14	0.000565
GO:0050792	BP	regulation of viral process	18	0.000568
GO:1904063	BP	negative regulation of cation transmembrane transport	12	0.000581
GO:0033629	BP	negative regulation of cell adhesion mediated by integrin	4	0.000591
GO:0060056	BP	mammary gland involution	4	0.000591
GO:0060352	BP	cell adhesion molecule production	4	0.000591
GO:0060385	BP	axonogenesis involved in innervation	4	0.000591
GO:0061299	BP	retina vasculature morphogenesis in camera-type eye	4	0.000591
GO:0071372	BP	cellular response to follicle-stimulating hormone stimulus	4	0.000591
GO:0086016	BP	AV node cell action potential	4	0.000591
GO:0086027	BP	AV node cell to bundle of His cell signaling	4	0.000591
GO:1900115	BP	extracellular regulation of signal transduction	4	0.000591
GO:1900116	BP	extracellular negative regulation of signal transduction	4	0.000591
GO:1900147	BP	regulation of Schwann cell migration	4	0.000591
GO:1902260	BP	negative regulation of delayed rectifier potassium channel activity	4	0.000591
GO:0048144	BP	fibroblast proliferation	15	0.000597
GO:0090101	BP	negative regulation of transmembrane receptor protein serine/threonine kinase signaling pathway	15	0.000597
GO:0008209	BP	androgen metabolic process	6	0.000599
GO:0032069	BP	regulation of nuclease activity	6	0.000599
GO:0043931	BP	ossification involved in bone maturation	6	0.000599
GO:0060039	BP	pericardium development	6	0.000599
GO:0060445	BP	branching involved in salivary gland morphogenesis	6	0.000599
GO:0042446	BP	hormone biosynthetic process	9	0.000603
GO:0030902	BP	hindbrain development	17	0.000613
GO:0010837	BP	regulation of keratinocyte proliferation	8	0.000619
GO:0060421	BP	positive regulation of heart growth	8	0.000619
GO:0030502	BP	negative regulation of bone mineralization	5	0.000634
GO:0071639	BP	positive regulation of monocyte chemotactic protein-1 production	5	0.000634
GO:0035051	BP	cardiocyte differentiation	18	0.000641
GO:1903900	BP	regulation of viral life cycle	16	0.000654
GO:0031114	BP	regulation of microtubule depolymerization	7	0.000661
GO:0032964	BP	collagen biosynthetic process	9	0.000679
GO:0120161	BP	regulation of cold-induced thermogenesis	15	0.000688
GO:0031396	BP	regulation of protein ubiquitination	19	0.000696
GO:0002260	BP	lymphocyte homeostasis	11	0.000703
GO:0033627	BP	cell adhesion mediated by integrin	11	0.000703
GO:0042886	BP	amide transport	31	0.000708
GO:0007368	BP	determination of left/right symmetry	14	0.000709

GO:0014829	BP	vascular associated smooth muscle contraction	6	0.000731
GO:0070841	BP	inclusion body assembly	6	0.000731
GO:0060761	BP	negative regulation of response to cytokine stimulus	10	0.000753
GO:0021510	BP	spinal cord development	12	0.000755
GO:0033077	BP	T cell differentiation in thymus	12	0.000755
GO:0001736	BP	establishment of planar polarity	9	0.000764
GO:0002707	BP	negative regulation of lymphocyte mediated immunity	9	0.000764
GO:0010171	BP	body morphogenesis	9	0.000764
GO:0031641	BP	regulation of myelination	9	0.000764
GO:0034340	BP	response to type I interferon	9	0.000764
GO:0060324	BP	face development	9	0.000764
GO:0003158	BP	endothelium development	14	0.000764
GO:0042542	BP	response to hydrogen peroxide	13	0.000772
GO:0032273	BP	positive regulation of protein polymerization	11	0.000773
GO:0032436	BP	positive regulation of proteasomal ubiquitin-dependent protein catabolic process	11	0.000773
GO:0060420	BP	regulation of heart growth	11	0.000773
GO:0030574	BP	collagen catabolic process	7	0.000775
GO:0032633	BP	interleukin-4 production	7	0.000775
GO:0032689	BP	negative regulation of type II interferon production	7	0.000775
GO:0048665	BP	neuron fate specification	7	0.000775
GO:0061436	BP	establishment of skin barrier	7	0.000775
GO:0010718	BP	positive regulation of epithelial to mesenchymal transition	8	0.000811
GO:0031667	BP	response to nutrient levels	30	0.000818
GO:0043116	BP	negative regulation of vascular permeability	5	0.000819
GO:0048557	BP	embryonic digestive tract morphogenesis	5	0.000819
GO:0060973	BP	cell migration involved in heart development	5	0.000819
GO:0001776	BP	leukocyte homeostasis	14	0.000822
GO:0050868	BP	negative regulation of T cell activation	14	0.000822
GO:0051101	BP	regulation of DNA binding	14	0.000822
GO:0046847	BP	filopodium assembly	10	0.000834
GO:1902105	BP	regulation of leukocyte differentiation	27	0.000842
GO:0106106	BP	cold-induced thermogenesis	15	0.000846
GO:0007164	BP	establishment of tissue polarity	9	0.000857
GO:0051785	BP	positive regulation of nuclear division	9	0.000857
GO:0003337	BP	mesenchymal to epithelial transition involved in metanephros morphogenesis	4	0.000859
GO:0014824	BP	artery smooth muscle contraction	4	0.000859
GO:0021561	BP	facial nerve development	4	0.000859
GO:0021610	BP	facial nerve morphogenesis	4	0.000859
GO:0032650	BP	regulation of interleukin-1 alpha production	4	0.000859
GO:0036135	BP	Schwann cell migration	4	0.000859
GO:0060525	BP	prostate glandular acinus development	4	0.000859
GO:0071281	BP	cellular response to iron ion	4	0.000859
GO:0090084	BP	negative regulation of inclusion body assembly	4	0.000859
GO:0090184	BP	positive regulation of kidney development	4	0.000859

GO:1900272	BP	negative regulation of long-term synaptic potentiation	4	0.000859
GO:0008361	BP	regulation of cell size	19	0.00087
GO:0090025	BP	regulation of monocyte chemotaxis	6	0.000884
GO:0035148	BP	tube formation	17	0.000889
GO:0045834	BP	positive regulation of lipid metabolic process	17	0.000889
GO:0003230	BP	cardiac atrium development	7	0.000904
GO:0006636	BP	unsaturated fatty acid biosynthetic process	7	0.000904
GO:0060603	BP	mammary gland duct morphogenesis	7	0.000904
GO:0007632	BP	visual behavior	10	0.000922
GO:0032526	BP	response to retinoic acid	11	0.000928
GO:0070301	BP	cellular response to hydrogen peroxide	11	0.000928
GO:0120192	BP	tight junction assembly	11	0.000928
GO:0001889	BP	liver development	14	0.000949
GO:0014066	BP	regulation of phosphatidylinositol 3-kinase signaling	12	0.000971
GO:0046849	BP	bone remodeling	12	0.000971
GO:0071356	BP	cellular response to tumor necrosis factor	17	0.001002
GO:0051899	BP	membrane depolarization	11	0.001015
GO:0038034	BP	signal transduction in absence of ligand	10	0.001018
GO:0097192	BP	extrinsic apoptotic signaling pathway in absence of ligand	10	0.001018
GO:0003214	BP	cardiac left ventricle morphogenesis	5	0.001041
GO:0003228	BP	atrial cardiac muscle tissue development	5	0.001041
GO:0043117	BP	positive regulation of vascular permeability	5	0.001041
GO:0045652	BP	regulation of megakaryocyte differentiation	5	0.001041
GO:0051797	BP	regulation of hair follicle development	5	0.001041
GO:0090083	BP	regulation of inclusion body assembly	5	0.001041
GO:0097154	BP	GABAergic neuron differentiation	5	0.001041
GO:0098543	BP	detection of other organism	5	0.001041
GO:0035272	BP	exocrine system development	8	0.001047
GO:0060251	BP	regulation of glial cell proliferation	8	0.001047
GO:0031076	BP	embryonic camera-type eye development	7	0.001049
GO:0043551	BP	regulation of phosphatidylinositol 3-kinase activity	7	0.001049
GO:0060325	BP	face morphogenesis	7	0.001049
GO:0110110	BP	positive regulation of animal organ morphogenesis	7	0.001049
GO:0006066	BP	alcohol metabolic process	26	0.001053
GO:0009953	BP	dorsal/ventral pattern formation	12	0.001053
GO:1904427	BP	positive regulation of calcium ion transmembrane transport	12	0.001053
GO:0002826	BP	negative regulation of T-helper 1 type immune response	3	0.001058
GO:0043305	BP	negative regulation of mast cell degranulation	3	0.001058
GO:0048852	BP	diencephalon morphogenesis	3	0.001058
GO:0060129	BP	thyroid-stimulating hormone-secreting cell differentiation	3	0.001058
GO:0070447	BP	positive regulation of oligodendrocyte progenitor proliferation	3	0.001058
GO:0099191	BP	trans-synaptic signaling by BDNF	3	0.001058
GO:1900149	BP	positive regulation of Schwann cell migration	3	0.001058
GO:1904158	BP	axonemal central apparatus assembly	3	0.001058

GO:0030878	BP	thyroid gland development	6	0.001061
GO:0051446	BP	positive regulation of meiotic cell cycle	6	0.001061
GO:0002790	BP	peptide secretion	25	0.001067
GO:0048286	BP	lung alveolus development	9	0.00107
GO:1905517	BP	macrophage migration	9	0.00107
GO:0014065	BP	phosphatidylinositol 3-kinase signaling	14	0.001092
GO:0072332	BP	intrinsic apoptotic signaling pathway by p53 class mediator	11	0.001109
GO:1901879	BP	regulation of protein depolymerization	11	0.001109
GO:1901880	BP	negative regulation of protein depolymerization	10	0.001122
GO:0010563	BP	negative regulation of phosphorus metabolic process	32	0.00114
GO:0045936	BP	negative regulation of phosphate metabolic process	32	0.00114
GO:0070997	BP	neuron death	32	0.00114
GO:0042310	BP	vasoconstriction	12	0.001141
GO:0061008	BP	hepaticobiliary system development	14	0.00117
GO:1901214	BP	regulation of neuron death	29	0.001189
GO:0035315	BP	hair cell differentiation	9	0.001191
GO:0060389	BP	pathway-restricted SMAD protein phosphorylation	9	0.001191
GO:0001547	BP	antral ovarian follicle growth	4	0.001203
GO:0014745	BP	negative regulation of muscle adaptation	4	0.001203
GO:0032610	BP	interleukin-1 alpha production	4	0.001203
GO:0043589	BP	skin morphogenesis	4	0.001203
GO:0048012	BP	hepatocyte growth factor receptor signaling pathway	4	0.001203
GO:0048672	BP	positive regulation of collateral sprouting	4	0.001203
GO:0060272	BP	embryonic skeletal joint morphogenesis	4	0.001203
GO:0086067	BP	AV node cell to bundle of His cell communication	4	0.001203
GO:0140059	BP	dendrite arborization	4	0.001203
GO:0034612	BP	response to tumor necrosis factor	18	0.001206
GO:0010803	BP	regulation of tumor necrosis factor-mediated signaling pathway	7	0.001212
GO:0045761	BP	regulation of adenylate cyclase activity	7	0.001212
GO:0086065	BP	cell communication involved in cardiac conduction	7	0.001212
GO:0048145	BP	regulation of fibroblast proliferation	13	0.001218
GO:0007292	BP	female gamete generation	16	0.001234
GO:1904064	BP	positive regulation of cation transmembrane transport	16	0.001234
GO:0051145	BP	smooth muscle cell differentiation	10	0.001234
GO:1903322	BP	positive regulation of protein modification by small protein conjugation or removal	14	0.001253
GO:0001945	BP	lymph vessel development	6	0.001263
GO:0030204	BP	chondroitin sulfate metabolic process	6	0.001263
GO:0034505	BP	tooth mineralization	6	0.001263
GO:0035455	BP	response to interferon-alpha	6	0.001263
GO:0043552	BP	positive regulation of phosphatidylinositol 3-kinase activity	6	0.001263
GO:0060384	BP	innervation	6	0.001263
GO:0060977	BP	coronary vasculature morphogenesis	6	0.001263
GO:1990806	BP	ligand-gated ion channel signaling pathway	6	0.001263
GO:0044403	BP	biological process involved in symbiotic interaction	21	0.001271

GO:0071692	BP	protein localization to extracellular region	31	0.001276
GO:0034764	BP	positive regulation of transmembrane transport	22	0.001285
GO:0045606	BP	positive regulation of epidermal cell differentiation	5	0.001305
GO:0046629	BP	gamma-delta T cell activation	5	0.001305
GO:0060438	BP	trachea development	5	0.001305
GO:1900424	BP	regulation of defense response to bacterium	5	0.001305
GO:1900746	BP	regulation of vascular endothelial growth factor signaling pathway	5	0.001305
GO:1901890	BP	positive regulation of cell junction assembly	13	0.00131
GO:0009798	BP	axis specification	11	0.001317
GO:0006979	BP	response to oxidative stress	29	0.00133
GO:0043393	BP	regulation of protein binding	19	0.001333
GO:0030522	BP	intracellular receptor signaling pathway	21	0.001333
GO:0007200	BP	phospholipase C-activating G protein-coupled receptor signaling pathway	12	0.001336
GO:0032642	BP	regulation of chemokine production	12	0.001336
GO:0062014	BP	negative regulation of small molecule metabolic process	12	0.001336
GO:0032088	BP	negative regulation of NF-kappaB transcription factor activity	10	0.001356
GO:0071479	BP	cellular response to ionizing radiation	10	0.001356
GO:0016032	BP	viral process	25	0.001373
GO:0044331	BP	cell-cell adhesion mediated by cadherin	7	0.001393
GO:0045747	BP	positive regulation of Notch signaling pathway	7	0.001393
GO:0046006	BP	regulation of activated T cell proliferation	7	0.001393
GO:0085029	BP	extracellular matrix assembly	7	0.001393
GO:0043087	BP	regulation of GTPase activity	26	0.0014
GO:0030048	BP	actin filament-based movement	13	0.001407
GO:0062023	CC	collagen-containing extracellular matrix	59	4.12E-19
GO:0005604	CC	basement membrane	26	2.85E-13
GO:0005930	CC	axoneme	27	6.64E-12
GO:0097014	CC	ciliary plasm	27	7.9E-12
GO:0031514	CC	motile cilium	38	1.63E-11
GO:0032838	CC	plasma membrane bounded cell projection cytoplasm	28	1.79E-09
GO:0097729	CC	9+2 motile cilium	27	1.69E-08
GO:0045178	CC	basal part of cell	35	3.38E-08
GO:0009925	CC	basal plasma membrane	33	5.11E-08
GO:0099568	CC	cytoplasmic region	28	1.88E-07
GO:1990716	CC	axonemal central apparatus	5	5.22E-07
GO:0014069	CC	postsynaptic density	40	8.59E-07
GO:0005874	CC	microtubule	39	2.06E-06
GO:0042383	CC	sarcolemma	21	2.22E-06
GO:0005912	CC	adherens junction	20	2.3E-06
GO:0032279	CC	asymmetric synapse	40	2.59E-06
GO:0036126	CC	sperm flagellum	22	2.76E-06
GO:0005581	CC	collagen trimer	14	2.8E-06
GO:0005875	CC	microtubule associated complex	19	5.36E-06
GO:0005858	CC	axonemal dynein complex	8	6.02E-06

GO:0015629	CC	actin cytoskeleton	41	7.4E-06
GO:0019897	CC	extrinsic component of plasma membrane	21	8.07E-06
GO:0099572	CC	postsynaptic specialization	40	8.73E-06
GO:0016324	CC	apical plasma membrane	35	1.18E-05
GO:0045121	CC	membrane raft	34	1.23E-05
GO:0098857	CC	membrane microdomain	34	1.3E-05
GO:0030286	CC	dynein complex	11	1.81E-05
GO:0043235	CC	receptor complex	35	2.29E-05
GO:0043204	CC	perikaryon	18	3.41E-05
GO:0005583	CC	fibrillar collagen trimer	5	5.66E-05
GO:0036156	CC	inner dynein arm	5	5.66E-05
GO:0098643	CC	banded collagen fibril	5	5.66E-05
GO:0097169	CC	AIM2 inflammasome complex	3	5.94E-05
GO:0016323	CC	basolateral plasma membrane	24	8.31E-05
GO:0045211	CC	postsynaptic membrane	29	0.000116
GO:0000940	CC	outer kinetochore	5	0.000274
GO:0072687	CC	meiotic spindle	5	0.000274
GO:0031234	CC	extrinsic component of cytoplasmic side of plasma membrane	12	0.000327
GO:0030057	CC	desmosome	6	0.000327
GO:0019898	CC	extrinsic component of membrane	26	0.000397
GO:0002177	CC	manchette	5	0.000661
GO:0098644	CC	complex of collagen trimers	5	0.000661
GO:0005201	MF	extracellular matrix structural constituent	28	3.94E-12
GO:0005539	MF	glycosaminoglycan binding	33	2.21E-10
GO:0005125	MF	cytokine activity	29	3.51E-08
GO:0050840	MF	extracellular matrix binding	14	3.72E-08
GO:0008201	MF	heparin binding	24	8.79E-08
GO:0050839	MF	cell adhesion molecule binding	33	1.53E-07
GO:0019838	MF	growth factor binding	21	2.71E-07
GO:0008569	MF	minus-end-directed microtubule motor activity	8	4.46E-07
GO:0005516	MF	calmodulin binding	24	5.62E-07
GO:0043394	MF	proteoglycan binding	11	5.73E-07
GO:0004857	MF	enzyme inhibitor activity	37	8.58E-07
GO:0008017	MF	microtubule binding	29	9.18E-07
GO:0015631	MF	tubulin binding	36	1.79E-06
GO:0001664	MF	G protein-coupled receptor binding	32	2.3E-06
GO:0005126	MF	cytokine receptor binding	31	2.94E-06
GO:0003779	MF	actin binding	39	3.05E-06
GO:0004867	MF	serine-type endopeptidase inhibitor activity	17	4E-06
GO:0004175	MF	endopeptidase activity	39	4.9E-06
GO:1901681	MF	sulfur compound binding	29	5.1E-06
GO:0003777	MF	microtubule motor activity	13	5.39E-06
GO:0008081	MF	phosphoric diester hydrolase activity	14	6E-06
GO:0019199	MF	transmembrane receptor protein kinase activity	13	6.29E-06

GO:0005178	MF	integrin binding	19	9.99E-06
GO:0051959	MF	dynein light intermediate chain binding	8	1.11E-05
GO:0061134	MF	peptidase regulator activity	27	1.32E-05
GO:0030414	MF	peptidase inhibitor activity	23	1.57E-05
GO:0004713	MF	protein tyrosine kinase activity	17	1.64E-05
GO:0045505	MF	dynein intermediate chain binding	9	1.91E-05
GO:0008009	MF	chemokine activity	9	2.91E-05
GO:0099106	MF	ion channel regulator activity	17	2.94E-05
GO:0070851	MF	growth factor receptor binding	18	3.24E-05
GO:0001517	MF	N-acetylglucosamine 6-O-sulfotransferase activity	4	3.29E-05
GO:0001730	MF	2'-5'-oligoadenylate synthetase activity	4	3.29E-05
GO:0042578	MF	phosphoric ester hydrolase activity	30	4.56E-05
GO:0004866	MF	endopeptidase inhibitor activity	21	5.98E-05
GO:0004117	MF	calmodulin-activated dual specificity 3',5'-cyclic-GMP, 3',5'-cyclic-AMP phosphodiesterase activity	3	5.99E-05
GO:0048101	MF	calmodulin-activated 3',5'-cyclic-GMP phosphodiesterase activity	3	5.99E-05
GO:0061135	MF	endopeptidase regulator activity	22	6.22E-05
GO:0016247	MF	channel regulator activity	17	6.61E-05
GO:0106256	MF	hydroperoxy icosatetraenoate dehydratase activity	4	7.44E-05
GO:0004222	MF	metalloendopeptidase activity	15	8.69E-05
GO:0004714	MF	transmembrane receptor protein tyrosine kinase activity	10	8.91E-05
GO:0031994	MF	insulin-like growth factor I binding	5	9.02E-05
GO:0030020	MF	extracellular matrix structural constituent conferring tensile strength	8	9.24E-05
GO:0005506	MF	iron ion binding	18	0.000129
GO:0001227	MF	DNA-binding transcription repressor activity, RNA polymerase II-specific	27	0.00013
GO:0042562	MF	hormone binding	12	0.000145
GO:0140103	MF	catalytic activity, acting on a glycoprotein	6	0.000155
GO:0001217	MF	DNA-binding transcription repressor activity	27	0.00016
GO:0051015	MF	actin filament binding	21	0.000174
GO:0005044	MF	scavenger receptor activity	8	0.00023
GO:0003774	MF	cytoskeletal motor activity	14	0.000237
GO:0008376	MF	acetylgalactosaminyltransferase activity	7	0.000242
GO:0045545	MF	syndecan binding	4	0.000251
GO:0043395	MF	heparan sulfate proteoglycan binding	6	0.000261
GO:0061629	MF	RNA polymerase II-specific DNA-binding transcription factor binding	30	0.000264
GO:0005112	MF	Notch binding	6	0.000332
GO:0004497	MF	monooxygenase activity	16	0.000345
GO:0038024	MF	cargo receptor activity	11	0.000347
GO:0030547	MF	signaling receptor inhibitor activity	7	0.000356
GO:0008237	MF	metallopeptidase activity	19	0.000371

Table S2 KEGG analysis of DEGs

ID	Term	Count	Gene ID	pvalue
mmu04820	Cytoskeleton in muscle cells - Mus musculus (house mouse)	31	Dsc2/Itgb4/Lama1/Itga6/Tnni2/Dcn/Myh10/Xirp2/Tmod1/Col5a3/Myl9/Col3a1/Thbs2/Fmnl1/Flnc/Fbn2/Des/Nid2/Col6a2/Atp1a3/Vim/Myom2/Ank2/Fbn1/Col5a1/Trim54/Col1a2/Bgn/Mybph/Mybpc2/Tnni3	8.9E-07
mmu04020	Calcium signaling pathway - Mus musculus (house mouse)	31	Fgfr3/Met/Mst1r/Mst1/Gdnf/Cacna1c/Grin1/Gna15/Ntrk2/Cysltr1/Pdgfra/Plcb1/Adcy2/Mylk/Atp2b4/Plcd4/Cckar/Cacna1h/Fgf2/Smim6/Fgf7/Mcoln3/Adcy1/Vegfc/Pde1c/Cacna1g/Pde1a/Pde1b/Htr2a/Chrm3/Ednra	8.08E-06
mmu04924	Renin secretion - Mus musculus (house mouse)	12	Cacna1c/Clca3b/Plcb1/Npr1/Adora1/Pde1c/Pde1a/Ptger4/Pde1b/Ednra/Pde3a/Edn3	0.000516
mmu04350	TGF-beta signaling pathway - Mus musculus (house mouse)	15	Bmp7/Id4/Bmpr1b/Fst/Bmp5/Dcn/Bmp6/Fmod/Trf/Nog/Chrd/Grem1/Fbn1/Grem2/Amhr2	0.000555
mmu00512	Mucin type O-glycan biosynthesis - Mus musculus (house mouse)	7	B3gnt3/Gcnt1/Galnt18/Galnt12/Galnt6/St6galnac4/Galnt17	0.001079
mmu04062	Chemokine signaling pathway - Mus musculus (house mouse)	21	Cxcl5/Tiam1/Vav3/Jak3/Gnb4/Lyn/Ccl2/Ccl7/Ppbp/Pik3r5/Plcb1/Adcy2/Hck/Shc4/Adcy1/Gng11/Cxcl12/Ccl11/Ccr1/Ccl20/Cxcl3	0.001086
mmu00230	Purine metabolism - Mus musculus (house mouse)	16	Enpp1/Entpd3/Adssl1/Urah/Enpp3/Ak7/Adcy2/Npr1/Adcy1/Pde1c/Entpd1/Pde1a/Ak9/Pde10a/Pde1b/Pde3a	0.001587
mmu00980	Metabolism of xenobiotics by cytochrome P450 - Mus musculus (house mouse)	11	Cyp2f2/Cbr2/Gsta4/Cyp2s1/Gsto1/Aldh3a1/Cyp1b1/Adh7/Mgst2/Ugt1a6a/Gstm7	0.00182
mmu04611	Platelet activation - Mus musculus (house mouse)	15	P2ry1/Lyn/Fyn/Pik3r5/Plcb1/Adcy2/Mylk/Apbb1ip/Col3a1/Adcy1/Prkg1/Tbxas1/P2ry12/Col1a2/Ptgir	0.002091
mmu05032	Morphine addiction - Mus musculus (house mouse)	12	Gabrp/Gnb4/Adcy2/Adcy1/Adora1/Pde1c/Gabre/Pde1a/Pde10a/Pde1b/Gng11/Pde3a	0.002837
mmu04060	Cytokine-cytokine receptor interaction - Mus musculus (house mouse)	27	Il33/Cxcl5/Tnfrsf21/Bmp7/Ackr3/Ngfr/Cxcl17/Bmpr1b/Bmp5/Bmp6/Ccl2/Ccl7/Il1rl2/Csf3/Ppbp/Tnfsf10/Il1a/Tnfsf12/Il13ra2/Thpo/Tnfrsf18/Cxcl12/Ccl11/Ccr1/Amhr2/Ccl20/Cxcl3	0.002935
mmu04623	Cytosolic DNA-sensing pathway - Mus musculus (house mouse)	11	Il33/Sting1/Pycard/Gsdmd/Ripk3/Casp1/Zbp1/Aim2/Cgas/Ifi204/Ifi202b	0.003361
mmu04814	Motor proteins - Mus musculus (house mouse)	20	Dnah10/Kif1a/Kif19a/Dnah5/Tnni2/Dnah6/Dnah3/Cenpe/Dnah2/Tubb2b/Dnah1/Myh10/Dnah9/Kif2c/Myl9/Stard9/Dnali1/Dnah11/Dynlrb2/Tnni3	0.003418
mmu04974	Protein digestion and absorption - Mus musculus (house mouse)	13	Col17a1/Col7a1/Col25a1/Kcne3/Col15a1/Slc7a8/Col5a3/Col3a1/Col6a2/Atp1a3/Col5a1/Col1a2/Col26a1	0.003939
mmu04360	Axon guidance - Mus musculus (house mouse)	18	Wnt4/Ephb2/Sema3f/Met/Bmp7/Boc/Bmpr1b/Efna5/Unc5a/Sema3d/Sema6b/Fyn/Nrp1/Sema4f/Myl9/Wnt5b/Lrrc4/Cxcl12	0.006268
mmu04024	cAMP signaling pathway - Mus musculus (house mouse)	21	Tiam1/Vav3/Cacna1c/Bdnf/Grin1/Adcy2/Atp2b4/Hcar2/Myl9/Vipr2/Npr1/Adcy1/Adora1/Ppp1r1b/Sstr2/Pde10a/Atp1a3/Ednra/Pde3a/Edn3/Tnni3	0.006516
mmu04657	IL-17 signaling pathway - Mus musculus (house mouse)	11	Cxcl5/Muc5ac/Ccl2/Ccl7/Csf3/Mapk15/Mmp3/Mapk4/Ccl11/Ccl20/Cxcl3	0.008765
mmu04510	Focal adhesion - Mus musculus (house mouse)	19	Itgb4/Lama1/Cav1/Itga6/Vav3/Met/Fyn/Pdgfra/Mylk/Parvb/Myl9/Shc4/Thbs2/Vegfc/Spp1/Col6a2/Rasgrf1/Col1a2/Parvg	0.00908
mmu00910	Nitrogen metabolism - Mus musculus (house mouse)	4	Car5b/Glul/Car9/Car12	0.010079

mmu04061	Viral protein interaction with cytokine and cytokine receptor - Mus musculus (house mouse)	11	Cxcl5/Ackr3/Ccl2/Ccl7/Ppbp/Tnfsf10/Cxcl12/Ccl11/Ccr1/Ccl20/Cxcl3	0.010232
mmu00982	Drug metabolism - cytochrome P450 - Mus musculus (house mouse)	9	Fmo2/Gsta4/Gsto1/Aldh3a1/Adh7/Fmo3/Mgst2/Ugt1a6a/Gstm7	0.012052
mmu05202	Transcriptional misregulation in cancer - Mus musculus (house mouse)	20	Six1/Prom1/Met/Eya1/Ngfr/Cd14/Slc45a3/Igfbp3/Ccna2/Bcl11b/Plat/Pax3/Zeb1/Mycn/Mmp3/Runx1t1/Arnt2/Fli1/Mef2c/Mlf1	0.014236
mmu04550	Signaling pathways regulating pluripotency of stem cells - Mus musculus (house mouse)	14	Wnt4/Sox2/Otx1/Fgfr3/Jak3/Id4/Bmpr1b/Klf4/Dusp9/Tbx3/Fgf2/Wnt5b/Apc2/Hoxa1	0.014256
mmu04970	Salivary secretion - Mus musculus (house mouse)	10	Aqp5/Muc5ac/Trpv6/Plcb1/Adcy2/Atp2b4/Adcy1/Prkg1/Chrm3/Atp1a3	0.014705
mmu04115	p53 signaling pathway - Mus musculus (house mouse)	9	Serpinb5/Perp/Igfbp3/Ccnb2/Ccnb1/Trp73/Cdkn2a/Steap3/Rprm	0.015513
mmu04151	PI3K-Akt signaling pathway - Mus musculus (house mouse)	29	Itgb4/Lama1/Itga6/Fgfr3/Met/Ngfr/Jak3/Efna5/Gnb4/Gdnf/Sgk3/Bdnf/Myb/Ntrk2/Csf3/Pdgfra/Pik3r5/Artn/Fgf2/Thbs2/Fgf7/Vegfc/Spp1/Lpar3/Angpt2/Col6a2/Gng11/Col1a2/Ppp2r2c	0.016328
mmu04512	ECM-receptor interaction - Mus musculus (house mouse)	10	Itgb4/Lama1/Itga6/Fras1/Thbs2/Dmp1/Spp1/Col6a2/Col1a2/Sv2c	0.017051
mmu04022	cGMP-PKG signaling pathway - Mus musculus (house mouse)	16	Cacna1c/Pik3r5/Plcb1/Adcy2/Mylk/Atp2b4/Myl9/Npr1/Adcy1/Adora1/Prkg1/Atp1a3/Ednra/Pde3a/Mef2c/Gata4	0.01746
mmu04270	Vascular smooth muscle contraction - Mus musculus (house mouse)	14	Adm/Prkch/Cacna1c/Myh10/Plcb1/Adcy2/Mylk/Myl9/Npr1/Adcy1/Prkg1/Ednra/Ptgir/Edn3	0.017866
mmu00532	Glycosaminoglycan biosynthesis - chondroitin sulfate / dermatan sulfate - Mus musculus (house mouse)	4	Chst11/Chst7/Chst3/Csgalnact1	0.021503
mmu00770	Pantothenate and CoA biosynthesis - Mus musculus (house mouse)	4	Enpp1/Enpp3/Bcat1/Vnn3	0.021503
mmu04014	Ras signaling pathway - Mus musculus (house mouse)	20	Tiam1/Fgfr3/Met/Ngfr/Efna5/Gnb4/Bdnf/Grin1/Ntrk2/Pdgfra/Shc4/Rasa4/Fgf2/Fgf7/Vegfc/Angpt2/Rasgrf2/Gng11/Rasgrf1/Pla1a	0.021783
mmu04010	MAPK signaling pathway - Mus musculus (house mouse)	24	Fgfr3/Met/Ngfr/Cd14/Hspa1b/Efna5/Hspa1a/Gdnf/Cacna1c/Bdnf/Ntrk2/Pdgfra/Cacna1h/Dusp9/Artn/Fgf2/Il1a/Fgf7/Vegfc/Cacna1g/Angpt2/Rasgrf2/Mef2c/Rasgrf1	0.023159
mmu04514	Cell adhesion molecules - Mus musculus (house mouse)	16	Cdh3/Itga6/Vtcn1/Vsir/Cldn2/Cldn23/Cldn10/Cldn8/Ptprd/Esam/Cdh2/Lrrc4/Nrcam/Jam3/Icosl/Spn	0.024444
mmu05146	Amoebiasis - Mus musculus (house mouse)	11	Lama1/Serpinb10/Cd14/Serpinb9b/Arg1/Gna15/Plcb1/Col3a1/Adcy1/Col1a2/Cxcl3	0.024787
mmu00430	Taurine and hypotaurine metabolism - Mus musculus (house mouse)	4	Fmo2/Cdo1/Fmo3/Ggt5	0.025228
mmu04015	Rap1 signaling pathway - Mus musculus (house mouse)	18	Tiam1/Vav3/Fgfr3/Met/P2ry1/Ngfr/Efna5/Grin1/Pdgfra/Plcb1/Adcy2/Apbb1ip/Fgf2/Fgf7/Adcy1/Vegfc/Lpar3/Angpt2	0.030456
mmu04621	NOD-like receptor signaling pathway - Mus musculus (house mouse)	18	Sting1/Txnip/Ccl2/Pycard/Gsdmd/Ripk3/Plcb1/Oas2/Casp1/Naip5/Nod2/Aim2/Oas3/Ifi204/Oas1a/Trpv2/Cxcl3/Oas1g	0.032981
mmu04713	Circadian entrainment - Mus musculus (house mouse)	10	Gnb4/Cacna1c/Grin1/Plcb1/Adcy2/Cacna1h/Adcy1/Cacna1g/Prkg1/Gng11	0.033074

mmu04911	Insulin secretion - Mus musculus (house mouse)	9	Cacna1c/Plcb1/Adcy2/Cckar/Adcy1/Chrm3/Atp1a3/Ffar1/Abcc8	0.034508
mmu05134	Legionellosis - Mus musculus (house mouse)	7	Cd14/Hspa1b/Hspa1a/Pycard/Casp1/Naip5/Cxcl3	0.035504
mmu04390	Hippo signaling pathway - Mus musculus (house mouse)	14	Wnt4/Sox2/Bmp7/Bmpr1b/Bmp5/Snai2/Bmp6/Trp73/Wnt5b/Apc2/Lef1/Dlg2/Gli2/Ppp2r2c	0.036096
mmu05323	Rheumatoid arthritis - Mus musculus (house mouse)	9	Cxcl5/Ccl2/Acp5/Il1a/Mmp3/Cxcl12/Ccl20/Cxcl3/Atp6v1g3	0.036793
mmu04930	Type II diabetes mellitus - Mus musculus (house mouse)	6	Cacna1c/Hkdc1/Cacna1g/Abcc8/Mafa/Slc2a4	0.037287
mmu00590	Arachidonic acid metabolism - Mus musculus (house mouse)	9	Cbr2/Alox12/Ptges/Alox15/Tbxas1/Cyp2c55/Ggt5/Ptgis/Cyp2j9	0.039179
mmu04310	Wnt signaling pathway - Mus musculus (house mouse)	15	Wnt4/Lgr6/Porcn/Lgr4/Dkk2/Ccn4/Plcb1/Serpinf1/Wnt5b/Apc2/Ctnnd2/Ror2/Lef1/Prickle2/Frat1	0.039578
mmu04925	Aldosterone synthesis and secretion - Mus musculus (house mouse)	10	Cacna1c/Plcb1/Adcy2/Atp2b4/Cacna1h/Cyp21a1/Npr1/Adcy1/Cacna1g/Atp1a3	0.041753
mmu05224	Breast cancer - Mus musculus (house mouse)	13	Wnt4/Jag2/Hey1/Notch1/Shc4/Fgf2/Wnt5b/Fgf7/Apc2/Lef1/Frat1/Esr1/Dll4	0.042956
mmu04934	Cushing syndrome - Mus musculus (house mouse)	14	Wnt4/Ahr/Cacna1c/Plcb1/Adcy2/Cacna1h/Cyp21a1/Wnt5b/Adcy1/Apc2/Cacna1g/Lef1/Cdkn2a/Aipl1	0.043157
mmu04915	Estrogen signaling pathway - Mus musculus (house mouse)	12	Krt17/Krt14/Krt15/Hspa1b/Krt20/Hspa1a/Plcb1/Krt13/Adcy2/Shc4/Adcy1/Esr1	0.046637
mmu00601	Glycosphingolipid biosynthesis - lacto and neolacto series - Mus musculus (house mouse)	4	B3gnt3/B3gnt5/Fut2/B3galt1	0.049399

Table S3 GO analysis of potential therapeutic targets

ID	Category	Term	Count	pvalue
GO:0003018	BP	vascular process in circulatory system	15	8.67E-18
GO:0006874	BP	intracellular calcium ion homeostasis	14	1.42E-14
GO:0150063	BP	visual system development	15	4.67E-14
GO:0055074	BP	calcium ion homeostasis	14	5.14E-14
GO:0048880	BP	sensory system development	15	5.34E-14
GO:0043270	BP	positive regulation of monoatomic ion transport	13	5.69E-14
GO:0003012	BP	muscle system process	15	5.91E-14
GO:2000241	BP	regulation of reproductive process	12	1.44E-13
GO:0001667	BP	ameboidal-type cell migration	15	1.75E-13
GO:0042060	BP	wound healing	14	2.7E-13
GO:0035296	BP	regulation of tube diameter	11	3.42E-13
GO:0097746	BP	blood vessel diameter maintenance	11	3.42E-13
GO:0035150	BP	regulation of tube size	11	3.64E-13
GO:0070372	BP	regulation of ERK1 and ERK2 cascade	13	5.86E-13
GO:0001654	BP	eye development	14	8.33E-13
GO:0070588	BP	calcium ion transmembrane transport	13	8.56E-13

GO:0070371	BP	ERK1 and ERK2 cascade	13	1.43E-12
GO:0006816	BP	calcium ion transport	14	1.85E-12
GO:0070374	BP	positive regulation of ERK1 and ERK2 cascade	11	4.81E-12
GO:0061138	BP	morphogenesis of a branching epithelium	11	5.04E-12
GO:0006936	BP	muscle contraction	12	8.48E-12
GO:0001763	BP	morphogenesis of a branching structure	11	1.25E-11
GO:0097553	BP	calcium ion transmembrane import into cytosol	10	2.38E-11
GO:0033002	BP	muscle cell proliferation	11	3.35E-11
GO:0043010	BP	camera-type eye development	12	6.28E-11
GO:0019216	BP	regulation of lipid metabolic process	12	9.82E-11
GO:1901342	BP	regulation of vasculature development	11	1.26E-10
GO:0072006	BP	nephron development	9	1.36E-10
GO:0030336	BP	negative regulation of cell migration	11	1.39E-10
GO:2000146	BP	negative regulation of cell motility	11	2.15E-10
GO:0050678	BP	regulation of epithelial cell proliferation	12	2.54E-10
GO:1903034	BP	regulation of response to wounding	9	3E-10
GO:0001822	BP	kidney development	11	3.06E-10
GO:0006939	BP	smooth muscle contraction	8	3.36E-10
GO:1905330	BP	regulation of morphogenesis of an epithelium	7	3.66E-10
GO:0086004	BP	regulation of cardiac muscle cell contraction	6	3.7E-10
GO:0090257	BP	regulation of muscle system process	10	4.6E-10
GO:0072001	BP	renal system development	11	5E-10
GO:0048754	BP	branching morphogenesis of an epithelial tube	9	5.89E-10
GO:0010959	BP	regulation of metal ion transport	12	5.92E-10
GO:0001659	BP	temperature homeostasis	9	6.17E-10
GO:0040013	BP	negative regulation of locomotion	11	6.33E-10
GO:1903522	BP	regulation of blood circulation	10	6.39E-10
GO:0061041	BP	regulation of wound healing	8	1.04E-09
GO:1903115	BP	regulation of actin filament-based movement	6	1.2E-09
GO:0006942	BP	regulation of striated muscle contraction	7	1.28E-09
GO:0045765	BP	regulation of angiogenesis	10	2.07E-09
GO:0019233	BP	sensory perception of pain	8	2.36E-09
GO:0072073	BP	kidney epithelium development	8	2.76E-09
GO:0043542	BP	endothelial cell migration	9	3.02E-09
GO:0090596	BP	sensory organ morphogenesis	10	3.18E-09
GO:0048864	BP	stem cell development	7	3.19E-09
GO:0048545	BP	response to steroid hormone	9	3.79E-09
GO:1901617	BP	organic hydroxy compound biosynthetic process	9	3.79E-09
GO:0010631	BP	epithelial cell migration	10	4.03E-09
GO:0090132	BP	epithelium migration	10	4.27E-09
GO:0090130	BP	tissue migration	10	4.53E-09
GO:0006937	BP	regulation of muscle contraction	8	5.2E-09
GO:0048762	BP	mesenchymal cell differentiation	9	6.04E-09
GO:0006066	BP	alcohol metabolic process	10	6.91E-09

GO:0051047	BP	positive regulation of secretion	11	6.94E-09
GO:0045833	BP	negative regulation of lipid metabolic process	7	7.59E-09
GO:0010594	BP	regulation of endothelial cell migration	8	7.84E-09
GO:0008202	BP	steroid metabolic process	10	7.92E-09
GO:0032835	BP	glomerulus development	6	1.08E-08
GO:0048592	BP	eye morphogenesis	8	1.11E-08
GO:0050890	BP	cognition	10	1.12E-08
GO:0048732	BP	gland development	11	1.34E-08
GO:0055117	BP	regulation of cardiac muscle contraction	6	1.54E-08
GO:0086003	BP	cardiac muscle cell contraction	6	1.54E-08
GO:0043534	BP	blood vessel endothelial cell migration	7	1.54E-08
GO:0034765	BP	regulation of monoatomic ion transmembrane transport	11	1.62E-08
GO:0035249	BP	synaptic transmission, glutamatergic	7	1.82E-08
GO:0007409	BP	axonogenesis	11	1.83E-08
GO:0048662	BP	negative regulation of smooth muscle cell proliferation	6	1.97E-08
GO:0048863	BP	stem cell differentiation	9	2E-08
GO:0048659	BP	smooth muscle cell proliferation	8	2.04E-08
GO:1903532	BP	positive regulation of secretion by cell	10	2.09E-08
GO:0034767	BP	positive regulation of monoatomic ion transmembrane transport	8	2.56E-08
GO:2000027	BP	regulation of animal organ morphogenesis	7	2.64E-08
GO:0090049	BP	regulation of cell migration involved in sprouting angiogenesis	5	3.06E-08
GO:0048638	BP	regulation of developmental growth	10	3.06E-08
GO:2000242	BP	negative regulation of reproductive process	6	3.42E-08
GO:2000811	BP	negative regulation of anoikis	4	3.48E-08
GO:0060070	BP	canonical Wnt signaling pathway	9	3.48E-08
GO:0061448	BP	connective tissue development	9	3.68E-08
GO:0046165	BP	alcohol biosynthetic process	7	3.94E-08
GO:0060485	BP	mesenchyme development	9	4E-08
GO:0002067	BP	glandular epithelial cell differentiation	6	4.91E-08
GO:0002065	BP	columnar/cuboidal epithelial cell differentiation	7	5.24E-08
GO:0042886	BP	amide transport	10	5.24E-08
GO:0001666	BP	response to hypoxia	8	5.43E-08
GO:0050810	BP	regulation of steroid biosynthetic process	6	5.63E-08
GO:0051966	BP	regulation of synaptic transmission, glutamatergic	6	5.63E-08
GO:0014032	BP	neural crest cell development	6	6.03E-08
GO:0007611	BP	learning or memory	9	6.15E-08
GO:0051783	BP	regulation of nuclear division	7	6.3E-08
GO:0032768	BP	regulation of monooxygenase activity	5	7.19E-08
GO:0002685	BP	regulation of leukocyte migration	8	7.33E-08
GO:0050679	BP	positive regulation of epithelial cell proliferation	8	7.57E-08
GO:0009410	BP	response to xenobiotic stimulus	9	7.76E-08
GO:0022612	BP	gland morphogenesis	7	7.86E-08
GO:0002064	BP	epithelial cell development	8	8.89E-08
GO:0051651	BP	maintenance of location in cell	8	8.89E-08

GO:0097529	BP	myeloid leukocyte migration	8	8.89E-08
GO:0043535	BP	regulation of blood vessel endothelial cell migration	6	9.5E-08
GO:0016055	BP	Wnt signaling pathway	10	9.64E-08
GO:0007613	BP	memory	7	9.74E-08
GO:0198738	BP	cell-cell signaling by wnt	10	1E-07
GO:0033628	BP	regulation of cell adhesion mediated by integrin	5	1.1E-07
GO:0010632	BP	regulation of epithelial cell migration	8	1.11E-07
GO:0070252	BP	actin-mediated cell contraction	6	1.14E-07
GO:0099183	BP	trans-synaptic signaling by BDNF, modulating synaptic transmission	3	1.17E-07
GO:0060973	BP	cell migration involved in heart development	4	1.22E-07
GO:0030856	BP	regulation of epithelial cell differentiation	7	1.25E-07
GO:0006694	BP	steroid biosynthetic process	7	1.3E-07
GO:0036293	BP	response to decreased oxygen levels	8	1.33E-07
GO:0061005	BP	cell differentiation involved in kidney development	5	1.35E-07
GO:0014033	BP	neural crest cell differentiation	6	1.36E-07
GO:0009266	BP	response to temperature stimulus	7	1.41E-07
GO:0030850	BP	prostate gland development	5	1.49E-07
GO:0006941	BP	striated muscle contraction	7	1.53E-07
GO:0051235	BP	maintenance of location	9	1.57E-07
GO:0002042	BP	cell migration involved in sprouting angiogenesis	5	1.64E-07
GO:0042391	BP	regulation of membrane potential	10	1.73E-07
GO:0001657	BP	ureteric bud development	6	1.82E-07
GO:2000209	BP	regulation of anoikis	4	1.84E-07
GO:0072132	BP	mesenchyme morphogenesis	5	1.97E-07
GO:0045444	BP	fat cell differentiation	8	2.05E-07
GO:0045766	BP	positive regulation of angiogenesis	7	2.07E-07
GO:1904018	BP	positive regulation of vasculature development	7	2.07E-07
GO:0042310	BP	vasoconstriction	6	2.14E-07
GO:0072163	BP	mesonephric epithelium development	6	2.14E-07
GO:0072164	BP	mesonephric tubule development	6	2.14E-07
GO:2000243	BP	positive regulation of reproductive process	6	2.14E-07
GO:0042063	BP	gliogenesis	9	2.16E-07
GO:0032370	BP	positive regulation of lipid transport	6	2.26E-07
GO:0099191	BP	trans-synaptic signaling by BDNF	3	2.34E-07
GO:0007200	BP	phospholipase C-activating G protein-coupled receptor signaling pathway	6	2.38E-07
GO:0045834	BP	positive regulation of lipid metabolic process	7	2.41E-07
GO:0001823	BP	mesonephros development	6	2.51E-07
GO:0034764	BP	positive regulation of transmembrane transport	8	2.57E-07
GO:0050900	BP	leukocyte migration	9	2.57E-07
GO:0014074	BP	response to purine-containing compound	6	2.65E-07
GO:0019218	BP	regulation of steroid metabolic process	6	2.65E-07
GO:0048660	BP	regulation of smooth muscle cell proliferation	7	3.1E-07
GO:0010894	BP	negative regulation of steroid biosynthetic process	4	3.17E-07
GO:0046887	BP	positive regulation of hormone secretion	7	3.21E-07

GO:0060562	BP	epithelial tube morphogenesis	9	3.25E-07
GO:0048599	BP	oocyte development	5	3.3E-07
GO:0060560	BP	developmental growth involved in morphogenesis	8	3.53E-07
GO:0032964	BP	collagen biosynthetic process	5	3.58E-07
GO:0072009	BP	nephron epithelium development	6	3.58E-07
GO:0003007	BP	heart morphogenesis	8	3.63E-07
GO:0051924	BP	regulation of calcium ion transport	8	3.72E-07
GO:0045939	BP	negative regulation of steroid metabolic process	4	3.74E-07
GO:0032963	BP	collagen metabolic process	6	3.76E-07
GO:0046879	BP	hormone secretion	9	3.76E-07
GO:0003205	BP	cardiac chamber development	7	3.94E-07
GO:0019932	BP	second-messenger-mediated signaling	8	4.02E-07
GO:0008016	BP	regulation of heart contraction	7	4.08E-07
GO:0009994	BP	oocyte differentiation	5	4.19E-07
GO:0046620	BP	regulation of organ growth	6	4.35E-07
GO:0072012	BP	glomerulus vasculature development	4	4.38E-07
GO:0060688	BP	regulation of morphogenesis of a branching structure	5	4.52E-07
GO:0009914	BP	hormone transport	9	4.53E-07
GO:0051209	BP	release of sequestered calcium ion into cytosol	6	5.01E-07
GO:0003272	BP	endocardial cushion formation	4	5.1E-07
GO:0061437	BP	renal system vasculature development	4	5.1E-07
GO:0061440	BP	kidney vasculature development	4	5.1E-07
GO:0002040	BP	sprouting angiogenesis	6	5.25E-07
GO:0030048	BP	actin filament-based movement	6	5.25E-07
GO:0051283	BP	negative regulation of sequestering of calcium ion	6	5.25E-07
GO:0015850	BP	organic hydroxy compound transport	8	5.3E-07
GO:0060326	BP	cell chemotaxis	8	5.57E-07
GO:0001655	BP	urogenital system development	5	5.66E-07
GO:0051282	BP	regulation of sequestering of calcium ion	6	5.75E-07
GO:0046890	BP	regulation of lipid biosynthetic process	7	5.85E-07
GO:0086005	BP	ventricular cardiac muscle cell action potential	4	5.91E-07
GO:0060048	BP	cardiac muscle contraction	6	6.01E-07
GO:0032414	BP	positive regulation of ion transmembrane transporter activity	6	6.29E-07
GO:0051208	BP	sequestering of calcium ion	6	6.29E-07
GO:0070482	BP	response to oxygen levels	8	6.6E-07
GO:1905954	BP	positive regulation of lipid localization	6	6.87E-07
GO:0050769	BP	positive regulation of neurogenesis	8	7.09E-07
GO:0015711	BP	organic anion transport	9	7.24E-07
GO:0007204	BP	positive regulation of cytosolic calcium ion concentration	7	7.28E-07
GO:0035265	BP	organ growth	7	7.74E-07
GO:0048639	BP	positive regulation of developmental growth	7	7.74E-07
GO:0030198	BP	extracellular matrix organization	8	7.78E-07
GO:0043276	BP	anoikis	4	7.8E-07
GO:0045927	BP	positive regulation of growth	8	7.97E-07

GO:0043062	BP	extracellular structure organization	8	8.15E-07
GO:0045229	BP	external encapsulating structure organization	8	8.15E-07
GO:0001649	BP	osteoblast differentiation	7	8.73E-07
GO:0032411	BP	positive regulation of transporter activity	6	8.88E-07
GO:0048608	BP	reproductive structure development	8	9.14E-07
GO:0061045	BP	negative regulation of wound healing	5	9.19E-07
GO:0048645	BP	animal organ formation	5	9.81E-07
GO:0061458	BP	reproductive system development	8	1E-06
GO:0010574	BP	regulation of vascular endothelial growth factor production	4	1.01E-06
GO:0060740	BP	prostate gland epithelium morphogenesis	4	1.01E-06
GO:0046883	BP	regulation of hormone secretion	8	1.09E-06
GO:0050920	BP	regulation of chemotaxis	7	1.1E-06
GO:0003156	BP	regulation of animal organ formation	4	1.14E-06
GO:0050999	BP	regulation of nitric-oxide synthase activity	4	1.14E-06
GO:0060512	BP	prostate gland morphogenesis	4	1.14E-06
GO:1902074	BP	response to salt	8	1.17E-06
GO:0051928	BP	positive regulation of calcium ion transport	6	1.33E-06
GO:1904238	BP	pericyte cell differentiation	3	1.4E-06
GO:0043537	BP	negative regulation of blood vessel endothelial cell migration	4	1.45E-06
GO:0060047	BP	heart contraction	7	1.46E-06
GO:0050921	BP	positive regulation of chemotaxis	6	1.49E-06
GO:0001508	BP	action potential	6	1.55E-06
GO:0001936	BP	regulation of endothelial cell proliferation	6	1.55E-06
GO:0050767	BP	regulation of neurogenesis	9	1.57E-06
GO:0050878	BP	regulation of body fluid levels	8	1.58E-06
GO:0010573	BP	vascular endothelial growth factor production	4	1.62E-06
GO:0019229	BP	regulation of vasoconstriction	5	1.8E-06
GO:0001556	BP	oocyte maturation	4	1.81E-06
GO:0033629	BP	negative regulation of cell adhesion mediated by integrin	3	1.92E-06
GO:0086016	BP	AV node cell action potential	3	1.92E-06
GO:0086027	BP	AV node cell to bundle of His cell signaling	3	1.92E-06
GO:0045667	BP	regulation of osteoblast differentiation	6	1.94E-06
GO:0003203	BP	endocardial cushion morphogenesis	4	2.01E-06
GO:0045940	BP	positive regulation of steroid metabolic process	4	2.01E-06
GO:0086091	BP	regulation of heart rate by cardiac conduction	4	2.01E-06
GO:0046942	BP	carboxylic acid transport	8	2.06E-06
GO:0003015	BP	heart process	7	2.07E-06
GO:0097530	BP	granulocyte migration	6	2.08E-06
GO:0033627	BP	cell adhesion mediated by integrin	5	2.13E-06
GO:0015849	BP	organic acid transport	8	2.15E-06
GO:0002687	BP	positive regulation of leukocyte migration	6	2.23E-06
GO:1904062	BP	regulation of monoatomic cation transmembrane transport	8	2.24E-06
GO:0032368	BP	regulation of lipid transport	6	2.4E-06
GO:0035850	BP	epithelial cell differentiation involved in kidney development	4	2.47E-06

GO:0062012	BP	regulation of small molecule metabolic process	8	2.52E-06
GO:1990845	BP	adaptive thermogenesis	6	2.57E-06
GO:0051962	BP	positive regulation of nervous system development	8	2.62E-06
GO:0051899	BP	membrane depolarization	5	2.64E-06
GO:1903035	BP	negative regulation of response to wounding	5	2.64E-06
GO:0098703	BP	calcium ion import across plasma membrane	4	2.72E-06
GO:0110110	BP	positive regulation of animal organ morphogenesis	4	2.72E-06
GO:0001935	BP	endothelial cell proliferation	6	2.84E-06
GO:0060563	BP	neuroepithelial cell differentiation	4	3E-06
GO:0086002	BP	cardiac muscle cell action potential involved in contraction	4	3E-06
GO:0086065	BP	cell communication involved in cardiac conduction	4	3E-06
GO:0007292	BP	female gamete generation	6	3.15E-06
GO:1904064	BP	positive regulation of cation transmembrane transport	6	3.15E-06
GO:0001973	BP	G protein-coupled adenosine receptor signaling pathway	3	3.31E-06
GO:0035588	BP	G protein-coupled purinergic receptor signaling pathway	3	3.31E-06
GO:0086067	BP	AV node cell to bundle of His cell communication	3	3.31E-06
GO:0009408	BP	response to heat	5	3.41E-06
GO:0051341	BP	regulation of oxidoreductase activity	5	3.59E-06
GO:0002347	BP	response to tumor cell	4	3.94E-06
GO:0007596	BP	blood coagulation	6	4.21E-06
GO:1901214	BP	regulation of neuron death	8	4.27E-06
GO:0007599	BP	hemostasis	6	4.62E-06
GO:0050817	BP	coagulation	6	4.62E-06
GO:0032970	BP	regulation of actin filament-based process	8	4.76E-06
GO:0050770	BP	regulation of axonogenesis	6	4.76E-06
GO:0032412	BP	regulation of monoatomic ion transmembrane transporter activity	7	4.83E-06
GO:1903531	BP	negative regulation of secretion by cell	6	4.91E-06
GO:0060993	BP	kidney morphogenesis	5	5E-06
GO:0050714	BP	positive regulation of protein secretion	6	5.06E-06
GO:0030900	BP	forebrain development	8	5.2E-06
GO:0086014	BP	atrial cardiac muscle cell action potential	3	5.26E-06
GO:0086026	BP	atrial cardiac muscle cell to AV node cell signaling	3	5.26E-06
GO:0086066	BP	atrial cardiac muscle cell to AV node cell communication	3	5.26E-06
GO:1902337	BP	regulation of apoptotic process involved in morphogenesis	3	5.26E-06
GO:0099551	BP	trans-synaptic signaling by neuropeptide, modulating synaptic transmission	2	5.26E-06
GO:0043523	BP	regulation of neuron apoptotic process	7	5.28E-06
GO:0046889	BP	positive regulation of lipid biosynthetic process	5	5.48E-06
GO:1904427	BP	positive regulation of calcium ion transmembrane transport	5	5.48E-06
GO:0014706	BP	striated muscle tissue development	7	5.52E-06
GO:2001233	BP	regulation of apoptotic signaling pathway	8	5.97E-06
GO:1905952	BP	regulation of lipid localization	6	6.24E-06
GO:0001503	BP	ossification	8	6.29E-06
GO:0022898	BP	regulation of transmembrane transporter activity	7	6.43E-06
GO:0035590	BP	purinergic nucleotide receptor signaling pathway	3	6.46E-06

GO:0061101	BP	neuroendocrine cell differentiation	3	6.46E-06
GO:0072109	BP	glomerular mesangium development	3	6.46E-06
GO:1904748	BP	regulation of apoptotic process involved in development	3	6.46E-06
GO:0003197	BP	endocardial cushion development	4	6.47E-06
GO:0002027	BP	regulation of heart rate	5	6.53E-06
GO:1902930	BP	regulation of alcohol biosynthetic process	4	6.99E-06
GO:1903169	BP	regulation of calcium ion transmembrane transport	6	7.2E-06
GO:0032965	BP	regulation of collagen biosynthetic process	4	7.54E-06
GO:0048477	BP	oogenesis	5	7.74E-06
GO:0003198	BP	epithelial to mesenchymal transition involved in endocardial cushion formation	3	7.83E-06
GO:0099550	BP	trans-synaptic signaling, modulating synaptic transmission	3	7.83E-06
GO:0032409	BP	regulation of transporter activity	7	8.63E-06
GO:0010596	BP	negative regulation of endothelial cell migration	4	8.72E-06
GO:0070509	BP	calcium ion import	4	8.72E-06
GO:0006869	BP	lipid transport	8	8.77E-06
GO:1901652	BP	response to peptide	8	8.77E-06
GO:0007162	BP	negative regulation of cell adhesion	7	8.8E-06
GO:0030111	BP	regulation of Wnt signaling pathway	7	9.36E-06
GO:0048771	BP	tissue remodeling	6	9.76E-06
GO:0051952	BP	regulation of amine transport	5	9.88E-06
GO:0060419	BP	heart growth	5	1.03E-05
GO:0051055	BP	negative regulation of lipid biosynthetic process	4	1.08E-05
GO:0070997	BP	neuron death	8	1.1E-05
GO:0098659	BP	inorganic cation import across plasma membrane	5	1.11E-05
GO:0099587	BP	inorganic ion import across plasma membrane	5	1.11E-05
GO:0051048	BP	negative regulation of secretion	6	1.14E-05
GO:0055078	BP	sodium ion homeostasis	4	1.15E-05
GO:0051216	BP	cartilage development	6	1.17E-05
GO:0048562	BP	embryonic organ morphogenesis	7	1.21E-05
GO:0030858	BP	positive regulation of epithelial cell differentiation	4	1.23E-05
GO:0015837	BP	amine transport	5	1.25E-05
GO:0050708	BP	regulation of protein secretion	7	1.26E-05
GO:0051402	BP	neuron apoptotic process	7	1.28E-05
GO:0002011	BP	morphogenesis of an epithelial sheet	4	1.31E-05
GO:0010712	BP	regulation of collagen metabolic process	4	1.31E-05
GO:0086001	BP	cardiac muscle cell action potential	4	1.31E-05
GO:0071621	BP	granulocyte chemotaxis	5	1.34E-05
GO:1990266	BP	neutrophil migration	5	1.39E-05
GO:0034329	BP	cell junction assembly	8	1.4E-05
GO:0016042	BP	lipid catabolic process	7	1.41E-05
GO:0051222	BP	positive regulation of protein transport	7	1.44E-05
GO:0003214	BP	cardiac left ventricle morphogenesis	3	1.52E-05
GO:0043117	BP	positive regulation of vascular permeability	3	1.52E-05
GO:0090050	BP	positive regulation of cell migration involved in sprouting angiogenesis	3	1.52E-05

GO:0150104	BP	transport across blood-brain barrier	3	1.52E-05
GO:0062197	BP	cellular response to chemical stress	7	1.55E-05
GO:0090090	BP	negative regulation of canonical Wnt signaling pathway	5	1.55E-05
GO:0071228	BP	cellular response to tumor cell	2	1.58E-05
GO:0072011	BP	glomerular endothelium development	2	1.58E-05
GO:0098912	BP	membrane depolarization during atrial cardiac muscle cell action potential	2	1.58E-05
GO:0099540	BP	trans-synaptic signaling by neuropeptide	2	1.58E-05
GO:0060038	BP	cardiac muscle cell proliferation	4	1.58E-05
GO:0090497	BP	mesenchymal cell migration	4	1.58E-05
GO:0003014	BP	renal system process	5	1.61E-05
GO:0009636	BP	response to toxic substance	5	1.61E-05
GO:0009110	BP	vitamin biosynthetic process	3	1.76E-05
GO:0010232	BP	vascular transport	3	1.76E-05
GO:0071900	BP	regulation of protein serine/threonine kinase activity	7	1.76E-05
GO:1904951	BP	positive regulation of establishment of protein localization	7	1.93E-05
GO:0010876	BP	lipid localization	8	1.97E-05
GO:2001026	BP	regulation of endothelial cell chemotaxis	3	2.02E-05
GO:0097191	BP	extrinsic apoptotic signaling pathway	6	2.06E-05
GO:1901888	BP	regulation of cell junction assembly	6	2.06E-05
GO:0061337	BP	cardiac conduction	4	2.13E-05
GO:0008347	BP	glial cell migration	4	2.25E-05
GO:0030193	BP	regulation of blood coagulation	4	2.25E-05
GO:0098900	BP	regulation of action potential	4	2.25E-05
GO:0042445	BP	hormone metabolic process	6	2.26E-05
GO:2001234	BP	negative regulation of apoptotic signaling pathway	6	2.26E-05
GO:0048144	BP	fibroblast proliferation	5	2.34E-05
GO:0007193	BP	adenylate cyclase-inhibiting G protein-coupled receptor signaling pathway	4	2.38E-05
GO:0060828	BP	regulation of canonical Wnt signaling pathway	6	2.48E-05
GO:0120161	BP	regulation of cold-induced thermogenesis	5	2.5E-05
GO:1900046	BP	regulation of hemostasis	4	2.51E-05
GO:0050727	BP	regulation of inflammatory response	7	2.63E-05
GO:0010633	BP	negative regulation of epithelial cell migration	4	2.65E-05
GO:0002793	BP	positive regulation of peptide secretion	5	2.75E-05
GO:0003206	BP	cardiac chamber morphogenesis	5	2.75E-05
GO:0045598	BP	regulation of fat cell differentiation	5	2.75E-05
GO:0106106	BP	cold-induced thermogenesis	5	2.75E-05
GO:0050818	BP	regulation of coagulation	4	2.79E-05
GO:1904705	BP	regulation of vascular associated smooth muscle cell proliferation	4	2.79E-05
GO:0003231	BP	cardiac ventricle development	5	2.84E-05
GO:0010575	BP	positive regulation of vascular endothelial growth factor production	3	2.95E-05
GO:0048593	BP	camera-type eye morphogenesis	5	3.02E-05
GO:0050805	BP	negative regulation of synaptic transmission	4	3.1E-05
GO:0003273	BP	cell migration involved in endocardial cushion formation	2	3.15E-05
GO:0060686	BP	negative regulation of prostatic bud formation	2	3.15E-05

GO:0072008	BP	glomerular mesangial cell differentiation	2	3.15E-05
GO:0072144	BP	glomerular mesangial cell development	2	3.15E-05
GO:0001837	BP	epithelial to mesenchymal transition	5	3.31E-05
GO:0098657	BP	import into cell	6	3.36E-05
GO:1990874	BP	vascular associated smooth muscle cell proliferation	4	3.43E-05
GO:0003094	BP	glomerular filtration	3	3.7E-05
GO:0008209	BP	androgen metabolic process	3	3.7E-05
GO:0043279	BP	response to alkaloid	4	3.97E-05
GO:1901215	BP	negative regulation of neuron death	6	4.05E-05
GO:0010893	BP	positive regulation of steroid biosynthetic process	3	4.12E-05
GO:0032770	BP	positive regulation of monooxygenase activity	3	4.12E-05
GO:0060045	BP	positive regulation of cardiac muscle cell proliferation	3	4.12E-05
GO:0098901	BP	regulation of cardiac muscle cell action potential	3	4.12E-05
GO:0010001	BP	glial cell differentiation	6	4.14E-05
GO:0003208	BP	cardiac ventricle morphogenesis	4	4.16E-05
GO:0048844	BP	artery morphogenesis	4	4.16E-05
GO:0048738	BP	cardiac muscle tissue development	6	4.31E-05
GO:0014855	BP	striated muscle cell proliferation	4	4.36E-05
GO:0007178	BP	transmembrane receptor protein serine/threonine kinase signaling pathway	7	4.41E-05
GO:0007043	BP	cell-cell junction assembly	5	4.43E-05
GO:0030178	BP	negative regulation of Wnt signaling pathway	5	4.43E-05
GO:0050680	BP	negative regulation of epithelial cell proliferation	5	4.43E-05
GO:2001236	BP	regulation of extrinsic apoptotic signaling pathway	5	4.56E-05
GO:0048596	BP	embryonic camera-type eye morphogenesis	3	4.57E-05
GO:0086019	BP	cell-cell signaling involved in cardiac conduction	3	4.57E-05
GO:0097205	BP	renal filtration	3	4.57E-05
GO:1902932	BP	positive regulation of alcohol biosynthetic process	3	4.57E-05
GO:0031667	BP	response to nutrient levels	7	4.76E-05
GO:0006809	BP	nitric oxide biosynthetic process	4	4.79E-05
GO:0034308	BP	primary alcohol metabolic process	4	4.79E-05
GO:0048015	BP	phosphatidylinositol-mediated signaling	5	4.82E-05
GO:0071383	BP	cellular response to steroid hormone stimulus	5	4.82E-05
GO:0051098	BP	regulation of binding	7	4.91E-05
GO:0035767	BP	endothelial cell chemotaxis	3	5.06E-05
GO:0060561	BP	apoptotic process involved in morphogenesis	3	5.06E-05
GO:0086010	BP	membrane depolarization during action potential	3	5.06E-05
GO:1905523	BP	positive regulation of macrophage migration	3	5.06E-05
GO:0003241	BP	growth involved in heart morphogenesis	2	5.23E-05
GO:0010957	BP	negative regulation of vitamin D biosynthetic process	2	5.23E-05
GO:0072007	BP	mesangial cell differentiation	2	5.23E-05
GO:0072143	BP	mesangial cell development	2	5.23E-05
GO:0086045	BP	membrane depolarization during AV node cell action potential	2	5.23E-05
GO:0003151	BP	outflow tract morphogenesis	4	5.24E-05
GO:0048017	BP	inositol lipid-mediated signaling	5	5.24E-05

GO:0010623	BP	programmed cell death involved in cell development	3	5.57E-05
GO:0060317	BP	cardiac epithelial to mesenchymal transition	3	5.57E-05
GO:0060384	BP	innervation	3	5.57E-05
GO:0072028	BP	nephron morphogenesis	4	5.72E-05
GO:0019221	BP	cytokine-mediated signaling pathway	7	5.96E-05
GO:0032102	BP	negative regulation of response to external stimulus	7	5.96E-05
GO:0001656	BP	metanephros development	4	5.97E-05
GO:0060420	BP	regulation of heart growth	4	5.97E-05
GO:0010875	BP	positive regulation of cholesterol efflux	3	6.12E-05
GO:0046209	BP	nitric oxide metabolic process	4	6.23E-05
GO:0062013	BP	positive regulation of small molecule metabolic process	5	6.48E-05
GO:0021675	BP	nerve development	4	6.5E-05
GO:0051937	BP	catecholamine transport	4	6.5E-05
GO:0060306	BP	regulation of membrane repolarization	3	6.7E-05
GO:1904706	BP	negative regulation of vascular associated smooth muscle cell proliferation	3	6.7E-05
GO:0042490	BP	mechanoreceptor differentiation	4	6.78E-05
GO:2001057	BP	reactive nitrogen species metabolic process	4	6.78E-05
GO:0009306	BP	protein secretion	7	6.79E-05
GO:0007219	BP	Notch signaling pathway	5	6.83E-05
GO:0035592	BP	establishment of protein localization to extracellular region	7	6.88E-05
GO:0007369	BP	gastrulation	5	7.01E-05
GO:0035148	BP	tube formation	5	7.01E-05
GO:0046683	BP	response to organophosphorus	4	7.07E-05
GO:0010863	BP	positive regulation of phospholipase C activity	3	7.31E-05
GO:0014047	BP	glutamate secretion	3	7.31E-05
GO:0071692	BP	protein localization to extracellular region	7	7.49E-05
GO:0016331	BP	morphogenesis of embryonic epithelium	5	7.56E-05
GO:0009991	BP	response to extracellular stimulus	7	7.71E-05
GO:0007231	BP	osmosensory signaling pathway	2	7.84E-05
GO:0035166	BP	post-embryonic hemopoiesis	2	7.84E-05
GO:0046137	BP	negative regulation of vitamin metabolic process	2	7.84E-05
GO:0060129	BP	thyroid-stimulating hormone-secreting cell differentiation	2	7.84E-05
GO:0060685	BP	regulation of prostatic bud formation	2	7.84E-05
GO:0060687	BP	regulation of branching involved in prostate gland morphogenesis	2	7.84E-05
GO:2000324	BP	positive regulation of glucocorticoid receptor signaling pathway	2	7.84E-05
GO:0032967	BP	positive regulation of collagen biosynthetic process	3	7.97E-05
GO:0048048	BP	embryonic eye morphogenesis	3	7.97E-05
GO:0090183	BP	regulation of kidney development	3	7.97E-05
GO:0090075	BP	relaxation of muscle	3	8.65E-05
GO:0000280	BP	nuclear division	7	8.73E-05
GO:0048511	BP	rhythmic process	6	8.78E-05
GO:0042692	BP	muscle cell differentiation	7	9.21E-05
GO:0019935	BP	cyclic-nucleotide-mediated signaling	4	9.32E-05
GO:0030168	BP	platelet activation	4	9.32E-05

GO:0050772	BP	positive regulation of axonogenesis	4	9.32E-05
GO:0033273	BP	response to vitamin	3	9.38E-05
GO:0051953	BP	negative regulation of amine transport	3	9.38E-05
GO:1902003	BP	regulation of amyloid-beta formation	3	9.38E-05
GO:0006970	BP	response to osmotic stress	4	9.68E-05
GO:0043524	BP	negative regulation of neuron apoptotic process	5	9.88E-05
GO:0010714	BP	positive regulation of collagen metabolic process	3	0.000101
GO:0019934	BP	cGMP-mediated signaling	3	0.000101
GO:0040020	BP	regulation of meiotic nuclear division	3	0.000101
GO:1900274	BP	regulation of phospholipase C activity	3	0.000101
GO:0030593	BP	neutrophil chemotaxis	4	0.000104
GO:0072080	BP	nephron tubule development	4	0.000104
GO:0043405	BP	regulation of MAP kinase activity	5	0.000106
GO:0001938	BP	positive regulation of endothelial cell proliferation	4	0.000108
GO:0090288	BP	negative regulation of cellular response to growth factor stimulus	4	0.000108
GO:1905332	BP	positive regulation of morphogenesis of an epithelium	3	0.00011
GO:0046668	BP	regulation of retinal cell programmed cell death	2	0.00011
GO:0060556	BP	regulation of vitamin D biosynthetic process	2	0.00011
GO:0060745	BP	mammary gland branching involved in pregnancy	2	0.00011
GO:1902338	BP	negative regulation of apoptotic process involved in morphogenesis	2	0.00011
GO:0002790	BP	peptide secretion	6	0.000111
GO:0001704	BP	formation of primary germ layer	4	0.000112
GO:1901654	BP	response to ketone	5	0.000116
GO:0043588	BP	skin development	6	0.000117
GO:0030431	BP	sleep	3	0.000118
GO:1903670	BP	regulation of sprouting angiogenesis	3	0.000118
GO:0031334	BP	positive regulation of protein-containing complex assembly	5	0.000122
GO:0098739	BP	import across plasma membrane	5	0.000122
GO:1901653	BP	cellular response to peptide	6	0.000123
GO:2000181	BP	negative regulation of blood vessel morphogenesis	4	0.000125
GO:0031076	BP	embryonic camera-type eye development	3	0.000127
GO:0051968	BP	positive regulation of synaptic transmission, glutamatergic	3	0.000127
GO:0010595	BP	positive regulation of endothelial cell migration	4	0.000129
GO:0019751	BP	polyol metabolic process	4	0.000129
GO:1901343	BP	negative regulation of vasculature development	4	0.000129
GO:0061326	BP	renal tubule development	4	0.000134
GO:0062014	BP	negative regulation of small molecule metabolic process	4	0.000134
GO:0007616	BP	long-term memory	3	0.000136
GO:0014909	BP	smooth muscle cell migration	4	0.000139
GO:0015844	BP	monoamine transport	4	0.000139
GO:0007389	BP	pattern specification process	7	0.00014
GO:0015833	BP	peptide transport	6	0.00014
GO:0032024	BP	positive regulation of insulin secretion	4	0.000143
GO:0010874	BP	regulation of cholesterol efflux	3	0.000146

GO:0031128	BP	developmental induction	3	0.000146
GO:0048730	BP	epidermis morphogenesis	3	0.000146
GO:0010700	BP	negative regulation of norepinephrine secretion	2	0.000146
GO:0031547	BP	brain-derived neurotrophic factor receptor signaling pathway	2	0.000146
GO:0032730	BP	positive regulation of interleukin-1 alpha production	2	0.000146
GO:0045899	BP	positive regulation of RNA polymerase II transcription preinitiation complex assembly	2	0.000146
GO:0060087	BP	relaxation of vascular associated smooth muscle	2	0.000146
GO:0060353	BP	regulation of cell adhesion molecule production	2	0.000146
GO:0061314	BP	Notch signaling involved in heart development	2	0.000146
GO:1904746	BP	negative regulation of apoptotic process involved in development	2	0.000146
GO:1905331	BP	negative regulation of morphogenesis of an epithelium	2	0.000146
GO:1990962	BP	xenobiotic transport across blood-brain barrier	2	0.000146
GO:0071216	BP	cellular response to biotic stimulus	6	0.000147
GO:0055017	BP	cardiac muscle tissue growth	4	0.000148
GO:0043266	BP	regulation of potassium ion transport	4	0.000153
GO:0001569	BP	branching involved in blood vessel morphogenesis	3	0.000156
GO:0086009	BP	membrane repolarization	3	0.000156
GO:1902742	BP	apoptotic process involved in development	3	0.000156
GO:1902991	BP	regulation of amyloid precursor protein catabolic process	3	0.000156
GO:0071214	BP	cellular response to abiotic stimulus	6	0.000156
GO:0104004	BP	cellular response to environmental stimulus	6	0.000156
GO:0043401	BP	steroid hormone mediated signaling pathway	4	0.000158
GO:0007088	BP	regulation of mitotic nuclear division	4	0.000164
GO:0021987	BP	cerebral cortex development	4	0.000164
GO:0071346	BP	cellular response to type II interferon	4	0.000164
GO:0008217	BP	regulation of blood pressure	5	0.000165
GO:0001937	BP	negative regulation of endothelial cell proliferation	3	0.000167
GO:0032373	BP	positive regulation of sterol transport	3	0.000167
GO:0032376	BP	positive regulation of cholesterol transport	3	0.000167
GO:0060840	BP	artery development	4	0.000169
GO:0043114	BP	regulation of vascular permeability	3	0.000178
GO:0055023	BP	positive regulation of cardiac muscle tissue growth	3	0.000178
GO:0120163	BP	negative regulation of cold-induced thermogenesis	3	0.000178
GO:0007623	BP	circadian rhythm	5	0.000179
GO:0071675	BP	regulation of mononuclear cell migration	4	0.000186
GO:0003344	BP	pericardium morphogenesis	2	0.000187
GO:0032229	BP	negative regulation of synaptic transmission, GABAergic	2	0.000187
GO:0034651	BP	cortisol biosynthetic process	2	0.000187
GO:0042368	BP	vitamin D biosynthetic process	2	0.000187
GO:1902645	BP	tertiary alcohol biosynthetic process	2	0.000187
GO:1905276	BP	regulation of epithelial tube formation	2	0.000187
GO:1905278	BP	positive regulation of epithelial tube formation	2	0.000187
GO:0001941	BP	postsynaptic membrane organization	3	0.000189
GO:0034205	BP	amyloid-beta formation	3	0.000189

GO:0048010	BP	vascular endothelial growth factor receptor signaling pathway	3	0.000189
GO:0030595	BP	leukocyte chemotaxis	5	0.000194
GO:0045216	BP	cell-cell junction organization	5	0.000194
GO:1905521	BP	regulation of macrophage migration	3	0.000201
GO:0048145	BP	regulation of fibroblast proliferation	4	0.000211
GO:2000177	BP	regulation of neural precursor cell proliferation	4	0.000211
GO:0043647	BP	inositol phosphate metabolic process	3	0.000214
GO:0060421	BP	positive regulation of heart growth	3	0.000214
GO:1901890	BP	positive regulation of cell junction assembly	4	0.000217
GO:0007249	BP	I-kappaB kinase/NF-kappaB signaling	5	0.000219
GO:0014812	BP	muscle cell migration	4	0.000224
GO:0030195	BP	negative regulation of blood coagulation	3	0.000227
GO:0060043	BP	regulation of cardiac muscle cell proliferation	3	0.000227
GO:0046486	BP	glycerolipid metabolic process	6	0.000227
GO:0003253	BP	cardiac neural crest cell migration involved in outflow tract morphogenesis	2	0.000234
GO:0046666	BP	retinal cell programmed cell death	2	0.000234
GO:0060513	BP	prostatic bud formation	2	0.000234
GO:0099538	BP	synaptic signaling via neuropeptide	2	0.000234
GO:2000344	BP	positive regulation of acrosome reaction	2	0.000234
GO:0007517	BP	muscle organ development	6	0.000237
GO:2000278	BP	regulation of DNA biosynthetic process	4	0.000237
GO:0010518	BP	positive regulation of phospholipase activity	3	0.00024
GO:0010718	BP	positive regulation of epithelial to mesenchymal transition	3	0.00024
GO:0045907	BP	positive regulation of vasoconstriction	3	0.00024
GO:1900047	BP	negative regulation of hemostasis	3	0.00024
GO:0050819	BP	negative regulation of coagulation	3	0.000254
GO:0009913	BP	epidermal cell differentiation	5	0.000255
GO:0045664	BP	regulation of neuron differentiation	5	0.000255
GO:0006813	BP	potassium ion transport	5	0.00026
GO:0000086	BP	G2/M transition of mitotic cell cycle	4	0.000266
GO:0007588	BP	excretion	3	0.000269
GO:0043434	BP	response to peptide hormone	6	0.000269
GO:0003158	BP	endothelium development	4	0.000274
GO:0008544	BP	epidermis development	6	0.000276
GO:0034605	BP	cellular response to heat	3	0.000284
GO:0002246	BP	wound healing involved in inflammatory response	2	0.000285
GO:0032342	BP	aldosterone biosynthetic process	2	0.000285
GO:0045898	BP	regulation of RNA polymerase II transcription preinitiation complex assembly	2	0.000285
GO:0060352	BP	cell adhesion molecule production	2	0.000285
GO:0071372	BP	cellular response to follicle-stimulating hormone stimulus	2	0.000285
GO:0072075	BP	metanephric mesenchyme development	2	0.000285
GO:1990961	BP	xenobiotic detoxification by transmembrane export across the plasma membrane	2	0.000285
GO:0048872	BP	homeostasis of number of cells	6	0.000288
GO:0050435	BP	amyloid-beta metabolic process	3	0.000299

GO:0055010	BP	ventricular cardiac muscle tissue morphogenesis	3	0.000299
GO:1903170	BP	negative regulation of calcium ion transmembrane transport	3	0.000299
GO:0007411	BP	axon guidance	5	0.000301
GO:0072089	BP	stem cell proliferation	4	0.000306
GO:0045055	BP	regulated exocytosis	5	0.000307
GO:0097485	BP	neuron projection guidance	5	0.000307
GO:0043583	BP	ear development	5	0.000313
GO:0014065	BP	phosphatidylinositol 3-kinase signaling	4	0.000315
GO:1902692	BP	regulation of neuroblast proliferation	3	0.000315
GO:0030857	BP	negative regulation of epithelial cell differentiation	3	0.000332
GO:0043536	BP	positive regulation of blood vessel endothelial cell migration	3	0.000332
GO:0006705	BP	mineralocorticoid biosynthetic process	2	0.000342
GO:0014824	BP	artery smooth muscle contraction	2	0.000342
GO:0014832	BP	urinary bladder smooth muscle contraction	2	0.000342
GO:0032354	BP	response to follicle-stimulating hormone	2	0.000342
GO:0032650	BP	regulation of interleukin-1 alpha production	2	0.000342
GO:0033625	BP	positive regulation of integrin activation	2	0.000342
GO:0034650	BP	cortisol metabolic process	2	0.000342
GO:0042362	BP	fat-soluble vitamin biosynthetic process	2	0.000342
GO:0045607	BP	regulation of inner ear auditory receptor cell differentiation	2	0.000342
GO:0045631	BP	regulation of mechanoreceptor differentiation	2	0.000342
GO:0060525	BP	prostate glandular acinus development	2	0.000342
GO:0060536	BP	cartilage morphogenesis	2	0.000342
GO:0090184	BP	positive regulation of kidney development	2	0.000342
GO:0090209	BP	negative regulation of triglyceride metabolic process	2	0.000342
GO:0098911	BP	regulation of ventricular cardiac muscle cell action potential	2	0.000342
GO:1900272	BP	negative regulation of long-term synaptic potentiation	2	0.000342
GO:2000322	BP	regulation of glucocorticoid receptor signaling pathway	2	0.000342
GO:2000980	BP	regulation of inner ear receptor cell differentiation	2	0.000342
GO:0030522	BP	intracellular receptor signaling pathway	5	0.000348
GO:0051445	BP	regulation of meiotic cell cycle	3	0.000349
GO:0140353	BP	lipid export from cell	3	0.000349
GO:0035270	BP	endocrine system development	4	0.00035
GO:0048675	BP	axon extension	4	0.00035
GO:0006979	BP	response to oxidative stress	6	0.000361
GO:0042987	BP	amyloid precursor protein catabolic process	3	0.000367
GO:0045839	BP	negative regulation of mitotic nuclear division	3	0.000367
GO:0051353	BP	positive regulation of oxidoreductase activity	3	0.000367
GO:0060443	BP	mammary gland morphogenesis	3	0.000367
GO:0071622	BP	regulation of granulocyte chemotaxis	3	0.000367
GO:0034341	BP	response to type II interferon	4	0.000369
GO:0001974	BP	blood vessel remodeling	3	0.000385
GO:0030199	BP	collagen fibril organization	3	0.000385
GO:0048706	BP	embryonic skeletal system development	4	0.000389

GO:0090277	BP	positive regulation of peptide hormone secretion	4	0.000389
GO:0002791	BP	regulation of peptide secretion	5	0.000399
GO:0032610	BP	interleukin-1 alpha production	2	0.000403
GO:0034374	BP	low-density lipoprotein particle remodeling	2	0.000403
GO:0044557	BP	relaxation of smooth muscle	2	0.000403
GO:0046618	BP	xenobiotic export from cell	2	0.000403
GO:0048484	BP	enteric nervous system development	2	0.000403
GO:0060601	BP	lateral sprouting from an epithelium	2	0.000403
GO:0086015	BP	SA node cell action potential	2	0.000403
GO:0086018	BP	SA node cell to atrial cardiac muscle cell signaling	2	0.000403
GO:0086070	BP	SA node cell to atrial cardiac muscle cell communication	2	0.000403
GO:0044839	BP	cell cycle G2/M phase transition	4	0.000409
GO:0090087	BP	regulation of peptide transport	5	0.000413
GO:0034763	BP	negative regulation of transmembrane transport	4	0.000419
GO:0007159	BP	leukocyte cell-cell adhesion	6	0.000421
GO:0001755	BP	neural crest cell migration	3	0.000424
GO:0043407	BP	negative regulation of MAP kinase activity	3	0.000424
GO:0046622	BP	positive regulation of organ growth	3	0.000424
GO:0090303	BP	positive regulation of wound healing	3	0.000424
GO:2001257	BP	regulation of cation channel activity	4	0.00043
GO:0010517	BP	regulation of phospholipase activity	3	0.000444
GO:0042311	BP	vasodilation	3	0.000444
GO:0046173	BP	polyol biosynthetic process	3	0.000444
GO:0050873	BP	brown fat cell differentiation	3	0.000444
GO:0060193	BP	positive regulation of lipase activity	3	0.000444
GO:0006081	BP	cellular aldehyde metabolic process	3	0.000465
GO:0033344	BP	cholesterol efflux	3	0.000465
GO:0051785	BP	positive regulation of nuclear division	3	0.000465
GO:0071470	BP	cellular response to osmotic stress	3	0.000465
GO:0001660	BP	fever generation	2	0.00047
GO:0003207	BP	cardiac chamber formation	2	0.00047
GO:0007343	BP	egg activation	2	0.00047
GO:0014820	BP	tonic smooth muscle contraction	2	0.00047
GO:0032341	BP	aldosterone metabolic process	2	0.00047
GO:0034309	BP	primary alcohol biosynthetic process	2	0.00047
GO:0034375	BP	high-density lipoprotein particle remodeling	2	0.00047
GO:0051967	BP	negative regulation of synaptic transmission, glutamatergic	2	0.00047
GO:0061309	BP	cardiac neural crest cell development involved in outflow tract morphogenesis	2	0.00047
GO:0072044	BP	collecting duct development	2	0.00047
GO:0098917	BP	retrograde trans-synaptic signaling	2	0.00047
GO:0043271	BP	negative regulation of monoatomic ion transport	4	0.000486
GO:0003229	BP	ventricular cardiac muscle tissue development	3	0.000486
GO:0043254	BP	regulation of protein-containing complex assembly	6	0.000506
GO:0001658	BP	branching involved in ureteric bud morphogenesis	3	0.000508

GO:1905517	BP	macrophage migration	3	0.000508
GO:0001707	BP	mesoderm formation	3	0.00053
GO:0030330	BP	DNA damage response, signal transduction by p53 class mediator	3	0.00053
GO:0070265	BP	necrotic cell death	3	0.00053
GO:1903305	BP	regulation of regulated secretory pathway	4	0.000534
GO:0048588	BP	developmental cell growth	5	0.000536
GO:0014848	BP	urinary tract smooth muscle contraction	2	0.000541
GO:0032308	BP	positive regulation of prostaglandin secretion	2	0.000541
GO:0043518	BP	negative regulation of DNA damage response, signal transduction by p53 class mediator	2	0.000541
GO:0045188	BP	regulation of circadian sleep/wake cycle, non-REM sleep	2	0.000541
GO:0060046	BP	regulation of acrosome reaction	2	0.000541
GO:0060442	BP	branching involved in prostate gland morphogenesis	2	0.000541
GO:0072074	BP	kidney mesenchyme development	2	0.000541
GO:0097084	BP	vascular associated smooth muscle cell development	2	0.000541
GO:0010634	BP	positive regulation of epithelial cell migration	4	0.000547
GO:0071375	BP	cellular response to peptide hormone stimulus	5	0.000553
GO:0050433	BP	regulation of catecholamine secretion	3	0.000554
GO:0009755	BP	hormone-mediated signaling pathway	4	0.000559
GO:0006940	BP	regulation of smooth muscle contraction	3	0.000577
GO:0015800	BP	acidic amino acid transport	3	0.000577
GO:0045600	BP	positive regulation of fat cell differentiation	3	0.000577
GO:0051480	BP	regulation of cytosolic calcium ion concentration	3	0.000577
GO:0051784	BP	negative regulation of nuclear division	3	0.000577
GO:0051090	BP	regulation of DNA-binding transcription factor activity	6	0.000584
GO:0030879	BP	mammary gland development	4	0.000585
GO:0003002	BP	regionalization	6	0.000598
GO:0032371	BP	regulation of sterol transport	3	0.000602
GO:0032374	BP	regulation of cholesterol transport	3	0.000602
GO:0098754	BP	detoxification	3	0.000602
GO:0008212	BP	mineralocorticoid metabolic process	2	0.000618
GO:0010832	BP	negative regulation of myotube differentiation	2	0.000618
GO:0021984	BP	adenohypophysis development	2	0.000618
GO:0033280	BP	response to vitamin D	2	0.000618
GO:0033700	BP	phospholipid efflux	2	0.000618
GO:0042359	BP	vitamin D metabolic process	2	0.000618
GO:0042748	BP	circadian sleep/wake cycle, non-REM sleep	2	0.000618
GO:0042921	BP	glucocorticoid receptor signaling pathway	2	0.000618
GO:0043084	BP	penile erection	2	0.000618
GO:0046415	BP	urate metabolic process	2	0.000618
GO:0071371	BP	cellular response to gonadotropin stimulus	2	0.000618
GO:0086103	BP	G protein-coupled receptor signaling pathway involved in heart process	2	0.000618
GO:0090051	BP	negative regulation of cell migration involved in sprouting angiogenesis	2	0.000618
GO:0007584	BP	response to nutrient	3	0.000627
GO:0048332	BP	mesoderm morphogenesis	3	0.000627

GO:0034250	BP	positive regulation of amide metabolic process	4	0.00064
GO:0120254	BP	olefinic compound metabolic process	4	0.00064
GO:0045428	BP	regulation of nitric oxide biosynthetic process	3	0.000653
GO:0055008	BP	cardiac muscle tissue morphogenesis	3	0.000653
GO:0072175	BP	epithelial tube formation	4	0.000655
GO:0031346	BP	positive regulation of cell projection organization	6	0.000671
GO:0048678	BP	response to axon injury	3	0.000679
GO:0051926	BP	negative regulation of calcium ion transport	3	0.000679
GO:0060675	BP	ureteric bud morphogenesis	3	0.000679
GO:0045165	BP	cell fate commitment	5	0.000684
GO:0021543	BP	pallium development	4	0.000699
GO:0043433	BP	negative regulation of DNA-binding transcription factor activity	4	0.000699
GO:0006704	BP	glucocorticoid biosynthetic process	2	0.000699
GO:0031958	BP	corticosteroid receptor signaling pathway	2	0.000699
GO:0032306	BP	regulation of prostaglandin secretion	2	0.000699
GO:0055089	BP	fatty acid homeostasis	2	0.000699
GO:0061308	BP	cardiac neural crest cell development involved in heart development	2	0.000699
GO:0071243	BP	cellular response to arsenic-containing substance	2	0.000699
GO:0071498	BP	cellular response to fluid shear stress	2	0.000699
GO:0086012	BP	membrane depolarization during cardiac muscle cell action potential	2	0.000699
GO:0097152	BP	mesenchymal cell apoptotic process	2	0.000699
GO:2001028	BP	positive regulation of endothelial cell chemotaxis	2	0.000699
GO:0071695	BP	anatomical structure maturation	5	0.000705
GO:0017156	BP	calcium-ion regulated exocytosis	3	0.000706
GO:2001259	BP	positive regulation of cation channel activity	3	0.000706
GO:0042982	BP	amyloid precursor protein metabolic process	3	0.000734
GO:0050432	BP	catecholamine secretion	3	0.000734
GO:0051896	BP	regulation of protein kinase B signaling	4	0.000745
GO:0045669	BP	positive regulation of osteoblast differentiation	3	0.000763
GO:0080164	BP	regulation of nitric oxide metabolic process	3	0.000763
GO:0030656	BP	regulation of vitamin metabolic process	2	0.000786
GO:0032957	BP	inositol trisphosphate metabolic process	2	0.000786
GO:0033080	BP	immature T cell proliferation in thymus	2	0.000786
GO:0033623	BP	regulation of integrin activation	2	0.000786
GO:0034698	BP	response to gonadotropin	2	0.000786
GO:0046184	BP	aldehyde biosynthetic process	2	0.000786
GO:0047484	BP	regulation of response to osmotic stress	2	0.000786
GO:0060572	BP	morphogenesis of an epithelial bud	2	0.000786
GO:0061307	BP	cardiac neural crest cell differentiation involved in heart development	2	0.000786
GO:0061318	BP	renal filtration cell differentiation	2	0.000786
GO:0072077	BP	renal vesicle morphogenesis	2	0.000786
GO:0072112	BP	podocyte differentiation	2	0.000786
GO:0072311	BP	glomerular epithelial cell differentiation	2	0.000786
GO:0080154	BP	regulation of fertilization	2	0.000786

GO:0072171	BP	mesonephric tubule morphogenesis	3	0.000792
GO:0019722	BP	calcium-mediated signaling	4	0.000794
GO:0043627	BP	response to estrogen	3	0.000822
GO:0048146	BP	positive regulation of fibroblast proliferation	3	0.000822
GO:0061180	BP	mammary gland epithelium development	3	0.000822
GO:0071347	BP	cellular response to interleukin-1	3	0.000822
GO:0030509	BP	BMP signaling pathway	4	0.000827
GO:0031214	BP	biomineral tissue development	4	0.000827
GO:0055088	BP	lipid homeostasis	4	0.000845
GO:0010721	BP	negative regulation of cell development	5	0.00085
GO:0038034	BP	signal transduction in absence of ligand	3	0.000852
GO:0097192	BP	extrinsic apoptotic signaling pathway in absence of ligand	3	0.000852
GO:1903036	BP	positive regulation of response to wounding	3	0.000852
GO:2000573	BP	positive regulation of DNA biosynthetic process	3	0.000852
GO:0030072	BP	peptide hormone secretion	5	0.000874
GO:0090287	BP	regulation of cellular response to growth factor stimulus	5	0.000874
GO:0010544	BP	negative regulation of platelet activation	2	0.000877
GO:0033079	BP	immature T cell proliferation	2	0.000877
GO:0033145	BP	positive regulation of intracellular steroid hormone receptor signaling pathway	2	0.000877
GO:0033604	BP	negative regulation of catecholamine secretion	2	0.000877
GO:0051000	BP	positive regulation of nitric-oxide synthase activity	2	0.000877
GO:0060231	BP	mesenchymal to epithelial transition	2	0.000877
GO:1902430	BP	negative regulation of amyloid-beta formation	2	0.000877
GO:0009612	BP	response to mechanical stimulus	4	0.000897
GO:0071674	BP	mononuclear cell migration	4	0.000897
GO:0007548	BP	sex differentiation	5	0.000899
GO:0097193	BP	intrinsic apoptotic signaling pathway	5	0.000899
GO:0015718	BP	monocarboxylic acid transport	4	0.000916
GO:0071897	BP	DNA biosynthetic process	4	0.000916
GO:0008543	BP	fibroblast growth factor receptor signaling pathway	3	0.000916
GO:0051145	BP	smooth muscle cell differentiation	3	0.000916
GO:0051279	BP	regulation of release of sequestered calcium ion into cytosol	3	0.000916
GO:0043409	BP	negative regulation of MAPK cascade	4	0.000934
GO:0071356	BP	cellular response to tumor necrosis factor	4	0.000934
GO:0006766	BP	vitamin metabolic process	3	0.000949
GO:0016358	BP	dendrite development	5	0.000963
GO:0071496	BP	cellular response to external stimulus	5	0.000963
GO:0014061	BP	regulation of norepinephrine secretion	2	0.000973
GO:0032495	BP	response to muramyl dipeptide	2	0.000973
GO:0040037	BP	negative regulation of fibroblast growth factor receptor signaling pathway	2	0.000973
GO:0043116	BP	negative regulation of vascular permeability	2	0.000973
GO:0043691	BP	reverse cholesterol transport	2	0.000973
GO:0045986	BP	negative regulation of smooth muscle contraction	2	0.000973
GO:0048148	BP	behavioral response to cocaine	2	0.000973

GO:0060841	BP	venous blood vessel development	2	0.000973
GO:0072010	BP	glomerular epithelium development	2	0.000973
GO:0072087	BP	renal vesicle development	2	0.000973
GO:0099509	BP	regulation of presynaptic cytosolic calcium ion concentration	2	0.000973
GO:1902004	BP	positive regulation of amyloid-beta formation	2	0.000973
GO:1902644	BP	tertiary alcohol metabolic process	2	0.000973
GO:0055021	BP	regulation of cardiac muscle tissue growth	3	0.000982
GO:0007612	BP	learning	4	0.000991
GO:0060191	BP	regulation of lipase activity	3	0.001016
GO:0071772	BP	response to BMP	4	0.00105
GO:0071773	BP	cellular response to BMP stimulus	4	0.00105
GO:0072078	BP	nephron tubule morphogenesis	3	0.001051
GO:0043122	BP	regulation of I-kappaB kinase/NF-kappaB signaling	4	0.00107
GO:0006700	BP	C21-steroid hormone biosynthetic process	2	0.001073
GO:0032310	BP	prostaglandin secretion	2	0.001073
GO:0045540	BP	regulation of cholesterol biosynthetic process	2	0.001073
GO:0045836	BP	positive regulation of meiotic nuclear division	2	0.001073
GO:0048243	BP	norepinephrine secretion	2	0.001073
GO:0048569	BP	post-embryonic animal organ development	2	0.001073
GO:0090594	BP	inflammatory response to wounding	2	0.001073
GO:0106118	BP	regulation of sterol biosynthetic process	2	0.001073
GO:0060415	BP	muscle tissue morphogenesis	3	0.001087
GO:0072088	BP	nephron epithelium morphogenesis	3	0.001124
GO:0050806	BP	positive regulation of synaptic transmission	5	0.00116
GO:0007405	BP	neuroblast proliferation	3	0.001161
GO:0035584	BP	calcium-mediated signaling using intracellular calcium source	2	0.001179
GO:0045606	BP	positive regulation of epidermal cell differentiation	2	0.001179
GO:0051770	BP	positive regulation of nitric-oxide synthase biosynthetic process	2	0.001179
GO:0060307	BP	regulation of ventricular cardiac muscle cell membrane repolarization	2	0.001179
GO:0072273	BP	metanephric nephron morphogenesis	2	0.001179
GO:0090205	BP	positive regulation of cholesterol metabolic process	2	0.001179
GO:1902992	BP	negative regulation of amyloid precursor protein catabolic process	2	0.001179
GO:0051099	BP	positive regulation of binding	4	0.001198
GO:0014015	BP	positive regulation of gliogenesis	3	0.001199
GO:0045995	BP	regulation of embryonic development	3	0.001199
GO:0051384	BP	response to glucocorticoid	3	0.001199
GO:0045665	BP	negative regulation of neuron differentiation	3	0.001238
GO:0015914	BP	phospholipid transport	3	0.001278
GO:0019915	BP	lipid storage	3	0.001278
GO:0061333	BP	renal tubule morphogenesis	3	0.001278
GO:0010880	BP	regulation of release of sequestered calcium ion into cytosol by sarcoplasmic reticulum	2	0.001289
GO:0031649	BP	heat generation	2	0.001289
GO:0050965	BP	detection of temperature stimulus involved in sensory perception of pain	2	0.001289
GO:0072202	BP	cell differentiation involved in metanephros development	2	0.001289

GO:0120255	BP	olefinic compound biosynthetic process	2	0.001289
GO:1903672	BP	positive regulation of sprouting angiogenesis	2	0.001289
GO:0043087	BP	regulation of GTPase activity	5	0.001351
GO:0034612	BP	response to tumor necrosis factor	4	0.001359
GO:0006835	BP	dicarboxylic acid transport	3	0.001359
GO:0070555	BP	response to interleukin-1	3	0.001359
GO:0035050	BP	embryonic heart tube development	3	0.001401
GO:0048644	BP	muscle organ morphogenesis	3	0.001401
GO:0033630	BP	positive regulation of cell adhesion mediated by integrin	2	0.001404
GO:0034405	BP	response to fluid shear stress	2	0.001404
GO:0036303	BP	lymph vessel morphogenesis	2	0.001404
GO:0040019	BP	positive regulation of embryonic development	2	0.001404
GO:0048485	BP	sympathetic nervous system development	2	0.001404
GO:0099625	BP	ventricular cardiac muscle cell membrane repolarization	2	0.001404
GO:2000810	BP	regulation of bicellular tight junction assembly	2	0.001404
GO:0043491	BP	protein kinase B signaling	4	0.001408
GO:1990138	BP	neuron projection extension	4	0.001408
GO:0061053	BP	somite development	3	0.001444
GO:0022408	BP	negative regulation of cell-cell adhesion	4	0.001458
GO:0043281	BP	regulation of cysteine-type endopeptidase activity involved in apoptotic process	4	0.001458
GO:0061351	BP	neural precursor cell proliferation	4	0.001484
GO:0044344	BP	cellular response to fibroblast growth factor stimulus	3	0.001488
GO:0006706	BP	steroid catabolic process	2	0.001524
GO:0007620	BP	copulation	2	0.001524
GO:0010759	BP	positive regulation of macrophage chemotaxis	2	0.001524
GO:0014808	BP	release of sequestered calcium ion into cytosol by sarcoplasmic reticulum	2	0.001524
GO:0034114	BP	regulation of heterotypic cell-cell adhesion	2	0.001524
GO:0060547	BP	negative regulation of necrotic cell death	2	0.001524
GO:1903306	BP	negative regulation of regulated secretory pathway	2	0.001524
GO:0001570	BP	vasculogenesis	3	0.001577
GO:0010717	BP	regulation of epithelial to mesenchymal transition	3	0.001577
GO:0002690	BP	positive regulation of leukocyte chemotaxis	3	0.001624
GO:0031960	BP	response to corticosteroid	3	0.001624
GO:0071774	BP	response to fibroblast growth factor	3	0.001624
GO:0008361	BP	regulation of cell size	4	0.001643
GO:0008211	BP	glucocorticoid metabolic process	2	0.001649
GO:0045684	BP	positive regulation of epidermis development	2	0.001649
GO:0046685	BP	response to arsenic-containing substance	2	0.001649
GO:0099623	BP	regulation of cardiac muscle cell membrane repolarization	2	0.001649
GO:1902230	BP	negative regulation of intrinsic apoptotic signaling pathway in response to DNA damage	2	0.001649
GO:1902993	BP	positive regulation of amyloid precursor protein catabolic process	2	0.001649
GO:1903514	BP	release of sequestered calcium ion into cytosol by endoplasmic reticulum	2	0.001649
GO:1903975	BP	regulation of glial cell migration	2	0.001649
GO:2000647	BP	negative regulation of stem cell proliferation	2	0.001649

GO:0019226	BP	transmission of nerve impulse	3	0.001671
GO:0042632	BP	cholesterol homeostasis	3	0.001671
GO:0060021	BP	roof of mouth development	3	0.001671
GO:0014910	BP	regulation of smooth muscle cell migration	3	0.001718
GO:0055092	BP	sterol homeostasis	3	0.001718
GO:1904063	BP	negative regulation of cation transmembrane transport	3	0.001718
GO:0048839	BP	inner ear development	4	0.001727
GO:0050796	BP	regulation of insulin secretion	4	0.001727
GO:0043502	BP	regulation of muscle adaptation	3	0.001767
GO:0072091	BP	regulation of stem cell proliferation	3	0.001767
GO:2001237	BP	negative regulation of extrinsic apoptotic signaling pathway	3	0.001767
GO:0003338	BP	metanephros morphogenesis	2	0.001778
GO:0006883	BP	intracellular sodium ion homeostasis	2	0.001778
GO:0033622	BP	integrin activation	2	0.001778
GO:0034368	BP	protein-lipid complex remodeling	2	0.001778
GO:0034369	BP	plasma lipoprotein particle remodeling	2	0.001778
GO:0050961	BP	detection of temperature stimulus involved in sensory perception	2	0.001778
GO:0090335	BP	regulation of brown fat cell differentiation	2	0.001778
GO:0032092	BP	positive regulation of protein binding	3	0.001816
GO:0046330	BP	positive regulation of JNK cascade	3	0.001816
GO:0007188	BP	adenylate cyclase-modulating G protein-coupled receptor signaling pathway	4	0.001844
GO:0017157	BP	regulation of exocytosis	4	0.001874
GO:0021700	BP	developmental maturation	5	0.001887
GO:0048469	BP	cell maturation	4	0.001904
GO:0006887	BP	exocytosis	5	0.001909
GO:0060039	BP	pericardium development	2	0.001912
GO:0060445	BP	branching involved in salivary gland morphogenesis	2	0.001912
GO:0045862	BP	positive regulation of proteolysis	5	0.001953
GO:1903046	BP	meiotic cell cycle process	4	0.001966
GO:0006641	BP	triglyceride metabolic process	3	0.00197
GO:0030510	BP	regulation of BMP signaling pathway	3	0.00197
GO:0050795	BP	regulation of behavior	3	0.00197
GO:0070167	BP	regulation of biomineral tissue development	3	0.00197
GO:0010810	BP	regulation of cell-substrate adhesion	4	0.001997
GO:0014066	BP	regulation of phosphatidylinositol 3-kinase signaling	3	0.002023
GO:0016525	BP	negative regulation of angiogenesis	3	0.002023
GO:0001759	BP	organ induction	2	0.002051
GO:0014829	BP	vascular associated smooth muscle contraction	2	0.002051
GO:0032305	BP	positive regulation of icosanoid secretion	2	0.002051
GO:0034367	BP	protein-containing complex remodeling	2	0.002051
GO:0045187	BP	regulation of circadian sleep/wake cycle, sleep	2	0.002051
GO:0051767	BP	nitric-oxide synthase biosynthetic process	2	0.002051
GO:0051769	BP	regulation of nitric-oxide synthase biosynthetic process	2	0.002051
GO:0060008	BP	Sertoli cell differentiation	2	0.002051

GO:0060444	BP	branching involved in mammary gland duct morphogenesis	2	0.002051
GO:0060571	BP	morphogenesis of an epithelial fold	2	0.002051
GO:0061311	BP	cell surface receptor signaling pathway involved in heart development	2	0.002051
GO:1900027	BP	regulation of ruffle assembly	2	0.002051
GO:0032890	BP	regulation of organic acid transport	3	0.002077
GO:0030518	BP	intracellular steroid hormone receptor signaling pathway	3	0.002132
GO:0071901	BP	negative regulation of protein serine/threonine kinase activity	3	0.002132
GO:0032496	BP	response to lipopolysaccharide	5	0.002183
GO:0006929	BP	substrate-dependent cell migration	2	0.002194
GO:0015732	BP	prostaglandin transport	2	0.002194
GO:0015874	BP	norepinephrine transport	2	0.002194
GO:0033198	BP	response to ATP	2	0.002194
GO:0045932	BP	negative regulation of muscle contraction	2	0.002194
GO:0050995	BP	negative regulation of lipid catabolic process	2	0.002194
GO:0090200	BP	positive regulation of release of cytochrome c from mitochondria	2	0.002194
GO:0010976	BP	positive regulation of neuron projection development	4	0.002226
GO:0032642	BP	regulation of chemokine production	3	0.002244
GO:0045930	BP	negative regulation of mitotic cell cycle	4	0.00226
GO:0008630	BP	intrinsic apoptotic signaling pathway in response to DNA damage	3	0.002301
GO:0003178	BP	coronary sinus valve development	1	0.002311
GO:0003182	BP	coronary sinus valve morphogenesis	1	0.002311
GO:0003243	BP	circumferential growth involved in left ventricle morphogenesis	1	0.002311
GO:0003270	BP	Notch signaling pathway involved in regulation of secondary heart field cardioblast proliferation	1	0.002311
GO:0006542	BP	glutamine biosynthetic process	1	0.002311
GO:0010021	BP	amylopectin biosynthetic process	1	0.002311
GO:0014843	BP	growth factor dependent regulation of skeletal muscle satellite cell proliferation	1	0.002311
GO:0021806	BP	initiation of movement involved in cerebral cortex radial glia guided migration	1	0.002311
GO:0021808	BP	cytosolic calcium signaling involved in initiation of cell movement in glial-mediated radial cell migration	1	0.002311
GO:0031550	BP	positive regulation of brain-derived neurotrophic factor receptor signaling pathway	1	0.002311
GO:0031945	BP	positive regulation of glucocorticoid metabolic process	1	0.002311
GO:0031948	BP	positive regulation of glucocorticoid biosynthetic process	1	0.002311
GO:0032244	BP	positive regulation of nucleoside transport	1	0.002311
GO:0032900	BP	negative regulation of neurotrophin production	1	0.002311
GO:0035589	BP	G protein-coupled purinergic nucleotide receptor signaling pathway	1	0.002311
GO:0035724	BP	CD24 biosynthetic process	1	0.002311
GO:0035921	BP	desmosome disassembly	1	0.002311
GO:0036309	BP	protein localization to M-band	1	0.002311
GO:0036371	BP	protein localization to T-tubule	1	0.002311
GO:0060843	BP	venous endothelial cell differentiation	1	0.002311
GO:0061369	BP	negative regulation of testicular blood vessel morphogenesis	1	0.002311
GO:0071831	BP	intermediate-density lipoprotein particle clearance	1	0.002311
GO:0090731	BP	cellular response to very-low-density lipoprotein particle stimulus	1	0.002311
GO:0097497	BP	blood vessel endothelial cell delamination	1	0.002311
GO:0098694	BP	regulation of synaptic vesicle budding from presynaptic endocytic zone membrane	1	0.002311

GO:0099083	BP	retrograde trans-synaptic signaling by neuropeptide, modulating synaptic transmission	1	0.002311
GO:0150200	BP	regulation of transport across blood-brain barrier	1	0.002311
GO:0150201	BP	positive regulation of transport across blood-brain barrier	1	0.002311
GO:0150202	BP	negative regulation of transport across blood-brain barrier	1	0.002311
GO:1900085	BP	negative regulation of peptidyl-tyrosine autophosphorylation	1	0.002311
GO:1901627	BP	negative regulation of postsynaptic membrane organization	1	0.002311
GO:1902618	BP	cellular response to fluoride	1	0.002311
GO:1902999	BP	negative regulation of phospholipid efflux	1	0.002311
GO:1903000	BP	regulation of lipid transport across blood-brain barrier	1	0.002311
GO:1903001	BP	negative regulation of lipid transport across blood-brain barrier	1	0.002311
GO:1903002	BP	positive regulation of lipid transport across blood-brain barrier	1	0.002311
GO:1903165	BP	response to polycyclic arene	1	0.002311
GO:1903166	BP	cellular response to polycyclic arene	1	0.002311
GO:1904445	BP	negative regulation of establishment of Sertoli cell barrier	1	0.002311
GO:1904681	BP	response to 3-methylcholanthrene	1	0.002311
GO:1904682	BP	cellular response to 3-methylcholanthrene	1	0.002311
GO:1904998	BP	negative regulation of leukocyte adhesion to arterial endothelial cell	1	0.002311
GO:1905630	BP	response to glyceraldehyde	1	0.002311
GO:1905631	BP	cellular response to glyceraldehyde	1	0.002311
GO:1905853	BP	regulation of heparan sulfate binding	1	0.002311
GO:1905855	BP	positive regulation of heparan sulfate binding	1	0.002311
GO:1905858	BP	regulation of heparan sulfate proteoglycan binding	1	0.002311
GO:1905860	BP	positive regulation of heparan sulfate proteoglycan binding	1	0.002311
GO:1905890	BP	regulation of cellular response to very-low-density lipoprotein particle stimulus	1	0.002311
GO:2000066	BP	positive regulation of cortisol biosynthetic process	1	0.002311
GO:2000180	BP	negative regulation of androgen biosynthetic process	1	0.002311
GO:2000438	BP	negative regulation of monocyte extravasation	1	0.002311
GO:2000559	BP	regulation of CD24 production	1	0.002311
GO:2000560	BP	positive regulation of CD24 production	1	0.002311
GO:2000896	BP	amylopectin metabolic process	1	0.002311
GO:2001139	BP	negative regulation of phospholipid transport	1	0.002311
GO:0003085	BP	negative regulation of systemic arterial blood pressure	2	0.002342
GO:0016048	BP	detection of temperature stimulus	2	0.002342
GO:0050802	BP	circadian sleep/wake cycle, sleep	2	0.002342
GO:0051446	BP	positive regulation of meiotic cell cycle	2	0.002342
GO:0060037	BP	pharyngeal system development	2	0.002342
GO:0099622	BP	cardiac muscle cell membrane repolarization	2	0.002342
GO:0030516	BP	regulation of axon extension	3	0.00236
GO:0035282	BP	segmentation	3	0.00236
GO:0001505	BP	regulation of neurotransmitter levels	4	0.002401
GO:0034766	BP	negative regulation of monoatomic ion transmembrane transport	3	0.002419
GO:0002526	BP	acute inflammatory response	3	0.002479
GO:0045446	BP	endothelial cell differentiation	3	0.002479
GO:1904659	BP	glucose transmembrane transport	3	0.002479

GO:0001945	BP	lymph vessel development	2	0.002494
GO:0032958	BP	inositol phosphate biosynthetic process	2	0.002494
GO:0042749	BP	regulation of circadian sleep/wake cycle	2	0.002494
GO:0060977	BP	coronary vasculature morphogenesis	2	0.002494
GO:0070296	BP	sarcoplasmic reticulum calcium ion transport	2	0.002494
GO:0071548	BP	response to dexamethasone	2	0.002494
GO:0008645	BP	hexose transmembrane transport	3	0.00254
GO:0045666	BP	positive regulation of neuron differentiation	3	0.00254
GO:0008406	BP	gonad development	4	0.002547
GO:0030301	BP	cholesterol transport	3	0.002602
GO:0042572	BP	retinol metabolic process	2	0.002651
GO:0048668	BP	collateral sprouting	2	0.002651
GO:0070633	BP	transepithelial transport	2	0.002651
GO:1901797	BP	negative regulation of signal transduction by p53 class mediator	2	0.002651
GO:0045137	BP	development of primary sexual characteristics	4	0.002699
GO:0002237	BP	response to molecule of bacterial origin	5	0.002703
GO:0015749	BP	monosaccharide transmembrane transport	3	0.002729
GO:0015908	BP	fatty acid transport	3	0.002729
GO:0042542	BP	response to hydrogen peroxide	3	0.002729
GO:0090100	BP	positive regulation of transmembrane receptor protein serine/threonine kinase signaling pathway	3	0.002729
GO:2000116	BP	regulation of cysteine-type endopeptidase activity	4	0.002738
GO:0022404	BP	molting cycle process	3	0.002794
GO:0022405	BP	hair cycle process	3	0.002794
GO:0032602	BP	chemokine production	3	0.002794
GO:0010758	BP	regulation of macrophage chemotaxis	2	0.002812
GO:0022410	BP	circadian sleep/wake cycle process	2	0.002812
GO:0032303	BP	regulation of icosanoid secretion	2	0.002812
GO:0040036	BP	regulation of fibroblast growth factor receptor signaling pathway	2	0.002812
GO:0044319	BP	wound healing, spreading of cells	2	0.002812
GO:0045742	BP	positive regulation of epidermal growth factor receptor signaling pathway	2	0.002812
GO:0090505	BP	epiboly involved in wound healing	2	0.002812
GO:1901099	BP	negative regulation of signal transduction in absence of ligand	2	0.002812
GO:2000193	BP	positive regulation of fatty acid transport	2	0.002812
GO:2001240	BP	negative regulation of extrinsic apoptotic signaling pathway in absence of ligand	2	0.002812
GO:0007498	BP	mesoderm development	3	0.002859
GO:0008585	BP	female gonad development	3	0.002859
GO:0015918	BP	sterol transport	3	0.002859
GO:0048709	BP	oligodendrocyte differentiation	3	0.002859
GO:0007281	BP	germ cell development	5	0.002875
GO:0002052	BP	positive regulation of neuroblast proliferation	2	0.002978
GO:0050951	BP	sensory perception of temperature stimulus	2	0.002978
GO:0090504	BP	epiboly	2	0.002978
GO:0051588	BP	regulation of neurotransmitter transport	3	0.002994
GO:0042383	CC	sarcolemma	10	5.29E-12

GO:0045121	CC	membrane raft	12	8.92E-11
GO:0098857	CC	membrane microdomain	12	9.18E-11
GO:0030315	CC	T-tubule	7	2.69E-10
GO:0044853	CC	plasma membrane raft	7	2.63E-08
GO:0005901	CC	caveola	6	1.01E-07
GO:0045211	CC	postsynaptic membrane	9	1.07E-07
GO:0043204	CC	perikaryon	6	1.54E-06
GO:0150034	CC	distal axon	8	3.67E-06
GO:0043679	CC	axon terminus	6	7.38E-06
GO:0048786	CC	presynaptic active zone	5	9.46E-06
GO:0044306	CC	neuron projection terminus	6	1.33E-05
GO:0002095	CC	caveolar macromolecular signaling complex	2	1.57E-05
GO:0014704	CC	intercalated disc	4	1.78E-05
GO:0042734	CC	presynaptic membrane	6	1.87E-05
GO:0043235	CC	receptor complex	7	5.11E-05
GO:0044291	CC	cell-cell contact zone	4	5.7E-05
GO:0009925	CC	basal plasma membrane	6	5.86E-05
GO:0043195	CC	terminal bouton	4	6.48E-05
GO:0045178	CC	basal part of cell	6	9.06E-05
GO:0045098	CC	type III intermediate filament	2	0.000109
GO:0030018	CC	Z disc	4	0.000244
GO:0097179	CC	protease inhibitor complex	2	0.000285
GO:0016323	CC	basolateral plasma membrane	5	0.000329
GO:0016324	CC	apical plasma membrane	6	0.00035
GO:0031674	CC	I band	4	0.000359
GO:0005641	CC	nuclear envelope lumen	2	0.000403
GO:0005916	CC	fascia adherens	2	0.000403
GO:0048787	CC	presynaptic active zone membrane	3	0.000507
GO:0014069	CC	postsynaptic density	6	0.000548
GO:0032279	CC	asymmetric synapse	6	0.000691
GO:0001518	CC	voltage-gated sodium channel complex	2	0.000698
GO:0019897	CC	extrinsic component of plasma membrane	4	0.000808
GO:0099572	CC	postsynaptic specialization	6	0.000899
GO:0005788	CC	endoplasmic reticulum lumen	3	0.001159
GO:0030017	CC	sarcomere	4	0.001194
GO:0005791	CC	rough endoplasmic reticulum	3	0.001356
GO:1904090	CC	peptidase inhibitor complex	2	0.001402
GO:0005125	MF	cytokine activity	8	6.11E-08
GO:0097110	MF	scaffold protein binding	5	2E-06
GO:0001614	MF	purinergic nucleotide receptor activity	3	1.11E-05
GO:0016502	MF	nucleotide receptor activity	3	1.11E-05
GO:0001621	MF	G protein-coupled ADP receptor activity	2	1.57E-05
GO:0005539	MF	glycosaminoglycan binding	6	1.73E-05
GO:0050998	MF	nitric-oxide synthase binding	3	1.75E-05

GO:0001091	MF	RNA polymerase II general transcription initiation factor binding	3	2.93E-05
GO:0008201	MF	heparin binding	5	5.19E-05
GO:0001093	MF	TFIIB-class transcription factor binding	2	7.82E-05
GO:0001609	MF	G protein-coupled adenosine receptor activity	2	7.82E-05
GO:0090554	MF	phosphatidylcholine floppase activity	2	7.82E-05
GO:0140328	MF	floppase activity	2	0.000109
GO:0001664	MF	G protein-coupled receptor binding	6	0.00011
GO:0005262	MF	calcium channel activity	4	0.000128
GO:0070888	MF	E-box binding	3	0.000166
GO:0140359	MF	ABC-type transporter activity	3	0.000177
GO:1990782	MF	protein tyrosine kinase binding	4	0.000216
GO:0015085	MF	calcium ion transmembrane transporter activity	4	0.000229
GO:0099106	MF	ion channel regulator activity	4	0.000296
GO:0045028	MF	G protein-coupled purinergic nucleotide receptor activity	2	0.000341
GO:0140296	MF	general transcription initiation factor binding	3	0.000348
GO:0016247	MF	channel regulator activity	4	0.000376
GO:0008083	MF	growth factor activity	4	0.000396
GO:0001098	MF	basal transcription machinery binding	3	0.000422
GO:0001099	MF	basal RNA polymerase II transcription machinery binding	3	0.000422
GO:0044325	MF	transmembrane transporter binding	4	0.000427
GO:0005216	MF	monoatomic ion channel activity	6	0.000478
GO:1901681	MF	sulfur compound binding	5	0.000584
GO:0005200	MF	structural constituent of cytoskeleton	3	0.000624
GO:0008559	MF	ABC-type xenobiotic transporter activity	2	0.000697
GO:0022839	MF	monoatomic ion gated channel activity	5	0.000741
GO:0015562	MF	efflux transmembrane transporter activity	2	0.000783
GO:0022836	MF	gated channel activity	5	0.000785
GO:0046982	MF	protein heterodimerization activity	5	0.000785
GO:1990841	MF	promoter-specific chromatin binding	3	0.000788
GO:0005126	MF	cytokine receptor binding	5	0.00082
GO:0005261	MF	monoatomic cation channel activity	5	0.00082
GO:0015267	MF	channel activity	6	0.000865
GO:0022803	MF	passive transmembrane transporter activity	6	0.000865

Table S4 KEGG analysis of potential therapeutic targets

ID	Term	Count	geneID	pvalue
mmu04020	Calcium signaling pathway - Mus musculus (house mouse)	9	Gdnf/Cacna1c/Ntrk2/Cysltr1/Plcb1/Cacna1h/Fgf2/Htr2a/Ednra	1.99E-05
mmu05418	Fluid shear stress and atherosclerosis - Mus musculus (house mouse)	7	Cav1/Gsto1/Trpv4/Hmox1/Plat/Ccl2/Il1a	2.91E-05
mmu04924	Renin secretion - Mus musculus (house mouse)	5	Cacna1c/Plcb1/Adora1/Ednra/Pde3a	9.25E-05

mmu05207	Chemical carcinogenesis - receptor activation - Mus musculus (house mouse)	7	Gsto1/Cyp1b1/Ahr/Cacna1c/Klf4/Fgf2/Esr1	0.000422
mmu04929	GnRH secretion - Mus musculus (house mouse)	4	Cacna1c/Plcb1/Cacna1h/Spp1	0.00056
mmu04022	cGMP-PKG signaling pathway - Mus musculus (house mouse)	6	Cacna1c/Plcb1/Adora1/Prkg1/Ednra/Pde3a	0.000586
mmu05417	Lipid and atherosclerosis - Mus musculus (house mouse)	6	Abcg1/Ccl2/Plcb1/Tnfsf10/Casp1/Olr1	0.00195
mmu04010	MAPK signaling pathway - Mus musculus (house mouse)	7	Gdnf/Cacna1c/Bdnf/Ntrk2/Cacna1h/Fgf2/Il1a	0.002115
mmu04024	cAMP signaling pathway - Mus musculus (house mouse)	6	Cacna1c/Bdnf/Hcar2/Adora1/Ednra/Pde3a	0.002289
mmu04934	Cushing syndrome - Mus musculus (house mouse)	5	Wnt4/Ahr/Cacna1c/Plcb1/Cacna1h	0.002942
mmu04713	Circadian entrainment - Mus musculus (house mouse)	4	Cacna1c/Plcb1/Cacna1h/Prkg1	0.003012
mmu02010	ABC transporters - Mus musculus (house mouse)	3	Abcg1/Abcb1a/Abcg2	0.003812
mmu04919	Thyroid hormone signaling pathway - Mus musculus (house mouse)	4	Wnt4/Notch1/Plcb1/Esr1	0.005981
mmu04611	Platelet activation - Mus musculus (house mouse)	4	P2ry1/Plcb1/Prkg1/P2ry12	0.0069
mmu05205	Proteoglycans in cancer - Mus musculus (house mouse)	5	Wnt4/Cav1/Fgf2/Ank2/Esr1	0.00778
mmu04750	Inflammatory mediator regulation of TRP channels - Mus musculus (house mouse)	4	Trpv4/Plcb1/Htr2a/Trpv2	0.007908
mmu04927	Cortisol synthesis and secretion - Mus musculus (house mouse)	3	Cacna1c/Plcb1/Cacna1h	0.009442
mmu04621	NOD-like receptor signaling pathway - Mus musculus (house mouse)	5	Txnip/Ccl2/Plcb1/Casp1/Trpv2	0.009827
mmu04270	Vascular smooth muscle contraction - Mus musculus (house mouse)	4	Cacna1c/Plcb1/Prkg1/Ednra	0.011235
mmu05224	Breast cancer - Mus musculus (house mouse)	4	Wnt4/Notch1/Fgf2/Esr1	0.012049
mmu04540	Gap junction - Mus musculus (house mouse)	3	Plcb1/Prkg1/Htr2a	0.015264
mmu05412	Arrhythmogenic right ventricular cardiomyopathy - Mus musculus (house mouse)	3	Cacna1c/Cdh2/Des	0.015264
mmu05323	Rheumatoid arthritis - Mus musculus (house mouse)	3	Ccl2/Il1a/Ccl20	0.015742
mmu04727	GABAergic synapse - Mus musculus (house mouse)	3	Gabrp/Glul/Cacna1c	0.017226
mmu04742	Taste transduction - Mus musculus (house mouse)	3	P2ry1/Cacna1c/Plcb1	0.018258

mmu05032	Morphine addiction - Mus musculus (house mouse)	3	Gabrp/Adora1/Pde3a	0.018258
mmu04061	Viral protein interaction with cytokine and cytokine receptor - Mus musculus (house mouse)	3	Ccl2/Tnfsf10/Ccl20	0.019869
mmu05164	Influenza A - Mus musculus (house mouse)	4	Ccl2/Tnfsf10/Casp1/Il1a	0.020713
mmu04217	Necroptosis - Mus musculus (house mouse)	4	Glul/Tnfsf10/Casp1/Il1a	0.022314
mmu00250	Alanine, aspartate and glutamate metabolism - Mus musculus (house mouse)	2	Glul/Aspa	0.023285
mmu04933	AGE-RAGE signaling pathway in diabetic complications - Mus musculus (house mouse)	3	Ccl2/Plcb1/Il1a	0.023321
mmu04218	Cellular senescence - Mus musculus (house mouse)	4	Trpv4/Ccna2/Ccnb1/Il1a	0.02442
mmu04925	Aldosterone synthesis and secretion - Mus musculus (house mouse)	3	Cacna1c/Plcb1/Cacna1h	0.024539
mmu04080	Neuroactive ligand-receptor interaction - Mus musculus (house mouse)	6	Gabrp/P2ry1/Cysltr1/Adora1/Htr2a/Ednra	0.030319
mmu04060	Cytokine-cytokine receptor interaction - Mus musculus (house mouse)	5	Bmp7/Ccl2/Tnfsf10/Il1a/Ccl20	0.03255
mmu04724	Glutamatergic synapse - Mus musculus (house mouse)	3	Glul/Cacna1c/Plcb1	0.032564
mmu04930	Type II diabetes mellitus - Mus musculus (house mouse)	2	Cacna1c/Slc2a4	0.034238
mmu05206	MicroRNAs in cancer - Mus musculus (house mouse)	5	Cyp1b1/Notch1/Hmox1/Abcb1a/Vim	0.036366
mmu04071	Sphingolipid signaling pathway - Mus musculus (house mouse)	3	Acer2/Plcb1/Adora1	0.040983
mmu04068	FoxO signaling pathway - Mus musculus (house mouse)	3	Tnfsf10/Ccnb1/Slc2a4	0.045996
mmu04923	Regulation of lipolysis in adipocytes - Mus musculus (house mouse)	2	Adora1/Prkg1	0.046799
mmu04726	Serotonergic synapse - Mus musculus (house mouse)	3	Cacna1c/Plcb1/Htr2a	0.048612

Table S5 Ranking of variable importance as identified by the random forest model

Rank	Gene	MeanDecreaseGini
1	Esr1	0.410714286
2	Klf4	0.285714286
3	Sulf1	0.285714286
4	Aldh1a1	0.285714286
5	Mmp10	0.267857143
6	Snai2	0.267857143
7	Ccl2	0.267857143
8	Abcg1	0.267857143
9	Plcb1	0.267857143
10	Ednra	0.267857143
11	P2ry12	0.267857143
